# The GPCR repertoire in the demosponge *Amphimedon queenslandica*: insights into the GPCR system at the early divergence of animals

**DOI:** 10.1186/s12862-014-0270-4

**Published:** 2014-12-21

**Authors:** Arunkumar Krishnan, Rohit Dnyansagar, Markus Sällman Almén, Michael J Williams, Robert Fredriksson, Narayanan Manoj, Helgi B Schiöth

**Affiliations:** Department of Neuroscience, Functional Pharmacology, Uppsala University, Biomedical Center, Box 593, 75 124 Uppsala, Sweden; Department of Biotechnology, Indian Institute of Technology Madras, Chennai, 600036 India

**Keywords:** Neurotransmitters, G protein-coupled receptors, Adhesion, Signal transduction, Porifera, Eumetazoa

## Abstract

**Background:**

G protein-coupled receptors (GPCRs) play a central role in eukaryotic signal transduction. However, the GPCR component of this signalling system, at the early origins of metazoans is not fully understood. Here we aim to identify and classify GPCRs in *Amphimedon queenslandica* (sponge), a member of an earliest diverging metazoan lineage (Porifera). Furthermore, phylogenetic comparisons of sponge GPCRs with eumetazoan and bilaterian GPCRs will be essential to our understanding of the GPCR system at the roots of metazoan evolution.

**Results:**

We present a curated list of 220 GPCRs in the sponge genome after excluding incomplete sequences and false positives from our initial dataset of 282 predicted GPCR sequences obtained using Pfam search. Phylogenetic analysis reveals that the sponge genome contains members belonging to four of the five major GRAFS families including *Glutamate* (33), *Rhodopsin* (126), *Adhesion* (40) and *Frizzled* (3). Interestingly, the sponge *Rhodopsin* family sequences lack orthologous relationships with those found in eumetazoan and bilaterian lineages, since they clustered separately to form sponge specific groups in the phylogenetic analysis. This suggests that sponge *Rhodopsins* diverged considerably from that found in other basal metazoans. A few sponge *Adhesion*s clustered basal to *Adhesion* subfamilies commonly found in most vertebrates, suggesting some *Adhesion* subfamilies may have diverged prior to the emergence of Bilateria. Furthermore, at least eight of the sponge *Adhesion* members have a hormone binding motif (HRM domain) in their N-termini, although hormones have yet to be identified in sponges. We also phylogenetically clarified that sponge has homologs of metabotropic glutamate (mGluRs) and GABA receptors.

**Conclusion:**

Our phylogenetic comparisons of sponge GPCRs with other metazoan genomes suggest that sponge contains a significantly diversified set of GPCRs. This is evident at the family/subfamily level comparisons for most GPCR families, in particular for the *Rhodopsin* family of GPCRs. In summary, this study provides a framework to perform future experimental and comparative studies to further verify and understand the roles of GPCRs that predates the divergence of bilaterian and eumetazoan lineages.

**Electronic supplementary material:**

The online version of this article (doi:10.1186/s12862-014-0270-4) contains supplementary material, which is available to authorized users.

## Background

The G protein-coupled receptor (GPCR) superfamily is one of the largest families of integral transmembrane proteins in vertebrates and plays a dominant role in signal transduction in most eukaryotes. GPCRs, which mediate most of the cellular responses through hormones, neurotransmitters and environmental stimulants are thus major drug targets, with approximately 36% of current clinical drugs targeting these receptors [1,2]. In humans, there are around 800 genes coding for GPCRs, and we earlier classified them into five main GRAFS families: G*lutamate*, *Rhodopsin*, *Adhesion*, *Frizzled* and *Secretin* [3,4]. Subsequent GPCR mining studies have suggested that GRAFS families are present in most bilaterian species [5-8]. In addition, our earlier studies demonstrated that four of the five GRAFS families (excluding *Secretin*, which evolved after the divergence of cnidarians) are found in basal fungi, indicating that the *Glutamate, Rhodopsin, Adhesion,* and *Frizzled* families evolved before the divergence of metazoan lineages [9].

Although the four GRAFS families first evolved in the basal fungi, only a few sequences were unambiguous homologs of metazoan representatives [9]. For example, only a few homologues of the *Rhodopsin* family were found in basal fungi and *Rhodopsin* GPCRs were not found in choanoflagellates (*Monosiga brevicollis* and *Salpingoeca rosetta*) and filasterean *Capsaspora owczarzaki* [9,10]. Moreover, these closest metazoan relatives are limited to only a few genes coding for *Adhesion* and *Glutamate* GPCR families. These observations clearly indicate that the first large expansions of *Rhodopsin* GPCRs, as well as other families of GPCRs, occurred at the early origins of metazoans. This model is well supported by the recent genome release of *Amphimedon queenslandica* (hereafter referred to as sponge), which belongs to one of the earliest diverging and oldest surviving phyletic branches of Metazoa. The draft genome as well as the transcriptome profiling of sponge indicated the presence of several *Rhodopsin*-like GPCRs and an overall count of more than 200 GPCRs, including *Adhesion* and *Glutamate* family GPCRs [11,12]. Additional studies on some specific subsets of sponge GPCRs such as *Glutamate* [13] and *Frizzled* [14] provided further insights into the GPCR component in sponge. Taken together this suggests that the last common ancestor of metazoans possessed a complex GPCR system, perhaps with expansions within the *Rhodopsin* family in comparison to pre-metazoan lineages like Choanoflagellata [15] and Filasterea [16]. Furthermore, genome data of species that diverged after sponges provided valuable insights into the evolution of the GPCR superfamily. Previous mining of GPCRs in a cnidarian, *Nematostella vectensis* and a placozoan, *Trichoplax adhaerens* revealed that these pre-bilaterian metazoans contained a large GPCR repertoire with 890 and 420 GPCR coding genes, respectively [17,18].

Although several studies including genome-wide analysis of the sponge demonstrated the presence of several GPCRs, a comprehensive overview of sponge GPCR families is still lacking and their relationship to the versions found in eumetazoans and bilaterians is largely unknown. This is important because sponges and the eumetazoans (*Nematostella* and *Trichoplax*) are known to lack most of cell types found in bilaterians. For example, sponges are simple pore bearing animals that lack gut, a nervous system and muscle, but constitute an internal network of canals and ciliated choanocyte chambers that pump water to extract food [19-21]. Placozoans (*Trichoplax*) are flat animals consisting of a lower and upper epithelium, which sandwich layers of multinucleated fibre cells [17,22]. Similarly, nerves, sensory cells and muscle cells are apparently absent in placozoans. In contrast, *Nematostella* is regarded as one of the first animals possessing a nervous system. In *Nematostella*, an ectodermal and endodermal nerve net constituting of a simple and diffuse nervous system runs throughout the animal’s body [23,24].

In order to better understand the components of the GPCR system and its evolution at the early origins of Metazoa, we aimed to curate a complete set of GPCRs in sponge, as well as provide a comparative analysis with GPCRs found in eumetazoans (*Nematostella* and *Trichoplax*) and bilaterians (humans and sea urchin; *Strongylocentrotus purpuratus*). Utilising the sponge genome, we sought to answer questions such as, 1) does one of the most ancient metazoan lineage have orthologs of mammalian GPCRs, 2) do sponges hold mammalian-like subfamily level classifications for each major GRAFS families, 3) are sponge GPCRs orthologous to those found in cnidarians and other pre-bilaterian lineages.

## Results

### Identification and classification of GPCRs in sponge

In order to generate a complete set of sponge GPCRs, we aligned the sponge proteome with Hidden Markov Models (HMM) of the 14831 families contained within the Pfam database (version 27). We retrieved all sequences that contained the Pfam domains corresponding to the various GPCR families (see Methods). This initial screen identified 282 GPCR sequences belonging to the GPCR_A Pfam clan (CL0192). These numbers are similar and comparable with previous studies where sponge GPCR sequences were identified [10-12]. However, this initial list of GPCRs included fragments and possibly some false positives, and thus had to be refined before performing phylogenetic analysis to obtain stable and consistent topologies. Therefore, we examined these 282 GPCRs for the presence of seven transmembrane (TM) helices using HMMTOP and Phobius servers. To remove fragments and false positives we excluded the sequences having less than five or more than eight TM regions from the final dataset. To cross verify the list of sponge GPCRs, we aligned each putative sponge GPCR sequence with our tagged human GPCRs using the standalone BLASTP program (data not shown). Such stepwise processes led to the verification and categorisation of a final dataset containing 220 GPCRs. A majority of these were categorised into four of the five main GRAFS families, including 126 *Rhodopsin* (7tm_1), 40 *Adhesion* (7tm_2), 33 *Glutamate* (7tm_3) and three *Frizzled* receptors. However, we did not find *Secretin* family receptors in the sponge genome. It must be mentioned that an earlier report suggested that sponge has *Secretin* family GPCRs [12] possibly due to the presence of the HRM (hormone receptor motif) domain in their N-termini, which is a usual characteristic of *Secretin* GPCRs [4,25]. However, *Secretin* family GPCRs are mostly activated by peptide hormones, which to date have not been identified in the sponge genome [11]. Our finding that the sponge genome lack *Secretin*s is also consistent with earlier studies which proposed that *Secretin* family descended from the *Adhesion* family after the split of the cnidarians from other bilaterians and that the *Secretin* GPCRs are a bilaterian innovation [26,27]. Considering that *Adhesion* and *Secretin* families belong to the same class of GPCRs (Class B) and encode a 7tm_2 transmembrane domain, it is sometimes difficult to distinguish between the families and they can be wrongly assigned. Since *Adhesion* is a parent family to *Secretin* GPCRs and due to the lack of experimental support for the presence of *Secretin* GPCR activity in sponges we here label these class B (7tm_2) receptors as *Adhesion* family receptors. Nevertheless, the presence of an HRM domain in these *Adhesion* GPCRs is intriguing and should prompt further experimental verifications to provide evidence for GPCR mediated hormonal activity in sponges.

Interestingly, in addition to the GRAFS families, we identified 14 cyclic AMP-like receptors (Dicty_CAR; PF05462), two intimal thickness-related-like receptors (PF06814), and one lung-7TM-like receptor (PF06814) in the sponge genome. Moreover, we identified a putative homolog of GPR143 (PF02101), which in humans and other mammals is associated with ocular albinism. In summary, the proportion of sponge GPCRs to the genome size is comparable to several other metazoans and that it also constitutes a large expansion within the *Rhodopsin* family [11,13]. The complete set of sponge GPCR sequences identified in this study is available in FASTA format (see Additional file 1). It must be noted here that the numbers provided in this study may vary from future predictions using subsequent genome assemblies of *Amphimedon*, which may provide better resolution of the fragmentary sequences/regions. To avoid possible confusion in subsequent paragraphs, a whole family is denoted using the corresponding family name in italics with an initial capital letter (*Rhodopsin*), while the homologs/members of a particular family are denoted as *Rhodopsins* or *Adhesions* or *Adhesion*-like receptors.

### Phylogenetic verification of GRAFS topological classification

**Figure 1:**
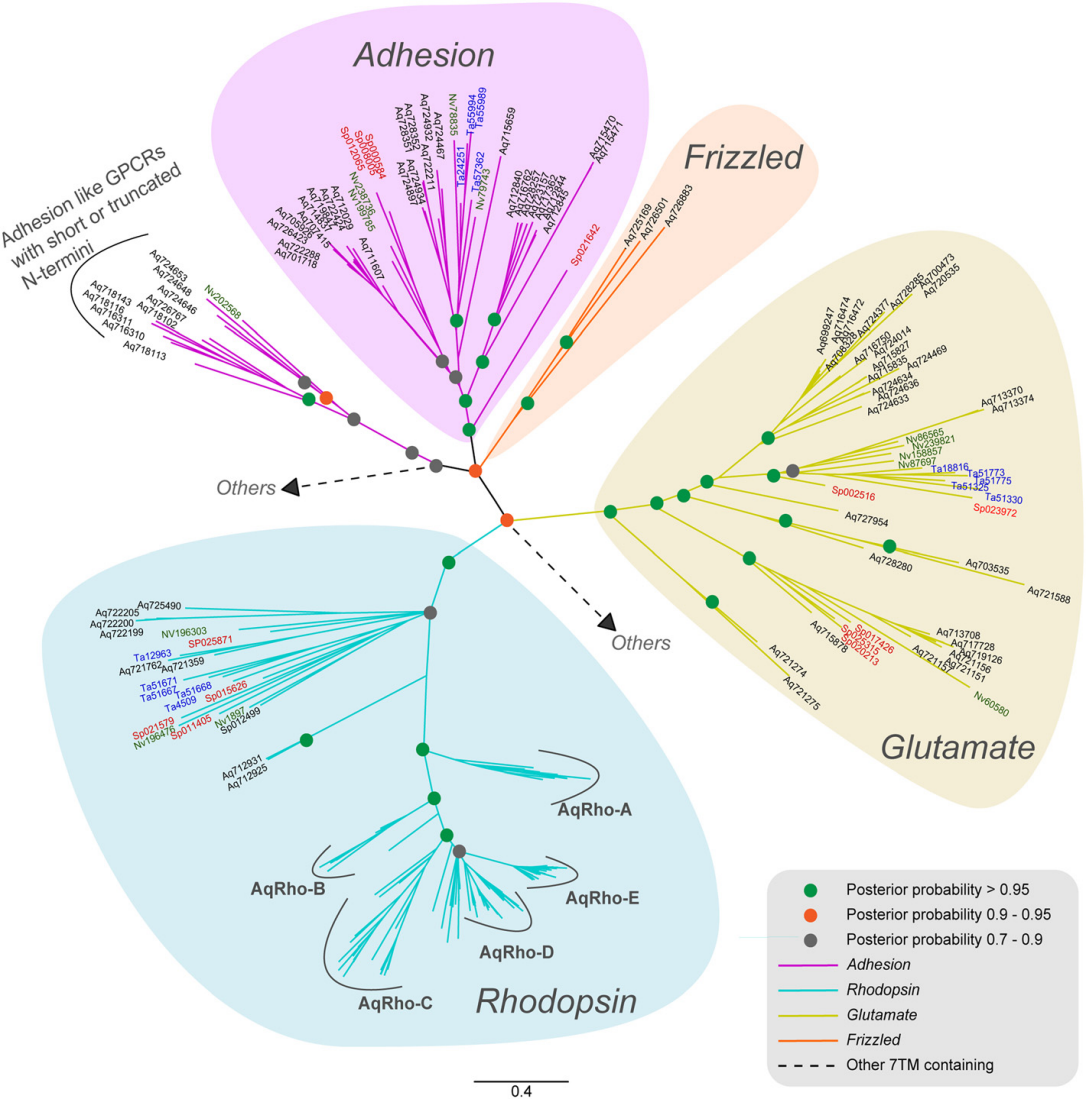
**Phylogenetic relationships of GPCRs identified in the sponge genome.** The tree topology shows distinct phylogenetic clusters belonging to the *Glutamate*, *Rhodopsin*, *Adhesion*, and *Frizzled* families of the GRAFS classification system. The tree also includes a few representatives of GPCR families from other genomes. The edges are colored according to the families, while the accession IDs are colored differently for each species. The illustration shows putative *Adhesion* like GPCRs that lacks the conventional long N-termini. However, these *Adhesion* GPCRs contain the characteristic 7tm_2 domain and placed basal to the major node that contains all the other *Adhesions* included in the phylogenetic tree making. Sponge specific *Rhodopsin* clusters (*AqRho-A* to *AqRho-E*) within the major *Rhodopsin* cluster are highlighted.

The human GPCR repertoire can be classified into five main groups (*Glutamate*, *Rhodopsin*, *Adhesion*, *Frizzled* and *Secretin*; GRAFS) based on phylogenetic analysis [4]. Subsequent comparative phylogenetic studies in several vertebrate and invertebrate species have supported this classification system and established that GPCRs indeed formed distinct clusters corresponding to its five main families [6,8,28]. To investigate whether the GPCRs identified in sponge also exhibit distinct phylogenetic clusters corresponding to the GRAF classes (excluding *Secretin*, which is absent in sponges), we performed a Bayesian phylogenetic analysis using all sponge GPCR sequences. To test the robustness of the topology as well as to resolve the orthology relationships between the members in sponge and other bilaterians and pre-bilaterians, we expanded the dataset to include a few representative sequences from the *Trichoplax, Nematostella* and sea urchin GPCRs. This set contained equal proportion of representatives from *Rhodopsin, Glutamate* and *Adhesion* GPCR families. The overall unrooted topology indicated that the *Rhodopsin*, *Glutamate* and *Adhesion* GPCRs descend into separate and distinct clusters, whereas *Frizzled* GPCRs were placed basal to the *Adhesion* GPCR node (Figure 1).

### *Rhodopsin* receptor family

The human *Rhodopsin* family GPCRs are classified into four major groups termed α-, β-, γ-, and δ that are divided into 13 major subfamilies. Some of these 13 subfamilies like amine and peptide binding *Rhodopsin* family receptors are present and seem fairly conserved in most of the analysed bilaterians [4,6-8,28]. Similarly, in order to categorize sponge *Rhodopsins* and explore their similarity to those found in other species, we aligned each sponge *Rhodopsin* family sequence against a database of GPCRs containing complete repertories from human, sea urchin, *Trichoplax* and *Nematostella*. The entire list of blast hits are provided in Additional file 2. Furthermore, we performed BLAST searches against manually annotated and reviewed *Rhodopsin* (7tm_1) GPCRs obtained from the Swiss-Prot database (available in Additional file 3). This reviewed list of *Rhodopsin* GPCRs included most of the GPCRs from well characterised vertebrates, as well as from several well-known invertebrate model organisms. From BLASTP search results we were unable to classify most of the sponge *Rhodopsin* family sequences into any of the 13 known *Rhodopsin-*like GPCRs subfamilies. This is because sponge *Rhodopsins* failed to satisfy our classification criteria that at least four of the first five hits must be from the same subfamily. However, a few sponge *Rhodopsin*s had their best aligned hits (E-values ranging from e-10 to e-20) to beta-adrenergic receptors, serotonin and opsin family receptors, among others (see Additional file 3). These BLASTP results were subsequently verified using Bayesian and Maximum Likelihood (ML) based phylogenetic analysis.

**Figure 2:**
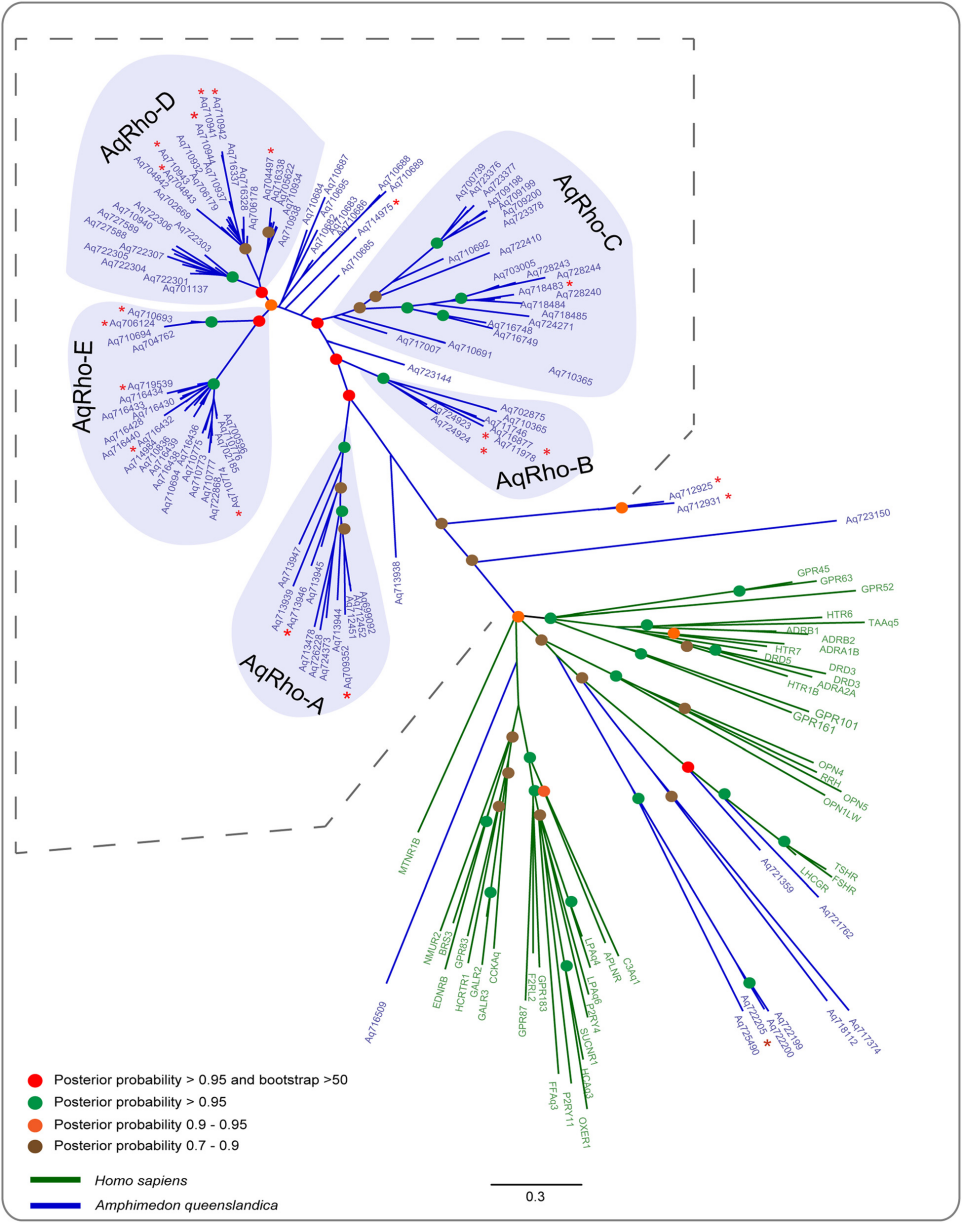
**Phylogenetic tree showing relationships between** ***Rhodopsin*** **family GPCRs in sponge and human genomes.** The tree topology was inferred from Bayesian analysis with a gamma correction using MrBayes software. The phylogenetic tree is based only on the transmembrane region. The MCMC analysis was used to test the robustness of the nodes and was supported by a non-parametric bootstrap analysis with 500 replicates. The edges corresponding to human *Rhodopsin* family GPCRs are highlighted in green. Edges containing sponge *Rhodopsins* are highlighted in blue. Sponge specific clusters and are labeled as *AqRho*-A – *AqRho*-E (where Aq stands for *Amphimedon queenslandica*, Rho for *Rhodopsin* like GPCRs and A to E represent the distinct clusters in the phylogenetic tree). The values indicated at major branches are posterior probability values from Bayesian analysis and percentage bootstrap values from Maximum likelihood analysis. Red asterisk symbol denotes sequences that have at least four of the top five hits as beta-adrenergic, serotonin and opsin family receptors (see Additional file 3) in our blast search. However, they did not seem to form a coherent group with the human *Rhodopsins*. Scale bars indicate phylogenetic distance as number of substitutions per site. Phylogenetic relationships between sponge *Rhodopsins*, the eumetazoans (*Nematostella* and *Trichoplax*) and sea-urchin genomes are provided in Additional file 4.

Phylogenetic analysis using Bayesian and ML methods was performed to resolve the relationships between human and sponge *Rhodopsin* GPCR family sequences (Figure 2). The results obtained from both tree building methods indicated that the largest differences between human and sponge GPCR repertoires was within the *Rhodopsin* family. Although a few of the sponge *Rhodopsins* were placed in the same branch containing human *Rhodopsins*, they lacked reliable confidence value support from both tree making methods (ML and Bayesian). Taken together, our results from the phylogenetic analysis suggest that the sponge *Rhodopsins* lacked unambiguous orthologous relationships to any of the known human subfamilies (see Figure 2). This is consistent with the BLAST results that could not classify sponge *Rhodopsin*s into subfamilies. Moreover, it must be mentioned here that a few of the sponge *Rhodopsins* had their top hits (see Additional file 3) as amine and opsin-like receptors in the blast search. However, these sequences failed to form a coherent group with the human *Rhodopsin* homologs. Instead, they clustered separately and are found scattered within the major node that grouped the sponge *Rhodopsins* (see Figure 2). This distinctive repertoire of sponge *Rhodopsin*s had relatively high similarity between them and form five observable clusters (Figure 2). Here, we putatively labelled these sponge specific clusters as *AqRho* A to E.

Since the phylogenetic distance between the GPCR dataset representing human and sponge was large, we wanted to investigate whether similar phylogenetic relationships existed between sponge and other closely related species. Therefore, we extended our study to three additional species having completely sequenced genomes. This included two non-bilaterian animals from the eumetazoan lineage, the placozoan *Trichoplax* and the cnidarian *Nematostella*, as well as the deuterostome bilaterian, sea urchin (Additional file 4). The phylogenetic trees indicated a similar topology wherein sponge *Rhodopsins* lack orthologous relationships to those found in *Nematostella*, *Trichoplax* and sea urchin (Additional file 4)*.*

### *Adhesion* receptor family

**Figure 3:**
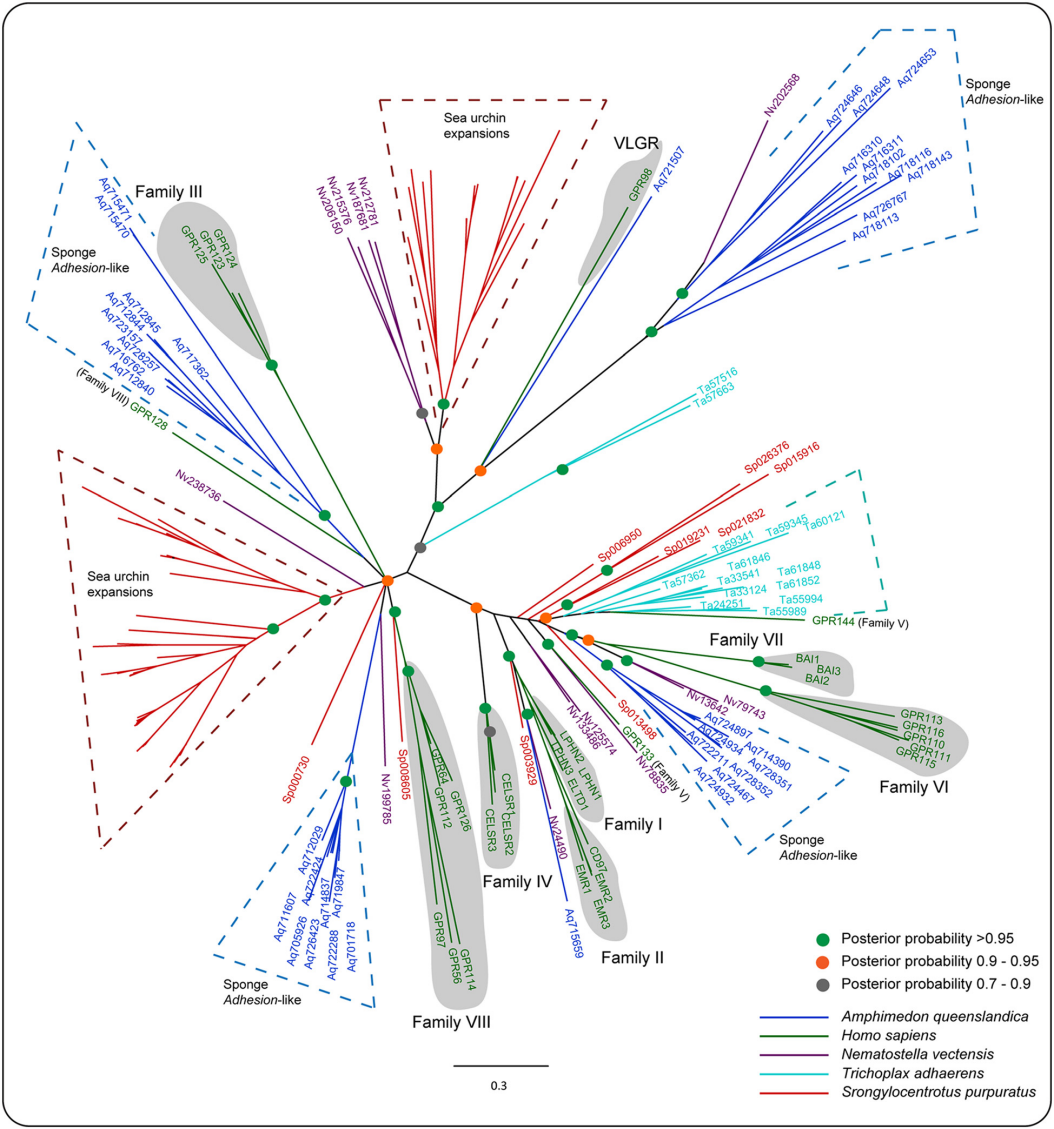
**Phylogenetic relationships between** ***Adhesion*** **family GPCRs in sponge and other genomes.** The color scheme for the branches is according to species used. The posterior probability values >0.95; 0.9 -0.95 and 0.7 to 0.9 are highlighted in filled green, orange and grey circles, respectively. Accession numbers for most of the sea urchin *Adhesions* were removed from the final representation for display reasons. Human *Adhesion* GPCR Groups I to VIII are heighted in grey.

The human genome contains 33 *Adhesion* receptors that phylogenetically cluster into eight main groups (I-VIII), with VLGR1 placed as an out-group. Earlier studies demonstrated that potential homologs of genes belonging to families I, III, IV, V, VIII and VLGR1 are present in most invertebrates, whereas families II, VI and VII are more likely to be vertebrate innovations [6-8,26]. To explore whether sponge *Adhesions* show homologous relationships to any known *Adhesion* GPCR groups, we included 33 human *Adhesions* and all identified sponge *Adhesions* for phylogenetic analysis. Furthermore, we included *Adhesions* from other metazoans to explore their relationship with sponge *Adhesions*. Phylogenetic analysis revealed that a few sponge *Adhesions* were placed basal to the node that contained human *Adhesions* belonging to family VIII (Figure 3). This tree topology was better supported when the analysis was restricted to only human and sponge *Adhesions* (Additional file 5). Also, the sponge *Adhesion* sequence Aq715659 clustered basal to the node containing human Group I and Group II *Adhesion* sequences (Figure 3). Additionally, two more sequences from sea urchin (Sp00392) and *Nematostella* (Nv24490) were placed in the same node containing human Group I and Group II *Adhesions*. This implies that these sequences are putative ancestral representatives of Groups I/II (Figure 3). A closer examination of the phylogenetic relationships showed that there were several *Adhesions* from sponge, *Trichoplax* and *Nematostella* placed in a major node that contained human *Adhesions* from groups VI and VII (Figure 3). The remaining sponge *Adhesions* are most likely sponge specific, since they clustered separately from any known *Adhesion* groups. This observation was consistent with other analysed metazoans, where most of the *Adhesions* from sea urchin and other genomes clustered separately from the human counterparts (see Figure 3).

Another noteworthy observation was that some of the sponge specific *Adhesions* have short N-termini and lack GPCR proteolytic site (GPS). However, these protein transcripts contained the core 7tm_2 domain region, characteristic to all *Adhesion* GPCRs. It is possible that these *Adhesions* were incompletely modelled at the N-termini due to sequencing errors. An alternative explanation is that these sponge *Adhesions* may truly lack a GPS site and the N-terminal domains, since the divergence is also reflected in the transmembrane helices that were utilized for phylogenetic tree making. These sequences clustered separately from rest of the sponge *Adhesion* GPCRs (see Figure 3). However, future experimental verification, as well as mining of *Adhesions* in other sponge genomes, is required to confirm these attributes of sponge *Adhesion*s. This might also clarify whether the short N-termini are more prevalent in ancient *Adhesion* GPCRs and the long N-termini was later gained due to addition and shuffling of key domains during the course of metazoan evolution.

**Figure 4:**
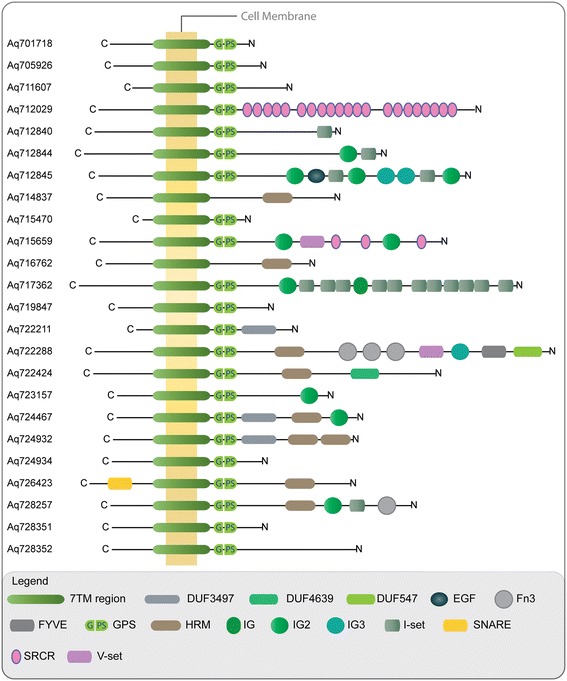
**N-terminal domain architecture of a selection of sponge** ***Adhesion*** **GPCRs.** The domains were identified by aligning sponge *Adhesions* to the latest version of Pfam library. Few *Adhesion* GPCR sequences that lack N-terminal domains are not shown. The domains shown in the figure include; 7TM: seven-transmembrane domain, DUF: Domain of unknown function, EGF: epidermal growth factor-like domain, fn3: fibronectin type III domain, GPS: GPCR proteolytic site domain, HRM: Hormone receptor domain, IG/IG_2/IG_3: immunoglobulin domains, I-set: Immunoglobulin I-set domain, SNARE: soluble N-ethylmaleimide-sensitive factor (NSF) attachment protein (SNAP) receptor domain, SRCR: Scavenger receptor cysteine-rich domain, V-set: Immunoglobulin V-set domain.

Although, a few sponge *Adhesions* lack a GPS site and N-terminal domains, the rest show diverse domain architecture similar to that observed in other metazoan *Adhesions*. The GPS domain, a common cleavage site for many members of this family is present in 28 out of 40 sponge *Adhesions*. Another interesting feature was the presence of a HRM domain in at least eight of the sponge *Adhesions* although hormones have not been reported in sponges (Figure 4). Intriguingly, we could also identify sponge *Adhesions* (Aq712029 and Aq715659) that harbour multiple repeats of the Scavenger receptor cysteine-rich protein (SRCR) domain (Figure 4). To the best of our knowledge identification of SRCR repeats is unique to *Adhesion* GPCRs and it is worth mentioning that SRCR repeats are often associated with immune system functions in vertebrates [29,30].

### *Glutamate* receptor family

**Figure 5:**
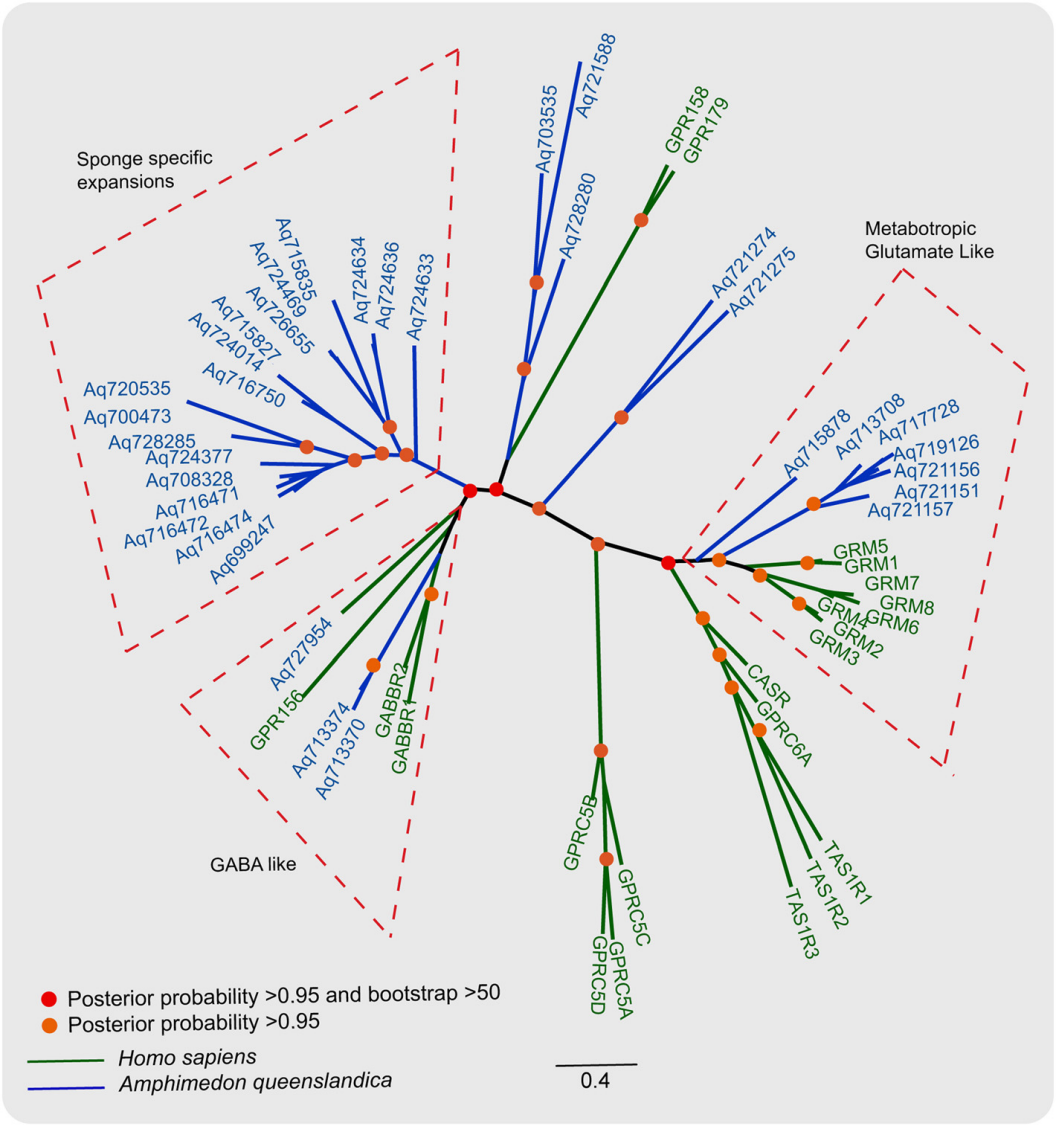
**Phylogenetic tree showing relationship between** ***Glutamate*** **family GPCRs in sponge and human.** The edges containing human *Glutamate* family GPCRs are highlighted in green, while the edges containing sponge *Glutamate* are highlighted in blue. Phylogenetic relationships between sponge *Glutamate*, the eumetazoans (*Nematostella* and *Trichoplax*) and sea-urchin genomes are provided in Additional file 6.

*Glutamate* receptors (GLRs) are crucial modulators of neurotransmission, and in humans there are 22 receptors consisting of eight metabotropic glutamate receptors (GRMs), two GABABRs, the calcium-sensing receptor (CASR), the sweet and umami taste receptors (TAS1R1–3), GPRC6A and seven orphan receptors [2,4]. Phylogenetic analysis of the *Glutamate* family members from human and sponge (Figure 5) revealed that sponge had seven GLRs homologous to human metabotropic *Glutamate* receptors (GRMs). In addition, phylogenetic analysis revealed that two sponge GLRs clustered with human GABAB receptors and another three were placed on the same node containing GPR158 and GPR179, but with a low posterior probability support (Figure 5). To test the robustness of these relationships, we included *Glutamate* GPCRs from *Nematostella*, *Trichoplax* and sea urchin. An overall unrooted tree obtained from a large dataset demonstrated that among the 33 indentified sponge GLRs, only seven are homologous to GRMs, while the rest were sponge specific receptors (Additional file 6). However, the sponge GLRs that had similarity to GABAB and two other orphans (GPR158 and GPR179) failed to give stable or consistent topology in a larger dataset and clustered separately from the known *Glutamate* receptors, suggesting they are divergent from other metazoan counterparts.

### *Frizzled* receptor family

The sponge proteome dataset contained three full length members of the *Frizzled* GPCR family. A comparative phylogenetic analysis with *Frizzled* receptors from sponge and other metazoan genomes was performed. For the phylogenetic tree construction, we also included the closely related smoothened GPCR family members from human and other metazoans. Phylogenetic relationships revealed that sponge *Frizzled* GPCRs are fast evolving or divergent from other metazoan counterparts (as indicated by Long Branch lengths) (see Additional file 7). Two sponge *Frizzled* receptors were placed basal to the node that contained human FZD9, FZD10 receptors. Also, one *Frizzled* receptor each from other analysed metazoans was placed in the same node with human FZD9, FZD10 receptors. Interestingly, one sponge *Frizzled*-like receptor was placed basal to the smoothened receptor cluster (Additional file 7). This finding was consistent with a recent study that identified a smoothened receptor in sponges [31]. It must be mentioned here that our initial screen for *Frizzled* GPCRs identified 9 *Frizzled-*like GPCR sequences, of which six were removed due to fragmentary models that contained less than 4 TM regions. Similarly, an earlier study identified eight *Frizzled*- like GPCRs in the sponge genome [14]. However, a few of these seem to be incompletely modeled and were not included in the final sponge GPCR dataset for better handling of the MSA (Multiple sequence alignment) data for subsequent phylogenetic studies.

### Other GPCR families

Our analysis revealed that the sponge proteome dataset also contains members of other GPCR families that do not belong to the GRAFS classification system. These included cAMP-like, intimal thickness-related receptor like (ITR-like), lung 7TM receptor-like and ocular albinism like (GPR143) receptors. Subsequent cross-genome phylogenetic analysis was performed on these GPCR families with the corresponding family members obtained from other species (Additional file 8). Protein sequences belonging to these GPCR families were obtained from *Nematostella*, *Trichoplax*, and sea urchin using Pfam HMM profile based searches. Corresponding sequences from human were obtained from the Swiss-Prot database. Overall phylogenetic tree topology indicated the presence of GPR143-like, lung 7TM-like and intimal thickness-related receptors in sponge. These sponge sequences clustered with their corresponding family sequences obtained from other species (Additional file 8). They formed separate clusters in the phylogenetic analysis with high confidence support. Phylogenetic analysis also revealed that the 14 cAMP-like receptors identified in the sponge genome form a separate cluster with high confidence support (Additional file 8). These 14 cAMP-like sequences contained the core region encoded by a Pfam domain (Dicty_CAR; PF05462) characteristic to the *Dictyostelium* cAMP GPCR family. Similarly, pairwise similarity search performed using these sequences as queries clearly demonstrated that cAMP family sequences are among the top hits. However, sponge cAMP-like receptors clustered separately from the *Dictyostelium* cAMP GPCR sequences, suggesting that they are quite divergent or fast evolving. It would thus be interesting to experimentally verify whether the cAMP-like receptors in the sponge genome have analogous roles to the previously known functions of *Dictyostelium* cAMP GPCR family.

## Discussion

The draft genome, as well as the transcriptome of *Amphimedon queenslandica* (sponge), revealed the genetic complexity of this primitive animal in detail and catalogued the presence of several crucial gene families, including GPCRs and other signaling system components [11,12]. However, a detailed curation of sponge GPCR families/subfamilies and phylogenetic comparisons with those versions found in eumetazoans and bilaterians needs to be performed to better understand the GPCR component in sponges from an evolutionary perspective. In this study, we curated GPCRs in the sponge genome and have phylogenetically compared the receptors to those found in other metazoans. Our HMM based search approach and phylogenetic analysis demonstrates that sponge contains four of the five main GRAFS families, namely, *Rhodopsin*, *Adhesion*, *Glutamate* and *Frizzled*. It is noteworthy that the sponge genome encodes one of the most ancient metazoan lineage specific expansions of the *Rhodopsin* family of GPCRs [11]. Moreover, our phylogenetic analysis with pre-bilaterian metazoans homologs clearly reveals that the *Rhodopsin* family has undergone significant diversifications in these pre-bilaterian metazoans. Possible explanations could be that they diversified due to the evolution of diverse morphological characteristics and adaptations of these species during the course of the early metazoan evolution [23,32,33]. This is also evident in other GPCR families, where phylogenetic analysis revealed that most members of the *Adhesion* and *Glutamate* families grouped into sponge-specific clusters. In summary, the study describes the sponge GPCR gene families in detail and our phylogenetic comparisons postulates a significantly diversified subset of GPCRs in sponge.

### Sponge *Rhodopsins*

Comparative phylogenetic analysis demonstrates that sponge *Rhodopsin* family GPCRs do not share orthologous relationship with those found in eumetazoans and other bilaterians (see Figure 2 and Additional file 4). In addition, sponge *Rhodopsin-*like GPCRs form five distinct clusters that are most likely sponge specific (Figure 2). Here, we putatively labelled these sponge specific clusters as *AqRho*-A to E. It must be mentioned here that several *Rhodopsin*s belonging to these sponge specific clusters are contained in the same contig region and located adjacent to each other. Several of the flanking sequences are found as many as a cluster of 2 to 8 sequences and share relatively high pairwise protein sequence identities ranging from 51% to 74%. This suggests that the expansions of sponge *Rhodopsins* are possibly driven by gene duplication events and it seems most likely true for other pre-bilaterian metazoans as well. However, to further examine whether these sequences can be classified into any of the known 13 *Rhodopsin* subfamilies, we performed a BLASTP search against the Swiss-Prot database. The results obtained from the BLAST search showed that a few sponge *Rhodopsin*-like GPCRs had their top hits as adrenergic, serotonin, dopamine, and opsin-like receptors (see Additional file 3). This is in line with the draft genome report of sponge (*Amphimedon*), which demonstrated the presence of serotonin and dopamine-like receptors [11]. This is also consistent with a recent study that identified adrenergic-like receptors in sponges [31]. Although the pairwise similarity search results suggest the presence of these putative receptors, our phylogenetic analysis was unable to reveal any clear orthologous relationships of the sponge *Rhodopsins* to the bilaterian counterparts. A possible explanation could be that sponge *Rhodopsins* have diverged considerably, possibly based on sponge specific physiology and behavior [19,20]. This hypothesis is plausible because species such as *Trichoplax* and *Nematostella*, belonging to the eumetazoan lineage and diverged from sponges later in the metazoan species tree, contain *Rhodopsin*-like GPCRs more similar to bilaterians than sponges. In fact, earlier studies provided evidence that eumetazoans do contain putative orthologues for some of the amine and peptide binding receptors [34-37]. The recent genome release of *Mnemiopsis leidyi* suggests that ctenophores are the sister group to the rest of the extant animals, including sponges, and that components of neuronal signaling were already present in an early metazoan ancestor [38]. Also, the same study proposed that components of neuronal signaling have undergone major loss and gain events in pre-bilaterian lineages [38]. Considering these observations, it is likely that *Rhodopsin* family GPCRs expanded independently in these species and may perform diverse functions based on the morphological characteristic of the organism [24,32,33,39]. Also, it is possible that these large expansions may have evolved to perform neuronal functions in ctenophores and cnidarians, and that this ability is secondarily lost in sponges and placozoans during the course of metazoan evolution. Nonetheless, at present it is evident that sponge *Rhodopsins* expanded due to gene duplication events and seems to have diverged considerably from those found in other pre-bilaterians and bilaterians. Further comparative genomics, as well as developmental/neurobiological studies would be essential to understand the roles of *Rhodopsin* family GPCRs that predated the divergence of Bilateria.

### Sponge *Adhesions*

The repertoire of *Adhesion* GPCRs (40) in the sponge genome is one of the first expansions within the *Adhesion* GPCR family at the roots of metazoan evolution. In comparison to the sponge, the closest unicellular metazoan relatives such as *Salpingoeca rosetta* and *Capsaspora owczarzaki* contained only a few genes (<10 genes) coding for *Adhesion* GPCRs. Furthermore, it must be highlighted here that our initial HMM search in the sponge genome identified a staggering 72 genes that encoded a 7tm_2 (*Adhesion*) domain. However, we removed 32 of those from our phylogenetic analysis, as they were lacking three or more helices. Therefore, it would be interesting to explore whether the subsequent improved versions of the sponge genome contain more full length *Adhesion* GPCRs. Collectively, this may suggest that the expansions of *Adhesion* GPCRs at the early origins of metazoans, relative to unicellular relatives, were possibly driven by the evolution of multicellularity in early metazoans since cell-cell adhesion is one of the major factors involved in driving multicellularity [33,40-42].

Another noteworthy observation is that a few of the sponge *Adhesion* GPCRs are found to be phylogenetically similar to vertebrate *Adhesion* GPCRs belonging to group I/II (see Figure 3). This suggests that some of the *Adhesion* GPCR subfamilies may have diverged early in metazoan evolution and would have later evolved or co-opted for more specialised functions, which we observe in bilaterians. Of note, previous studies suggested that group I and group II *Adhesion* GPCRs have potential roles in neurogenesis and migration [43-45]. Also, in contrast to *Rhodopsins*, which mostly bind hormones and neurotransmitters, the identified ligands of *Adhesion* GPCRs are mostly single-pass membrane proteins [45-48]. For instance, LPHN1, a Group I *Adhesion*, interacts with teneurin-2, FLRT1/3 and neurexin I-alpha & beta, which are all single-pass membrane proteins with a variety of N-terminal domains [46-48]. A BLASTP search using these single-pass membrane proteins (Teneurin-2, FLRT1/3 and neurexin I-alpha & beta) as queries against the sponge proteome obtained few reliable hits (PAC:15710607, PAC:15719742, PAC:15719354). Interestingly, these hits contained a single TM helix at the C-terminal end, N-terminal functional domains like laminin G-like, laminin EGF-like (similar to neurexin I-alpha & beta), and cadherins that are widely known to influence cell-cell adhesion, cell differentiation and migration [49-51]. This implies that some of the sponge *Adhesions* might interact with single pass membrane proteins to aid cell-cell adhesion in sponges. Thus, it would be interesting to explore the functional roles of ancestral Group I/II like *Adhesion* GPCRs in sponge and eumetazoans.

Although the sponge genome has a few *Adhesion* GPCRs that are somewhat similar to vertebrate *Adhesions* and placed basal to families I, II and VIII, most of them formed a distinct cluster and are likely to be sponge specific. Intriguingly, at least eight *Adhesion* receptors contain a hormone receptor domain (HRM) in their N-termini, a common characteristic of *Secretin* GPCRs (Figure 4). The absence of *Secretin* GPCRs in the sponge and the early presence of an HRM domain in *Adhesions* supports our previous hypothesis that the *Secretin* family descended from *Adhesion* GPCRs in an event somewhere during the split of cnidarians [26]. To the best of our knowledge, sponge *Adhesion*-like receptors are one of the most ancient GPCRs containing a hormone-binding domain. The HRM domain is essential for *Secretin* GPCR activity and is conserved in all the *Secretin* receptors. This suggests that HRM domain containing *Adhesion* GPCRs, found before the divergence of Bilateria, may have a possible role analogous to that observed in *Secretin* family of GPCRs [12]. However, this hypothesis needs further experimental verification since the presence of the HRM domain is surprising due to the lack of hormones in the sponge genome. Another distinctive feature of the sponge *Adhesion* GPCRs is the absence of a GPS domain in as many as 12 of the *Adhesion*s. It is important to note that the intra-molecular processing at a GPS site in the GPCR autoproteolysis-inducing domain (GAIN), proximal to the first transmembrane helix is attributed to several factors including signalling, membrane trafficking, as well as for the formation of heterodimeric GPCR complexes [45,52]. However, the absence of a GPS domain in some sponge *Adhesions* might imply that the evolutionary requirement for the conservation of the cleavage site is not very stringent or that the GPS site is more essential to those *Adhesions* with long N-termini. Moreover, it is possible that the missing GPS domains are an outcome of incomplete or missing regions in the current sponge genome draft assembly. The subsequent draft assemblies may help provide a complete picture of *Adhesion* GPCRs in sponges.

### Sponge *Glutamate* receptor family

Cross-genome phylogenetic analysis between the sponge and human (Figure 5) suggests that the sponge has homologous representatives for metabotropic *Glutamate* and GABA-like receptors with a similar N-terminal domain architecture, commonly observed in bilaterian counterparts. Also, we attempted to search for components necessary for a GABA shunt, a process by which GABA is produced in animal cells [53]. By homology search methods we found strong evidence for the presence of GABA-T (GABA α-oxoglutarate transaminase), which catalyses the synthesis of L-glutamate and glutamate decarboxylase, which catalyzes the synthesis of GABA. This is in line with an earlier study that identified glutamate decarboxylase in sponges [31]. However, these results are not surprising because GABA and metabotropic glutamate receptor-like GPCRs were identified previously in sponges, as well as in the amoeba *Dictyostelium discoideum* that evolved well before the divergence of metazoans [54,55].

It is interesting to note that previous studies provided potential insights into the role of glutamate receptors and neurotransmitter glutamate in non-neuronal cells [56]. For example, Elliot and Leys [57] showed that sponges, which lack neurons, use metabotropic glutamate and GABA receptor signaling for organized contractions of the sponge canal system. These roles of *Glutamate* receptors are rather distinctive from the commonly known functions of *Glutamate* GPCRs in a synaptic environment. Interestingly, there is growing evidence that challenges theories proposing the early origins of synapses and first components of a protosynapse somewhere close to the origins of cnidarians. A current hypothesis suggests that nerve cell components evolved at the very origins of metazoans and have undergone major loss and gain events in pre-bilaterian lineages [38,58]. Also, a few studies have suggested that glutamate is found in non-excitable cells, providing insights for glutamate to function beyond its general role acting as a neurotransmitter (see review in [59]). Therefore, the presence of *Glutamate* GPCRs in almost all pre-bilaterians including sponges shows the dynamic nature of *Glutamate* GPCRs, which seem to be functional both in synaptic rich and synaptic free environments that prevailed before the divergence of Bilateria.

## Conclusions

We present the first overall analysis of the GPCR repertoire in the sponge genome and have compared this to the eumetazoans and bilaterian versions. In summary, the sponge GPCR repertoire contains four of the five GPCR GRAFS families, as well as other GPCR gene families including cAMP-like receptors, intimal thickness-related receptor like (ITR-like), lung 7TM and GPR143 (ocular albinism) receptors. On the other hand, sponge lacks many of the classical mammalian-like sensory receptors including the olfactory receptors that are widely found in several bilaterians [60,61]. Moreover, our phylogenetic comparison reveals that the sponge *Rhodopsin* family does not share orthologous relationships with eumetazoan and bilaterian counterparts. This might imply that subfamily level diversifications of *Rhodopsins*, common in several bilaterians, likely became more pronounced later in the metazoan evolution, as indicated by the presence of some of the subfamilies in *Nematostella* and *Trichoplax* [34,37,62]. Nonetheless, the sponge encodes one of the first expansions of *Rhodopsin* and *Adhesion* family GPCRs early in metazoan evolution. Also, unexpectedly, sponge *Adhesions* encodes hormone binding domains, although hormone-like peptides are yet to be found in sponges. Similarly, the long N-termini of a few sponge *Adhesions* contain diverse domain architectures commonly observed in other metazoans. In conclusion, our analysis provides a wider framework for understanding the sponge GPCRs and to relate them to versions found in other pre-bilaterians and bilaterians. Furthermore, our data set comparisons provide a platform to perform more comparative genomic studies for understanding GPCR biology and signal transduction at the early origins of multicellularity.

## Methods

### Proteome datasets

The complete proteome dataset of *Amphimedon queenslandica* (sponge) was obtained from the Ensembl Metazoa database (http://metazoa.ensembl.org/info/data/ftp/index.html) [63]. Complete proteomes for *Trichoplax adhaerens* and *Nematostella vectensis* were downloaded from US Department of Energy Joint Genome Institute (http://genome.jgi-psf.org/) [64]. The sea urchin *Strongylocentrotus purpuratus* proteome was obtained from National Center for Biotechnology Information (NCBI) genomes (ftp://ftp.ncbi.nlm.nih.gov/genomes/).

### Identification and classification of sponge GPCRs

The complete sponge proteome sequences were searched against the Hidden Markov Model (HMM) profiles corresponding to each Pfam protein family contained in the Pfam database (Version 26). The complete search against the Pfam database was performed using Pfam_scan.pl script available at the Pfam homepage [65]. The pfam_scan.pl script aligns sequences with HMM profiles of Pfam domains using the HMMER3 software package [66] and obtains only the best aligned Pfam domain contained in each sequence. The same procedure was also employed to identify GPCRs in *Trichoplax, Nematostella,* and sea urchin. The obtained GPCR datasets from these genomes were utilised to perform comparative phylogenetic analysis with the sponge GPCRs. For the search against the complete Pfam database, the standard settings were utilized as provided in the Pfam_scan.pl. To ensure high specificity, we considered only the Pfam-A families matches, as each Pfam-A HMM profiles were built using a manually curated seed alignments and gathering thresholds (a cut-off threshold value determined for the sequences to be included in the full alignment) [67]. We retrieved sequences containing seven transmembrane domains/families belonging to the GPCR_A Pfam clan (CL0192). This dataset included sequences containing Pfam domains 7TM_1/*Rhodopsin* (PF00001), 7TM_2/*Adhesion* (PF00002), 7TM_3/*Glutamate* (PF00003), *Frizzled* (PF01534), as well as the domains corresponding to other GPCR families including, Dicty_CAR (PF05462), GpcrRhopsn4 (PF10192), Lung_7-TM_R (PF06814) and Ocular_alb (PF02101). All retrieved sequences were subsequently analysed for the number of helices using HMM based topology predictors Phobius [68]and HMMTOP [69] with default settings. In order to better handle the multiple sequence alignment and subsequent phylogenetic analysis, we discarded incomplete or fragmentary sequences (sequences containing less than five trans-membrane regions) from our final dataset.

Furthermore to categorize the sponge *Rhodopsin* in to subfamilies and to examine the similarity of Sponge *Rhodopsin* to those found in other species, we performed a BLASTP search against the complete GPCR repertoires from human, sea urchin, *Trichoplax* and *Nematostella* (Additional file 2). Furthermore, sponge *Rhodopsin* like GPCRs were subjected to a BLASTP search against manually annotated and reviewed *Rhodopsin* (7tm_1) GPCRs obtained from the Swiss-Prot database (see Additional file 3). We utilized standard default settings for the BLASTP searches, with a word size of three and BLOSUM62 scoring matrices. To categorize the sequences into subfamilies, the classification criteria were that they must have at least four of the five best hits from the same subfamily in the BLASTP search.

### Sequence alignment and phylogenetic analysis

Multiple sequence alignments analyzed in this study were generated using MAFFT [70] using the E-INS_I version (optimal for sequences with conserved motifs and carrying multiple domains) with default parameters. Thereafter alignments were manually inspected and trimmed to 7TM regions using Jalview software. The phylogenetic analysis was performed using the Bayesian approach implemented in MrBayes version 3.2 [71]. Markov Chain Monte Carlo (MCMC) was used to approximate the posterior probability of the trees. Analysis was run using the ‘*gamma’* distribution model for the variation of evolutionary rates across sites with ‘*mixed’* option to estimate the best amino acid substitution model. Each analysis was set to run for 10,000,000 generations and every 100th tree was sampled. A stop rule (standard deviation of split frequencies < 0.01) was applied in order to decide when to stop the MCMC run. All Bayesian analyses conducted in this study included two independent MCMC runs, where each MCMC run uses four parallel chains composed of three heated and one cold chain. The first 25% of the sampling were discarded as the ‘*burnin’* period. A consensus tree was built from the remaining 75% trees with ‘*sumt*’ command using 50% majority rule.

In order to verify the topology of the Bayesian phylogenetic trees supported by the posterior probability, we performed bootstrap analysis using the Maximum Likelihood method implemented in RAxML software [72]. Maximum Likelihood trees were computed for the trees showing human and sponge GPCR relationships and bootstrap values were indicated as percentage for the nodes (see Figures 2 and 5 and Additional file 5). We utilised four categories of rate variation across the amino acid sites and 500 bootstrap replicates were generated for the estimation of node support. Evolutionary model and parameters appropriate for phylogeny was determined using ProtTest [73] based on the Akaike Information Criterion (minAIC). Whelan and Goldman [74] (WAG) amino acid substitution matrix was obtained as the best model to determine the evolution for *Adhesion* data set while Jones–Thornton–Taylor (JTT) was obtained as best substitution model for *Rhodopsin* and *Glutamate* data sets. The phylogenetic trees were visualized and drawn using FigTree 1.3.1 (http://tree.bio.ed.ac.uk/software/figtree/).

## Availability of supporting data

The raw data supporting the results (raw alignments and tree files) of this article are available in the Dryad digital repository at doi:10.5061/dryad.43t7r (http://dx.doi.org/10.5061/dryad.43t7r) [75].

## Additional files

Additional file 1:
**Identified GPCRs in sponge (** ***Amphimedon queenslandica*** **) in FASTA format.**
Additional file 2:
**Blast hits of sponge** ***Glutamate*** **,** ***Rhodopsin*** **and** ***Adhesion*** **GPCR families against GPCR repertoires from eumetazoan and bilaterian GPCRs.**
Additional file 3:
**Blast hits of sponge** ***Rhodopsins*** **against reviewed list of GPCRs obtained from the Swiss-Prot database.**
Additional file 4:
**Phylogenetic relationship between** ***Rhodopsin*** **GPCRs in sponge and other metazoan genomes.**
Additional file 5:
**Phylogenetic relationships between** ***Adhesion*** **GPCRs in sponge and human.**
Additional file 6:
**Phylogenetic relationships between** ***Glutamate*** **GPCRs in sponge and other metazoan genomes.**
Additional file 7:
**Phylogenetic trees showing relationships between** ***Frizzled*** **GPCRs in sponge and other metazoan genomes.**
Additional file 8:
**Phylogenetic tree showing relationships between ‘other’ GPCR families (cAMP-like, intimal thickness-related receptor like (ITR-like), lung 7TM receptor-like and ocular albinism like GPCRs) in sponge and analyzed metazoan genomes.**

## Abbreviations

7TM: Seven transmembrane regions
cAMP receptor: Cyclic adenosine monophosphate receptor
CASR: Calcium-sensing receptor
EGF: Epidermal growth factor
FLRT1: Fibronectin leucine rich transmembrane protein 1
fn3: Fibronectin type III domain
FZD: Frizzled
GABAb: Gamma-amino butyric acid type B receptors
GAIN: G protein-coupled receptor autoproteolysis-inducing domain
GLR: *Glutamate* receptors
GPCR: G protein-coupled receptor
GPS: GPCR proteolytic site domain
GRMs: Metabotropic glutamate receptors
HMM: Hidden Markov Models
IG_2/IG_3: Immunoglobulin domains
I-set: Immunoglobulin I-set domain
L-DOPA: L-3,4-dihydroxyphenylalanine
LPHN1: Latrophilin-1
MCMC: Markov Chain Monte Carlo
Pfam: Protein families database
SRCR: Scavenger receptor cysteine-rich protein domain
TM: Trans-membrane
VLGR: Very large G-Protein-coupled receptor
V-set: Immunoglobulin V-set domain
