# Supplementary material for: The GPCR repertoire in the demosponge Amphimedon queenslandica: insights into the GPCR system at the early divergence of animals
Source: BMC Evol Biol. 2014 Dec 21;14:270. doi: 10.1186/s12862-014-0270-4 (PMC4302439; doi:10.1186/s12862-014-0270-4)
Supplement: Additional file 1: — Identified GPCRs in sponge ( Amphimedon queenslandica ) in FASTA format. [file 12862_2014_270_MOESM1_ESM.pdf]

Amphimedon Queenslandica GPCRs

Glutamate like GPCRs

>PAC:15699247 pep:novel scaffold:Aqu1:Contig2107:184:1032:1
gene:Aqu1.200719 transcript:PAC:15699247 description:""
IRIWLFSLGYNLCFGVILSKTWRVYYIFNNPKPNKKGVKDWVLLVIILIVVAIDLAIILV
GFIIPMSRLKSSEEEDTIHPQNINDEGKLQDHYILICNQSATVIWLALSFGYKGLLQVSA
IFMAFHTRRVKVKILNESKEIAAIIYINSIVLVLLAASEFTLATHHNAYAALFGLGLLTE
ATLFLGLIFIPKMVRLYLDPEGEKIFTRSDAPVTSTLQTIASTIDKK

>PAC:15700473 pep:novel scaffold:Aqu1:Contig4813:448:1516:-1
gene:Aqu1.201945 transcript:PAC:15700473 description:""
MHSFSLRVTKLSNVKINVVLLFGCTVLYSTGPLYTNSFSGFNETLQQSVLCNFRVWVFSI
GYMLSFTVIMAKAWRSVTVHPITASSNSQNVKDWMLYLIILILSVIDVFIIFVEAVNPQT
RINAKILYDGQIITENSTVVKEYYFECTRNKIIVATSLSYRSFLQLMCIFFVYHTRRVEV
SALNEAKQTSVIIYTNSIFMVLSGVIQFTLDHEINSVLIPVLLILQPTLFLAMIFIPLLL
LIYIGDRYFAKSENEGHSNNISSNDEENTQMNKKETTAKLQARIKELENEVTEMSMTMRR

>PAC:15703535 pep:novel scaffold:Aqu1:Contig9669:19:3751:-1
gene:Aqu1.205007 transcript:PAC:15703535 description:""
MFAYIFGHYLSTLQISYSVTSVLLDNNRRYPYFHTTIPNDEYYSVVLSKLLDHFDWQKVA
IISTFDKQSALVLQSIFRALAHHNEQAHIFEPVKDLVSAELVLKSAKLVLSDFFVKSKSY
FVIKSKRIFTKSRNLFEVGLCINNYFYSLYYLKDGKECIIGYYDSTNSTHHLQECETEYC
CNVTWKDGEITRSSPIFNAIITFGCTLGYISCLLFLGNYYIGLTGKGKLGPICNVAMSSI
SLSFLLIFSTILIKNWRIFQIFHNPSQQDRKYLSNTVLYIWMVIICAPVIIILILIHLFK
PLKVYQVSFPDHNCPGYPDNIESSLIEDHCIEASSFGWGAAIPVYFVILLIMIIYLGSWN
CRITRHYKSEGELAILTATVSIMIYIFNYGISLIATYGYTEAGMIKFILRYSIGTLFVYF
SFILAALYGFKVVQLLRDKKAHSRPVKRSRSTLRKKFLELSDEIFTLKCKLEKMNTSAVA
LEQ

>PAC:15708328 pep:novel scaffold:Aqu1:Contig12544:3941:5081:1
gene:Aqu1.209800 transcript:PAC:15708328 description:""
IIKLTSYKLNYIVILGSGLLYTSVFFYSLSLQNPTIQTIFCNGVRDWHLLLIIFSIVAID
VVIILVGSTLPQSRLTSFQAVDGEHPQVVNEEGKVQRNYVYVCNKQIGTIIWLGVSFGYK
GILQVLAIFMAFHTRRVKVKLLNESMETATIIYINSIIIVSLIVTEFTLPTHHNIYSALF
GLGLLIEATLFVGLIFIPKMIRLYFDPQGDNIFPQNTTVAPATLTNNILDEKESSEVVLN
LKKRVKELERQLVELK

>PAC:15713370 pep:known
scaffold:Aqu1:Contig13185:55705:62564:1 gene:Aqu1.214842
transcript:PAC:15713370 description:""
MLYFSFFLSSLLLSSSIIQTDGNANKTPLRLQILVPAEKNPTYWDGGVIPAMLMALDDVN
NASHILSNYTLTANISDTKSYVISEPTAALTGRLYKIPQVSYASYSPVLSDQSMYPLFLR
TAPSDLLMNRPRFALMATYDWCYVATLHQTANLFSLAVQDFHESFKSTLGTGPTGTCSGR
ADNDNPVLFSFTGDPQIPLMQIKSKKIRIIYGFFYEDKAVLVMCYARKLGLTTNNNHLWIL
PGWFSESWMDSANTLDWSPYNCSCNSDEIMEAAKYSLLVDAFSQLTDATLVSDSGYCQYC
HSSSQTNASYFKEYYSRRDSYFNITNATPSYDIPIELSSNAMYDAVWATALSLNATETKL
QSMGRNLSEFEYSDSTFTFLRDTFSSNISKMVTENMKNLDFIGVSGRVTFDGNGTRLTYT
QISQYVPGTRMINGTERDVLNTVRLAIYNETETGLDITYVVEPKWRLGRPPTDRSRPVSI
LISPAVIYTMDTVLAIGIIIAVVLLVFQIVTIRKPLMANSAPYINIILIIGCIVMMGSSI
LLGIDSGTPQVTDGDRDKILDEIGPSAKNRYAIICMTRVWLLTIGFTLSFGSLFAKTWQV
YRVYTKADLGKQPFKMWNFFIIMAIQLTIDAIFLSIWIGVFRFSHRIYEEDNEAENAIRK
HEYCYCDNFSYLVGSLYVYKGLLVVFGLFLAYESRNVKYTFINDSRFVSIAMYIVVVLIA
ISAPLSLVLADQHFIIDPSYAIAVLLIFITCMSCLMILYIPKFYYLAKGADTMVPSSKNE

AFLPVDNLQLGHTDVNPEDVQSAEQKIKQLKEQVKQREIELLSLARRASDQDSGVLCAST
EDTGADGSNRGSESPATMQTLQDTVTTVSSEGNGGDTINNNDVEEEENEGEREGERHEKL
PQEMEEGRQEKDVKERGDDDEEDINVAPLTRKKTVTIADVEEIEL

>PAC:15713374 pep:known
scaffold:Aqu1:Contig13185:68034:70195:1 gene:Aqu1.214846
transcript:PAC:15713374 description:""
MATLTSRPLINLSGEFTYKPPLPGNYSTKRMQPSGCYNINAIEIRETDTEILMANSAPYI
NIILIIGCIVMMGSSILLGIDSGTPQVTDGNRDNILDEINDSAKNRYAIICMTRTWLLTI
GFTLSFGSLFAKTWQVYRVYLDERKKNKPLKMWNFLLIMVIQLIVDVIFLSIWTGLFRFR
HGIDTVKDNNNINYSKFEKCECNGFIYLIGTLCAYKGLLLVFGLFLAYESRNVKYTFIND
SRFVSIAMYIVVVLIAISAPLSLVLANQHFIDHAYVVAVLMITIASLSCLLVLYVPKFFY
LAKGLDAMVSNNKAEVFTPVDNLKLGYQEETDTTTCNTPNSDNQTTNVTSFSTSPGTTQP
NTPADIMTTGDSCGSSVPSSSEKEDFEFRQDERP

>PAC:15713708 pep:novel scaffold:Aqu1:Contig13204:34:819:1
gene:Aqu1.215180 transcript:PAC:15713708 description:""
MIFILIGITLCFVVTVIYLIKPSIVVCTLQRAGLWFCFSIILSALLVKLVRISRIFMQKT
VTKRPKFISPKYQILFTFILVGIQMILVLISLVASYPKVKKSIVNNEENQNDFPSLVIQC
NSPHTAMIAILMVYYSILLIASNALAILTIRFPQNFNESKYVAFSTFSLGLMWIAFIFTY
LNTADKFQTAVVSFTIQMSAMAVLLCLFAPRVFIVLFLSKHKVYDTDNRKNAMTGEDCKI
KPKVLISSSTQETVTTDFKNN

>PAC:15715827 pep:novel
scaffold:Aqu1:Contig13301:102911:114579:1 gene:Aqu1.217299
transcript:PAC:15715827 description:""
MNSCLLFLLAFIITSSAQPNCSHQPDELRFLFMASNGSSFNSFSSLLGVDIALERINSNS
SILPNFTLSYSNAADSQCLRTPSLSAFLSQRIAPCSNFLAVIGSGCSVATDPVAEIGPFW
GLSHISFASSSVHLRDRDRFKNYFQIYASENNYIPGIASIMTFYGWSRVVIITEKVTLFT
ETEETLFELLTTKSVNATRIDFESDDNPADIPNLFPENERIFFLNMWEPKARQVICEAYK
RGYTDRTKYGWILYGWYSNAWWNLRSKNTTVTCTNEQLYSVLNGALAVQQYPINTNVTKT
AIGNLVDAGIESGLIPAESMRQKSYSEFYELYDARLADNVSYPSHAYLESTYVAHMSYDA
TWTLALALDRTARRIAGGQDPVPECSGTLTPLDQWDNTISTTSCLIRYELGRTSFEGLTV
KTIYGKSAKTAEIVPSKLSEYTGNVSFDDDGIRHNSPTRIYQIRNNEQQLVARWFEDNFV
YLNNESTETVFPDGVPSDGTPIRIVSTFHLSLVVIFYTISFAGLVFCIICLVFTFYFRNK
KLIKLISPNLNYFIIAGTSMVFLSIFLRIFPFDTSDEERFKKTRLLLCYISLYLDFIGYT
IAFGTILSKMWRIYYIFHNPSPNKKILTDWHLIGLILLITAGMAAILIIGSVIPQTRLKL
SLESDRENSNKFDERGVPLDYYVYHCFTTASFVWLIFLYIYLGMLQIIALILAIQTRKVR
IKILNDSKEVATIIYITSIVLVELMYRLYKDPKGESCETSQTMSSIEFGRSGALTVVSDK
ERIDQLQNKVLELQSQLEGKRVLNAENGDRKGPVTFIETSMVASITETIETAVKSTFTA

>PAC:15715835 pep:novel scaffold:Aqu1:Contig13302:5423:12089:-
1 gene:Aqu1.217307 transcript:PAC:15715835 description:""
MNFFLFFSLLAFIITANAQLNCSHEPDELQFLFMASNGSSFNTYSSLVGVDMALERINSN
SSLLSNFTLSYSDAVDSQCLRTPSLSEFLSQITAPCSNFLAVIGSGCSVATDPVAEIGPF
WGLSQVSYSSSSVHLRDRNRFKNYFQIYVSESNYIPGIAAIMTHYEWSRVVIITEKVTLF
TETEESLIELLNTKSIVATKISFESDEDPANISDLFPESGRIFFLNMWEPKARQVICEAY
KRRLTDRRKYAWILYGWYSNQWWTIPSTETPNDPFACTSDQLYSVLNGALAVQQYPINTN
VTKRVIGDLSYNDFYALYNARLNSSNSSYRNFQPTYVAHMAYDATWSLALALDKTAKRIA
EGQGPGPDCNGTLTPLDQWSNTITTTSCVIRYELGRTSFEGLSGNVSFDEDGIRPNSRTR
IYQIRNNEQLLVASWFQDEFIYLNNQNTETVFPDGVPSDGTPIRIISTFHASLVVIFYTI
SFVGLVFCAICLIFTFYFRNKKLIKLISPNLNYFIIAGTSMVFLSIFLRTFPFDTSNEEE
FKSTRLVFCYISLYLDFIGYTIAFGTILSKMWRIYYIFHNPSPNKKERGVPLDYYVYHCF
TTASYVWLIFLYIYLGMLQIIAIILAMQTRKVRIKILNDSKEVATIIYITSIVLVELVIV
SFALSSFYNTHEVLYDIGLLIVAFAILLVIFAPKMYRLYKDPEGEKCDTTQITSTVEFGK
SGAFTVVSDKEKIDRLQNEVMELKSQLDGRRASSSTYAENGDRKLSVSFIETSMVSLPIV
ETNN

>PAC:15715878 pep:known
scaffold:Aqu1:Contig13303:122284:125316:1 gene:Aqu1.217350
transcript:PAC:15715878 description:""
MKVDSFIFFGLLFLFYQSPILLAESNTEYVNSGGDVYLAGLFPVHANPGSSVRCGKILDL
GVQRLEAMVHAVELVNNDTELLPGIQLGFSIKDTCINSNYALEQSLSYVTSDIHFDTGNG
TTLRGVSGVIGAASSSVSIAVASLFRLFDLPQISYASTARFLSDKTRFDYFLRTIPPDLF
QAQVMVDIVVNYNWSYVIAIGTDDTYGQEGIKAFTEELGNSVSSTNKTFSVATTIKLQVT
ATEEDYDAAVVRMNDEWIRNSTVVVLFGQLATAEGVLRAVSRYANHRLMNFIVSDAVGDQ
LPSQYRSMGHSMISVLPRYYESQSFNEYFTSLLPSNNPNPWFKEYWSALFNCSFAANSTQ
QCNPEKERSTLISRYKQNSKVPFVIDAVYAFAHALQNLMLNVCGEIKLCDRIISYRDHGV
IINGELLLQYIKNVSFHGVSSEITFDSSGDVHGWYWIKNLQRKPNTVNEFWFLPVGSWNG
GTLNLTDDIEWATDKVPVSICSTDCTSGQFPQRVEGQASACWICVECPRKNEVSTGKKCT
VCPKGFSPNPDKDNCDVNPLVYLRWGHFFSIIILLLTLGGLIVTSITGIVFIVNYNHSTV
KASSRELSTVLLIGLILCYLLPFFSIGEPIAVTCGIRRFGFGFSFSLCYAALLVKVNRIH
RIFNRQRTSGQRPPLISPHSQLLFTGLLVLIQVILSASWLIVQIPGRKFVYYENTTEVTC
KATAYFGFVISLGYNFLLLIISTYYAFRTRKIPQNFNEARFINLTLYSIIIIWLAFVPAT
VGISQLGTVYETGAQVFAIVLSATATLCCIFFSKLYFMFSSKRKEGNEYQAGNNDRRFST
MQGENRINIFITPPTGQRSTGNASNLSPNLSPNLTPNISPNISPNVSPNVSPNVSPKASP
KKK

>PAC:15716471 pep:novel
scaffold:Aqu1:Contig13322:137731:141318:1 gene:Aqu1.217943
transcript:PAC:15716471 description:""
MKCFLLWSIFWLFFNTADAQRTTLYMGYITTITGSFQASGGRPAVDMALELINERDDILQ
NYTLTYTDVLDSGCNHTKGLDVLIELMNRDITYISLLGCGCSTSTISVAEISHYWNIPQL
AYAAGANVLNDKTRFKYFFRTLVSFRYVSQSLGQLMREFGWRQMSVITQDEPLFSRVTDE
LASIIFKDEGWILDRYDVPSGHNPLPFFDRSETQRFRIIHINSYPDIAYPVLCETSGEYL
SNYTDLLTNEPMYENLTALAISGVAFDGVWTIAIALDIASKKILSRNESGCENVPGDIVP
LEQFNYTNMKLGCILRQSFSEVNFLGVTGQIKFNRYGSRNDNVILYQQYRVINGEVGRYF
IGYVTFETPWKEGFFEFATGESMGTLWSDGVLPYDGFPIRVWLFSLGYNLCFGVILSKTW
RVYYIFNNPKPKKKGVKDWVLLFIILLIIAIDIIIILVGSTVPQSRLTSFEVVDSGNPQE
INEDEKFQNNFILVCNTRVGTLIWLGISFGYKGILQVLAIFMAFHTRHVKIKILNESKET
AAFIYINSIILVLLIVTEFTLSIYHNTYAALFGLGLLIEASLFLGLIFIPKASALNTF

>PAC:15716472 pep:novel
scaffold:Aqu1:Contig13322:146333:149576:1 gene:Aqu1.217944
transcript:PAC:15716472 description:""
MCLNTVVSQRIPLNFGYITTLTGSFIGDGGIPVVDMALRLINERNDVLQNYTLNYTDVLD
SACDRTMSLNQFFELINRNTTYISLLGCGCSPATIPVAELSHYWNIPQLSVVSAANSLDN
RIKFRNFYRTSVPFHDVGESLGQLMREFGWRQMSIITQDESLFSLVTENISNIFNNEGWT
LDRYDSETGSFRIFHINAYQDMAYQLLCEAYHRGMTGSDYLWIIPMWYNDNWWMSSSPTS
STNPSCTDAIMMEVITGTIGWIPRKYLTLNESITTFSGLTPNAYLGNYTALLQESRYRNI
TKFGFSGVVFDGVWAIAIGLDIASKKISSGNDSGCENVPGDLVPLEQFNYANTKLGCIFK
KSFNEIDFVGVTGQVKFNEFGSRNDEFIQYQQYRLSNSRLTKVHFGSVYVEGQRSSFVFS
SGESNSTLWKDGIPPYDGIPERGLDQNSIALIVIYDILAGFGIIFAAVCFIFNVVFRNKK
YAMMYKTTEAVFCNVRVWLFSLGYCLCFGVILSKTWRVYHIFNNPKPTKKGVKDWFLFLI
VSFIIAIDVFIILIGTIVPQSRLTSFEVVDGENPQIINDDGKLQNNFVVVCNTRTETLIW
EGLSFGYKGILQVLAIFMAFHTRGVKVRILNESKETAAIIYINSIVLLLLTVTEFTLATR
HNTYSALFGLGLLIEASLFLGLIFIPKASAVALK

>PAC:15716474 pep:known
scaffold:Aqu1:Contig13322:158027:161584:1 gene:Aqu1.217946
transcript:PAC:15716474 description:""
MLFNKRRAKIETKMQHIAFIFQCWMSSFIITVTSTKSPLHFGYITTLTGSFIASGGIPAV
DLALKLINERKDILQNYTLSYTDILDSKCNRTAGLDKFFELVNRDVTYVSLLGCGCSIST
IPVAEISHYWNIPQLSYATGADVLTDRNRFRNFFRTLVSFHYVGISLAHLMREFGWRQMS

IITQDESLFTGHVASNICDCFDLSEAQRFRIIHINTYPDIAYQLLCEVFLKAYYRGMTSP
DYLWIIPMWYNDDWWMPSSLSSYKNSSCTDAIMIKAVSGSIGFILDVFLTLQNKSLVTFS
GLTPQMYFSTYTALLKDPKYENLTELGLSGVAFDGVWAIAIGLDIASKKISSGSDSGCEN
VPGDIVPLEQFNYTNTKLGCILRQSFSEINFLGITGQIQFNENGSRNENIVFCQQYRTTT
DSSMARVTFGDVTIKPKGSIFVLKKEESNSTLWQRHGSLQPPYDGFPESNIHQNNMVLII
VYDIAAVCGLVFVVICFIFNIAFRSKKVVKMTSPNLNYIIILGSAMLYTCIFFYSHSSTT
KSMQSIFCNIREWMVSLGYSLCFGVILSKTWRVYYIFNNPKPNKKGIKDWVLFLIVFVVV
AIDFVIILVGSAIPQSKLASFKEVDHEHLQQANEKNRIQYNFILTCSQKTGTVVWLAFSF
GYKTMLQLLAIFMAFHTRRVKVRILNESKEIAAIIYINSTILVLLTVTEFTLANHHNTFA
ALFGLGLLTDASLFLGLIFIPKVNHAVLQIFKMIRLYRDPQGEKIFSDAENTCNTGASHA
VTNKVNTIQMVKEDGARTGNTNRSCSIRLYLLDK

>PAC:15716750 pep:known scaffold:Aqu1:Contig13334:8139:11806:-1 gene:Aqu1.218222 transcript:PAC:15716750 description:""
MLIPQQSIDKKAKSYGEILFDCFIEMANKSDVVSENGPVSFTSDGIRTVKRLRVLQYRNG
TLQEIAAIIVNQDNSLQFNYIENESDATVWPLGVPYDGVATNVTVSVSISITVIYSLLAG
CGIGFTVVCLIFNIFFRKNKLIRLSSPKLNYFIIAGAIIMYMSVIVYTLPSESELGNTVL
CNLKPRLFSLGYSLCFGTILAKMWRVYYIFTNPNMKKKSGIKDWHLSLFVLVVMIIDLLL
ITIPTAFEDARNTAKLIERKENPSEEEGVSYRTDYYTYICESRGRDIWLGVLYGFKGVLQ
VAALFLAFGTRRVKVKGLNESKFVAAIIYITSVVLAIKIIAAITLNEYVNVFSFLFATCI
LITATTTLGLIFIPQMIGLYRDPTGANIFYRESAAPSSAAAVAATETFKTMSLTGEKEIE
ISNDPFLSDKEKIEMLTKKIKELEKNSNEVSTKE

>PAC:15717728 pep:known scaffold:Aqu1:Contig13360:98899:101753:-1 gene:Aqu1.219200 transcript:PAC:15717728 description:""
MICQLTLLFLLTSLPPKTLGIQYQYESQPVILKSDGDTRISGIQDRIGKDIVLGGLFTVY
YSSGGTGVGSCGDEIWEDGMEMLEAMLFAIDSVNSDPTLLPNLTLGYDIRDTCKRENVAM
DETIDIVLSSGDLELESCAVVNTSTSEQVPVSVIIGAFESFITTPMAGLLRLFQKSQISY
GSSSTALSNRELYSYFFRTFPPDDKQAQAMVDLIVHYGWDHISTINSNNLYGQRGIEEVK
KHAAASSICIDFDAIITDEFKIADYANLASELLNSSANVVVLFASVHHVRSLFTELDRIQ
TLNSNERRFVWIASDTWAELTDREFNNVTAGKFGFAAFSENLDSFDVYYSQLLPSTNKRN
PWFPPYYEQYHKCKIDGIPVCDNTSVTNTSEYKQFSVVPLVMDAVYAAAHAIDNFIQDNC
DQPVQWYPGNQSCRGYNQSLNGDVLFRYISKVNFTSPSNNMVNFDSLGNIAVAKYSIFNY
QLIKCTSNDCPPLSLVNIGTWDSSVSNNRLQLYPNVRSQFGITSSGEGEYMPVLTLKSQC
QVCSPGHIKRIVVSSCCGTCDPCLGQNYTNASLTNTSCQTCPSDMWGNNPLTGSTGCVDI
SHSYLKPTDAWGIVLIVLSAVGLIAVVFVTCAFIWFWNTPIVKSSGREQMILLLIGLTLC
FLVTIVFLLKPSPVSCGFQRVGLWFCFSLVLSALLVKLIRISRIFLHKSISNRPKYTGSI
HQILFTFLLVGVQMSFVLVSMIVVHPDTITSTKLDENNHNDYPTLILKCTSPHIALIILQ
MLYFSALLIASNALAVLTIRFPANFNESKYVSFSTFSLGLMWFLFILSYIATNDSTIQTA
VISSTIQLSALAVLFCLFGPRVVIMIFWPQQNVLETAMPTQPTLLNSFPKADHKDGGSIN
LAKRTV

>PAC:15719126 pep:known scaffold:Aqu1:Contig13402:72118:74790:1 gene:Aqu1.220598 transcript:PAC:15719126 description:""
MNWQLSPLLLLIFSFLSIKAFGRIQHQYQVPSDVLRSDGGTRISGIQDRIDKDIVLGGLF
TVYFSSGGSNVGSCGDVIWEDGMEMLEAMLYAIDSVNSDPTLLPNLTLGYDIRDTCKREN
VAMDETIDIILSSGDLELESCAVVDTSISDQVPVSVILGAFESFITIPMAGLLRLFKKPQ
ISYGSSSTALSNRELYSYFFRTFPPDDKQAKAMVDLIVHYGWDHISTINSNNLYGQRGIE
EVKKHAAANSICIDFDAIITDEFKIANYTNLANELLNSLANVVVLFASVHHVRSLLTELD
RIQTENNNERHFVWIASDTWAELTDEEFNNVTAGKFGFAAFSENLDSFDNYFSQLLPSTN
NRNPWFSAYYEQYYDCKLNGTSVCDNSSITDASDYKQSSVVPLVTDAVYAAAHAINNFIQ
DNCIQPVQWYPNNQSCYGYNQSLNRDALFDYISKVNFSSPSNNWVNFNSQGNIAAAKYSI
FNYQLINCTANDCPSFSLVNIGTWDSSVDNRLLLDPNATSQFGIASSPTGEYVPIMALKS
QCKVCGPGQIKRIVVSSCCGTCDPCLGQSYTNASLTNVTLCQTCPNDMWGNNPLTGSTGC
VDISHTYLKPTDAWGIVLIVICAVGLIAVVFVSCVFVRFWNTPIVKSSGREQMILLLIGL

ALCFLVTVAFLLKPSPIICGCQRVGLWFCFSLVLSALLVKLIRISRIGFHKSMSNRPKCI
GPIYQILFTFLLVSVQMSLVLVSMIIVHPDTTTSIKLDENNHNDNPTLILQCTSPHIALI
ILQMLYISALLIASNVLAVLTIRFPANFNESRYVSFSTFSLGIMWFLFLLSYIATDDSTI
QTAVISSTIQLSALAVLGCLFGPRVFIMIFWPKQNDQGTVAPDLPTAYEV

>PAC:15720535 pep:novel
scaffold:Aqu1:Contig13434:222716:228112:1 gene:Aqu1.222007
transcript:PAC:15720535 description:""
MFLAAVLLSSLSLALSLDSRPKDIYFSYITTATGPFLSSGALPIIDYALDEINKNEDILT
NYTLSYGEVMDSKCQWKRSVDVFMDQFLNKSKFTYLSLIGCGCNIATQPVADISHYWSIP
QIEFASSSSSLCHQTVCKSFFQSVGSFQFIASSLVSLAVEMEWRQFAVLTEDNVYFTVIT
QQLNELFTNNEINIDSSILLTNNNNNNYRNFFNWTNGAVQRLIFINTYSPLAYQILCESY
SNDNYPFIAAKGYKNKMLYPEYVWITYDNYHDDWWAVNTKCDPSEMKNMINGSLSIIPED
YYPLLNESYNQTISGLSPALFHSAYANISQQGYTHQPVTSLSGSAYDTLWLLALGLNEAA
LRTASGDDTGCTSVSGSLVPLERFSYQNKKMGCVLQESIAQVTFNGITGPFEFNEDRGRD
TNSIITFQYRINYNGELVKVPVIRTSHDDITNSSNINFIQSVYRIWGRKCHHIIIIIIVV
VVVGIPYDGYPTERIVVLAPALRILFSVLTFFGIIFAGGCLFINTCCRKEKVTKLSNVKI
NVVLLFGCTVLYSTGPLYTSSFSGFNETLQQSVLCNFRVWVFSIGYMLSFTVIMAKAWRS
VTVHPITASSTSQNVKDWMLYLIILILSIIDVFIIFVEAVNPQTRINAKILYDGQIITEN
STVVKEYYFECTRNKIIVATSLSYRSFLQLMCIFFVYHTRRVEVSALNEAKQTSVIIYTN
SIFMVLYGVIQFTLDHEINSVLIPVTLILQPTLFLAMIFIPLLLLIYIGDRYFAKSETEG
HSNNISSNDEENTQMNKKETTAKLQARIKELENEVTEMSMTMRRVSVLNPINFDSITEEQ
SYVEIIYNN

>PAC:15721151 pep:known
scaffold:Aqu1:Contig13446:222124:224847:1 gene:Aqu1.222623
transcript:PAC:15721151 description:""
MSLAVAALLFLVSFSASESKYQFRNHPETVLYSNNGTRVSGIQDHFQKDIVLGGLFTVYY
DLGGTGEGSCGNVIFEAGIEMLEAMLHAIDSINSDQNLLPNMTLGYDIRDTCKRENIALD
ETIDMLFSSTDIQLDSASCQNSNMTLQSPVSVIVGAYESFLSIPVASLLRLFKKPQISYG
SSSTALSNREFYSYFYRTFPPDDQQAQAMIDLILHFGWDHISTINSNNLYGQRGIEEVKK
HAAANGICIDFDGFITNEFKIPDYIALARRLLNSSSDVVILFASLHEAETFLKELSKLHS
SLKSNRHFLWIASDAWAELTDEDFNNITSGKFAFPVYSKIYGSFNSYFSQLSPSVNLRDP
WFSRYYEQYCKENNCIHNSSITDTPGFRQFSVVPLVTDAVYAAAHAINNFIQDNCPHPLH
WQPHNQSCIGYNKSLDSETLRQYINRVNFTSPTGNRVAFDEFGNVQAKYTILNYQLVTSL
SCQSLKCSQDFELREVGVWDGSALGDRLEIFSNFTKQFGINQTTGDVLFSLNSYCQSCSL
GYFKRVVTSSCCGTCDPCLGSNYTNTNSSTMCMTCPDNMWGNNPLTGNTHCIDIDESYLK
PSDAWGIVLIILAIIGLIAVVSVTSVFIWFWNTPIVKSSGREQMMLVLIGITLCLLSALL
FLIKPSPTVCGLQRISLWFSSSLILSALLVKLIRITRIFMQRKVTTRPMFIAPAYQILFT
FILVGFQMLLVIISLSVAPPNIKQTKQYNSENYNDYPTLTLQCTLPHTATIVLQMLYYSI
LLITSNVLAVFTIRFPANFNESKYVSFATFSLTFLWLVFIPSYIITANTATQGYVMSFMI
QMSSIVTLLCLFGPRSFIMIFLPKMNVNKLQPTPNIKGFDKGIALKANIDSPANTVTKGT
ILDSQKE

>PAC:15721156 pep:novel
scaffold:Aqu1:Contig13446:249055:251713:-1 gene:Aqu1.222628
transcript:PAC:15721156 description:""
MEQPFLTSRVLFLSILLSMSDCAIQYDHTSPPERVLYSRTSDRITGFKDTLDKDVILGGL
FPVHVSQSMGGQCSDRFRSSGFQRMEAMLFAIDSINSNSSLLPGMSLGYDIRDTCVSENI
ALDESIDFVLESDQLEVESCGGMSNGNASDRVKVSSVIGASSSSVSIPVASLLRLFRVPQ
ISYASSSSALNNRDRYEYFLRTVPPDNQQAMAMIDLIERANWSFVSTIYSDNLYGEPGIN
QFHELAHRRGICIDLNLPIKESFLDYEYQYVADRLANSTANVTVLFTSFEHADMLLRTMT
NSNRSINKAGKVWIASDSTASTVLAANYAQVASELWGVLPATNLHAPFDDYYRQLSTSSN
KRNPWTQDAFKQLYNCAHNDCNVSSDYSQNEKVPLVIDAVYSIAYALKEFISDKCSAPLV
WYANNQSCQSHNGRTAVLNGSVLLQHLMNVSFTSPTGNEVSFNENGTVKAIYNIAVLKVE
QETSSYVLENVGYWNENLPIANRLHFYATQNENELFDDFTSQCQKCPPGQFKRAVTSSCC
GTCDPCLGSNFTNTNSSTMCMTCPDNMWGNNPLTGNTHCIDIDESYLKPSDAWGIVLIIL

```
AIIGLIAVVFVTSAFIWFWNTPIVKSSGREQMILVLIGITLCFVSTLFFLLKPSPAITRI
FMQQSISSRPRFITPPYQIIFTFLLVACQMMLVFISLLVVNPGVKKELHHNKTNDNNFPT
LILQCTTPHIALVVLQMLYNTALLITSNALAVVTIRFPANFNESRYVAFSTFSLGLMWFL
FILSYVATNGSTIQTAVLSSTIQLSALSVLLCLFGPRVFIMIFWPSHNSSESGMKPAGTK
DLESLPQRVPKSQTGVTVE

>PAC:15721157 pep:known
scaffold:Aqu1:Contig13446:253981:257698:-1 gene:Aqu1.222629
transcript:PAC:15721157 description:""
MVLLSSLLLLHSLLLCISGANYGYRDASETVTLSGNRIRGARNRTDVDIVLGGLFPVHSV
DDGGARCGSIRLERGLERMEAMLYALDIINSNASLLHGLKLGYDIRDTCSSENIGLDESI
DLVVSGQQLDLESCPTSDDTEYESANDSSEAALPTSTVIGAASSGVSVPVATLLRLFRIP
QISYASSSARLDNRDRYGYFYRTISPDSLQAEAMIDLCMEFNWTYVSTVYTNDFYGEPGI
DQFRKRAGETGICIDVDEGINSDSDDDDFSRIASLLLASSTNVVVLFANQGNARSLFSSI
EAINTANGTNKRFLWIASDAWARSISIVSLFNESLAGLFGFAPYTDESQGFQDYYSQLTL
ESNQRNPFFPEFYQSYFNCTINKTCNNTKPVTSHPRYQQGTFIPLVIDAVYSIAFAIRDY
LNDNCKQPIQWNRRTLSCDGNSDRLNGSVLLRYLNNVSFISPTGRHITFNPETGNAAGGQ
YQIVNYQRFLNKSYGFVPVGIWNGDNPNKRLSINHSISQFGLELINGQEVLLSRPRQSQC
TVCTAGQYIVEVEGSCCGTCSNCTGQEYSNTSSSSSCSTCPDRYWGNNPIIGSTSCVALE
ESYLRYRDAWSIVLMIMAIIGLISVVFVSVALGLYWNTPIVKSSGREQMILLLVGIALTF
LTTFFFVSKPSTFVCFFQRSSLWFCISFILASLLVKLIRISRIFLRQGSAASRPKFTEPR
YQILFTLLIVSIQFIVVLISMIVVYPLASEEQVESNMAGGPPIIQVTCDSPHLAPLIILA
LYHTALIIFCNILAVVTIRFPENFNESKYVAFSTFSIGLIWLAFTQTYIATSNEDRTAVV
SFALNLSCFAVLLCMFGPRIVIMIFFPERNVTQFTTQPKSRGSTDINAQFDTGYITTLDP
SLSVPTPKGSPRPTRVTLNLSDTQQKSGNGSFDDNSTKL

>PAC:15721274 pep:novel
scaffold:Aqu1:Contig13448:89589:93983:-1 gene:Aqu1.222746
transcript:PAC:15721274 description:""
MSTFHHLRLFVLLISAVLRSESQKIYSGERVVRFLALVQWRDEFNRTHSGWDTGPDMLAA
GRVAVQEINNRTDILTNYTLEMIEGRHEACGLTEANLGIKELASKGIGRENGSVVAVLGL
FCSTSAQHIAPVAGRAGLIQLSAANSPLFSRDLEKFPHLWRILVSAKAYTDMMLSLMRHF
GWEKVGLFQDLNTLFHSGIAESFIDLINKTNDKYTITYHGGILHTREELIDTALENIKTT
GTRIVFLQTTGPQTAKFLCAAANKNMVYPNYLWIITDFFLTGLIGELKGLSNCNEELLRK
GINGSLIGFFDLQDTEAVFTNASVAALNFLLLRHLTTKMIYSKPVLWYLTGLSLLLLTLA
SNGRDLQSEEGKEEERPVIRFLALVQWRDEFNRTHSGWDTGPDMLAAGRVAVQEINNRTD
ILTNYTLEMIEGRHEACGLTEANLGIKELASKGIGRENGSVVAVLGLFCSTSAQHIAPVA
GKAGLIQLSAANSPLFSSDLHNFPHLWRILVSAKAYADMMLSLMKRFGWRKVGVVQDLGT
IFHSGIAESFIDLIQDNDEYTINYHGGIIHARDELINSALDNIKATRTRIVFLQTTGPQT
AKFLCAAANKNMFYRDYLWIITDYFLTGLIKELEGLSNCNEELLRKGINGSLIGFFDLQD
TDAVFTNASGFNYSEYEHKYEEELNETRKEYYSSDDDVGDLLYAGLLYDQVWALALAINN
AIELYNVSMEDYSYDQYEVTDKIEKSLGRVSFRGATGHIMFSEDREVSTPINIYQYDTVH
ITAYFDPPPDSPESEMVPLSLYCAISLYVATLVVIVIVTIILICLLYMRSKPQIKASSLL
FSLIMFIGCYLICATCIVRITYAAFPSSEIIFEILCNVQSVFFFNGFSLVFVTLFIRLLR
IKKIFGNKSLTKYLGCCWTNKAMTLVVIVITIIPNLMLVGWIGFDRLKRETVNVTVDVEP
LVYNEEYFMCTSQLMYLWYAMVFTYTAFILLLIVILAACTRKIKHRDFKDTKKVNAFIFI
FVLTFTITGICLWLFREINQINVGHTVIISGLLLLVLECQVMLFLPKIMFCNRRRHRSYS
VTENVMTLFSFDKHEKH

>PAC:15721275 pep:novel
scaffold:Aqu1:Contig13448:100190:102436:1 gene:Aqu1.222747
transcript:PAC:15721275 description:""
MIIIVILLLCLSTFTETNKVVNLLALVPWPDDRESAGWDTGKVLLPAGRVAVKHVNNHKY
ILPGYSLRLIEDGHEACGLTEASKTIINFVRFASSPPHNTVAAVLGLYCSTSSIVLAQLK
EPVVLKLSASNSPVFKTHSYPNFWRFLVSAGIYGDMMIGLMNKFDWKRIAIVQDLETTFH
SAIAEELAKKILATSGEKEVVYHCGMVHTVDQFANDCINGIIDRKARIIFVSATGPQTAK
ILCLAGQRDMSYPFFLWIIIDFLVSSIIDEAGHIEDCDLSSLHYILENSLVAYFSLENTN
```

DLDSSFNVSIDDYLTAYRNELEADNITNPLKHDELYAGLLYDQVWAIALALNSSISKLEG
MNISIDTIGFNQPHVTQIVEESLSRISFHGVTGHIRFTSDNEVAGSVHIYQVSNSLEGLL
CHYEFQPLTNNYSFVNCQLSFSEEFDDDIPIQLLLLEAPIVISMFTGTCILIIFITIILL
LMAKCKNEPEIKAFSFSMNILIFLSCYVLCGTQLLSTVMAGVNIQSELAYSVICNIQVIG
TYSSICLVFVTLFMQLFRINRIFHNKRLSHLSCLYNNRVLFIITLLLQILPVVLQIVFVA
VIPFKLQLSESYEVVGSKTTLIRQRCCEIDSSTKNQLIFRFIFTLYEFVFLVLNTILASK
TSTISHRQFRNAKTVNVFMASVIIVQATSLSALYVLKQGSSSAMRYAELIVYIDSYLVTL
LCQALLFMPKILSVISRKSKQSLQSKHV

>PAC:15721588 pep:novel
scaffold:Aqu1:Contig13453:253962:257791:1 gene:Aqu1.223060
transcript:PAC:15721588 description:""
MERWWCLPISLFALIVFVVKASSLSCNPDSQTTLSLVGFFPCLRDAPASQLSHCDLMILG
SVELAIEDINASPAGEYNCFRLNLTHKITNNASEAVDAVKEYGFLNNRFLAQPTHGILGP
YSNQVALDLSYIFGRLFNIIQVTYSVTDSRLKDKARYPLLYSITPTDAFLNRLRSEVLRH
FGWRKVAVFSSDSARDTKVAEELADVLYNSTNKMSIMLGSFGSTAVSLLKIAKSNDVRVF
LPIVSEDKARAIFCEAYQSGLTSPSHVWILPGMSRSNWWKLLDNQTSNCTDEDVQNLTLR
VRERLNLPPFDFKNGYPPSLLHSKMFSAYDAVKLLAITWNDTINHLHNANTSKGLNATDI
IRDPMKDPTLGHLLMEKLIDATHRYTGFTLLGEPMLSMTSTGRGMVTQMFGNRESIVAVY
RHTIHEGSHFEVCTNGTGCGLSWKGGYQPTDGTSNTEPDHVLFYVIFFVLYFFIFVGGIS
FSIITCICKTNKYLKARTPFFTAILNVCCTGLSVSGIFYLVSNYIGLVQLKHIEVVSPLC
AIGFVVMQLSYVTILATVLVKNWRIYRIFYNPHIKNRKNLSTKDLSLGISAIVVPFMITI
TIIYSIYPPKEVENCLEMNDCMCLNRLGMWNIFPFLYFTILCIGLVILGISNSQLQSFFR
VEGQLANVVSIMFGLVGASIFIIQLREITSRSVVPSFSKILMVHVSIFISGFVFLIFVNA
PKIRIRQLNTTRHSKSSTSNLVSEDYLEKCKEIVKLKEQILKLS

>PAC:15724014 pep:known scaffold:Aqu1:Contig13489:2642:7036:1
gene:Aqu1.225486 transcript:PAC:15724014 description:""
MPAQNDPKAYCGVTNVADKPKEKTKMETKSFQYFCFPLIMSLQLIFLCICALASISHGSF
PLVYPQPSAESSNATSLYLAVMMSFNGTFLSSGTIPGIQIALDLINEQEDLLPGYKLHYI
ALDSRCNHTIALDSLFKQLISDTQRVGVIGAGCSVATEPTAEISHYYNITQVSCASSSHN
LANRVKYKAYFQMLPSAINLVPAIGAICLRYRWRHIGIIAQDENLFTQETRERIVFLAMY
PGHALTVVCEAYKKNRVYPRYTFIIYGWYSPTWWTSSASSIDCSREEIESVLHYSIAVLQ
HQFGTDRTQSTISGISYDTYHGMYDKRLSSFGYRYSTHNIQCFDGVWAFALALNQTINDL
KENETLSRLAQESEGWEDTETEFRIENATYKNNVIQQMMFGHLERTNFLGITGNVSFTNI
GIRRVNRLRVFQYRYTGNNTFGTVEFASVPALGNYTTLEYYNGESDSTVWPSQVPYDGVA
EIEIVSVPIPISVVFSLLAGAGMIFGVICLVFVIVYKKNKLVRLTSPKLNVLIIIGSLIM
YFSVILYTLPVYDSFGRTLLCNLEPRVFSLGYTLSFSTILAKMWRVYYIFTNPKPNRKAI
KDWHLSLFVLAAVSVDLLLLGLLAGYKGGRPEAYLVRNNENPSDERGTLGYITDYYVYTC
DSHEHSIFLGIIFGFKAILQVAALFLAFGIRKVKVRGLNDSKFVGLMVYISSIILTIQII
VSVTLNHYQFAFSILFGLSIIINTTVILGLLFFPQMVGVYKDPEGKEVFVGEQMKVKVKT
VLTSNEGAVSNIVSSPFMDDKQKIEALVQKINEMQENDVDMKDTAT

>PAC:15724377 pep:novel
scaffold:Aqu1:Contig13492:99010:102107:-1 gene:Aqu1.225849
transcript:PAC:15724377 description:""
MKLLCLLFLVSLSLSTSVSVFKKNSSLQFGYITSLTGSFIASGSIPVVDLALKLINERND
LLENYTLGYTDFLDSKCSYTSSLDQFFKLVNRNATILALLGCGCSTATIPVAEISHYWNI
PQVAFASSSLALFDRHRYRNFFRTIIAQRFIGTALAQLMVEFGWKQMGVITQDEALFQNT
RGLVLNGYVIKTGGSLDPFFARVSKFFISIMNDAMNFRINKINAYPSFSYQVLCKAYYRG
MYGSKYVWILPMWYAPDWWKTVSSGNCSCTDDIMLQMLNGIMGVVPEGFFPLENESITSR
IFADQYHFLLQEPKYENLTELGIAGLAFDAVWAIAVGLDIASKKILFGNETGCEKLSGEI
APLEYFNYTNKKLGCILKQSFSEVNFLGITVSQTIIGTTVNIMTFGRVTVESQRSGSPPY
DGYPVDRLHSNHIVLVVIYSLTAGCGIVFAVLCLTFNIVYSNKKVLMANNAKLNYLIILG
SAVLYISVFFFSYTPYNPKTQEVFCNSRSRLFSLGYNLCFGVILSRTWRIYYIFSNIKAK
KKVGIKNWMLLLIIIGIVLIDLIIIFTGRIKHAIVRDQEHPLVVDDNGRIVHYFVVICNS
SIISELFSFGYKGILQLCAIFMAFHMRNVNVKALNESKEIAAIIYINSIVLVMLGVTEFV

LDRSHNAYAALFGLGLLIDATLFLGFTFIPKIFNVYFNPHGGKLFTNTKIGKSMQSKVAP
ERIQDEDHCKIKNTSCSIGTLQ

>PAC:15724469 pep:known
scaffold:Aqu1:Contig13492:452411:460890:1 gene:Aqu1.225941
transcript:PAC:15724469 description:""
MVSATALLLYLSSLLLNAASQRLDVYIVYSNSGTYNSTPALLGATDAIAAFSNELLPQYS
LNITPLDDKCNASRGLQETLQYVSRPSPFVSIIGGGCPSTTSPLLEIAEYLKIPLISYNP
GLMRYSQNYVQVFPSRVSLVSSLAAVISEYGWSQISVITEVDDNFLKVEYALKLRLKSEN
VKVLDTVHMRPQTNITADLLFRTEARVFFLNTYQNTARRVLCTAHRNNTYYPGYAWLIYD
WYNEDWYLTDDSSGCMQREINEALDRAITIRQHKEIRPSEMDGYAFAYDAMVSLMRGLNR
TQSMIDEGINHNTSCPGGLPSSINETSNISNCVIRSNIIESAFQGKSLQTSSLYYMALWQ
HQIGDVSFTSDGIRKTIITYVSQYRESKLIRAEYTLKSNIEEFPISVSAQLVGFYMIPAN
NKHCRLVRIILIRFKLNYLFDDLVNHCCISSKLAFKGYRVLYNGGQLQVEPFATVNVSES
LFNYFSNESNSTVWPDGVPPDGTPIYEFSSFSIILVIVMYLWSSVGIIFTVICLIFNIIF
RNRKIIRLTSPNLNYFIIAGAIILYSLPFVTLQPSNAETVSLFVCIANIWMLSLGITSIF
APMLAKMFRIYRIFRNPRANKKVLTDWHLFFFVFILEGILIFILFLGTAIPFTRPEVSYS
EDPESPPIINEDGIKVIYQGVNCYHAASLVWIFVIYIYILILEAIAVVLAAKTRGVKVEA
VNDSKEVVAIVYIVTACSVSVIIIVTVLSEFTNISEGLYLTAALIGTTSTVTLMFLPKMW
SLYKDPTGENVFEPTTIKSSSGIQSQIHRQDSEKIVSLQREVGTLKKILRENSVRLMSVP
EAVNEIDGDESNNKNGGLRGLTNSCSQLPEVFGDPLTPGDEEKTL

>PAC:15724633 pep:known
scaffold:Aqu1:Contig13495:206785:217392:1 gene:Aqu1.226105
transcript:PAC:15724633 description:""
MIPLLLSLLCISSLQLPCVKSEDGNITFIFMLSRPSRQRDFLLEENTAGVQPAVDMALDY
INDRESLLSDYSLSYGEAAVNSQCDSRVSLRQFFDEIRSSPIKYFLLGAGCSLATENVGE
IANFWEIITMSYASQSSVLNQRSLYRNVFRTVPSSLGIADIVVALFAEYNWNKIVTVTQQ
ESEFEAIASAIYGSSMMAEQLYFQSDEDVANTVSFGTSSNTRIYLLNMYPPHARRVLCAA
YRNDPNIKYPRYVWLVYGWYPANWWTESVSGYDPMVTCTDEQLQSLLERSLILSNYPLTY
ETDSTPTDVGLTRSQFFAMYRNLTASYNYSESYAAPFAYDALWAVALAINSSIPSLSYNL
SQFDHKSIGRSITSDIANTLYNISFTGVTGTVSFNSNGTRRESEPRVSQYRLTNGELSPV
LIARINVSLNASGGLAYTWVYLNNENSGSVWSDGVPSDGTPEISIVGILVYVYVPVYILG
VSTIITGIIFAIVNFIFRSRKIIRLTSPNLNYLIVLGCILMSASGLIYSFPTTNYTAVYV
LCYIRAYTQLVGYSLCFGTICAKMWRVYFIFHNPTPKKRKAIKDWHLFAVVLVLCIIDLV
LMSTALGADRQYVPILVDDKERSNTDTNEYGIVYEYKNRVCDSPRSIIWIAIVFLKMFLV
QSAAVFFAIKTRRVKIKVLNDAKWISIIIYVTSFVLAVTLSGALALANFLNADAAVFSTG
LIVVSFLVLCILFIPKWYSLYKDPQGEKIFEKGSQPSFMSAGNNLTAVDHENMVHHIEQL
EKRLGKYEAVTPYQTRKRVLSSGSALDTNNPRLAALKIGNNSREGRSLSYSPGLDVTASL
PVSRLGLARSPAPPSPNNINNSGDSGVNDSSKENSEDKPRPAGNGNMFTLREEAEESNES
NKDLKELNDYKTESQRVLTFEDELHESPF

>PAC:15724634 pep:novel
scaffold:Aqu1:Contig13495:217764:223283:-1 gene:Aqu1.226106
transcript:PAC:15724634 description:""
MKASLRSVYLSLLLFLFTQDGVKAVCPPNTPSPPVPLYFGFMTAVDPQSDYVLAGIIPAV
DLALELINRNTSILPGYELSYNGTIYDSKCDHRQGLNEFFEMISSRTQYVAAVAAGCSAA
TLPVAEISHYWSLPIVSYSSAAAQLSDRTTFPSFFRTYPSDGNFVPAFVGLVRGLGWKRV
FIFTEESPLFTGNERSLFEELTAAGIYSQSQTFQSNDTAAGVIRDVINTNGHNGTHIFFI
NAYANSAIAILCQFYHLNKRYPNYAFITFGWYQTRWWDIDANPSINVPCTTQNLQTQLDR
VLILQQLPLALNGSAPTISGLTADQFDARYRSRLNDTEYINQLPPSVYNPIPDALTAFDA
LWSLALGLDRVQRAVCANETLGCNGGPLTELEDFMYYNDQVECMINKSMNMTGFEGVSGY
VEFNANGTRVQEAFTIFQYQENTVRVLIGTLSLADNNTFRYRNDTAVWPNGIPPDGTEIK
IINTIHVSLNVIFYTAAILGAVFAIGCLAFNVYFRNKKIIKLTSPNLNYTIGAGSLVMYS
SVIFLLLPGLDTYSLHLRCIVGYWLAMIGYIIAFSAVLAKMWRVYYIFHNPTASKRTLKD
WHLLVAVGVITSIVCLILIIGESIPQTRHEPTLVVDRETESTTNEFDVVVNYCTYSCYRS
TTSVAFQALIMAMLFLLQAIGIFLAFQTRKVKVKVLNDAKQVTAIIYVTSVCVVLIIMTT

FALGSFLNTSSAVFSLSIAAASTAFLILIFIPKMYNLYKDPDGENIFTGTHSSAAVPGIA
AGAASILTGVQPGNSFLLRKESIGINMALATLGDDPKATIAVLEAKVLELEEELRNSKMN
PGNPTKRGSLFPGFMSKNGLGNNRSSRDSTTEGSRASVTFEQPKDNLNAANSMTSPPSSV
IRESSEEIEEGSDDRDNTNVTSNGMNGNGVSNSGDCVKNNDGGKNEEEGTAKTSRTETNL

>PAC:15724636 pep:novel
scaffold:Aqu1:Contig13495:224535:230973:-1 gene:Aqu1.226108
transcript:PAC:15724636 description:""
MDFKLFLSTFLLFSGVAWAACPGDLPNGTLVPLYFGFMTAVDPHSDYVLAGIIPAVDLAL
ELINNNTSILSGYELSYNDTIYDAQVKTKALICSHRQGRDQFFSMISKRPPDFIALLGAG
CSAATLPVAELAHYWNFPIVSYSSAAPMLSDVSLYPSFFRTYPSDGNFVPAFVGLVRGLG
WKRVFILTEESPLFVQNERPLVEALSEAGIYGKSHSFLSGPDVDVAEVVRDVIYKNEHNN
THIFFLNMYSGKARAAFCAFYRLNMFYPNYAFIIPGWYQTGWWNNDAVNVSCSTRDLQTQ
LDRVLILQQLPVALNRSAPTISGLTADEFDTRYRSRLNDREYINRLPGPPSVYYPIPDAL
TAFDALWSLALGLDRVQRAVCANETLGCNGGPLTELEDFMYYNDQVECMVNKSLSMTGFE
GVSVNGIPPDGTEIKIINTIHVSLNVIFYTAAILGAVFAIGCLAFNVYFRNKKIIKLTSP
NLNYTIGAGSLVMYSSVIFLLLPGLDTYSLHLRCIVGYWLAMIGYVIAFSAVLAKMWRVY
YIFHNPTPSKRTLKDRHLLSVMGILTLIVIMILSIGEAVPDTRHEPTLVVDGEKGSTKNE
QDVIVNYCTYTCFSSHDAISYQALIMAYLFLLQVIGIFLAFQTRKVKVKILNEAKQVTAI
IYVTSVCVVLIIMTTFALGAFINVHATLFCFSIAAASTAFLVLIFIPKMHSLYKDPEGKK
VFRSSGLKAVPSLVPGGGGGGITTASGTVDSLRKDSLGINIALATLSNDPKATISILNAR
IVELEEELRQSRAENGGEKRGSLLSGFLSPKALPPVNENNIRRTEDSIEQLELSKKNSLS
PPPPPAPPSSRASNGNAAASFTEDSIQ

>PAC:15726655 pep:novel
scaffold:Aqu1:Contig13512:382660:391196:1 gene:Aqu1.228127
transcript:PAC:15726655 description:""
MNTCLLFLLAFIITNDAHNCSRQPDELQFLFMASNGSLYNSFGSLLGVDIALEKINSNSS
LLANFTLRYSNAADSQCRRTPSISAFLSQRIAPCSNFLAVIGSGCSVATDPVAEIGPFWG
LSHVSFSSSSVHLRDRDRFRNYFQIYASENNYIPAIASLITYYGWSRVVIITERVSLFTE
TEETLFKLLAAKSVAATRIDFESDEDPADIPNLFPESGRIFFLNMWEPKARQVICEAYKK
GYTDRRRYAWILYGWYNNEWWNIPSHSTPNNTFTCTSDQLYSVLNGALTIQQYPININVT
KTAIGNLSYNEFYELYNSRLANHSSYPEYRYILSSYVAHMAYDATWTLALALDRTAKRIA
GGQGPGPECSGTLTPLDEWNNTITATSCLIRYELGRTSFEGLTGNVSFDDDGIRCKSPAR
IYQIKNNEQLLVARWFQDEFIYLNNQSTSTLFPDGVPPDGTPIRIVSTFHLSLVVIFYAI
SFAGLVFCVVCLIFTFYFRNKKLIKLISPNLNYFIIAGTSMVFLSIFVRIFPFDTSNEKT
FKKTRLLFCYISLYLDFIGYTIAFGTILSKMWRIYYIFYNPSPNKKHSFPQSMLNKLTST
PYELTSTASKCMAYIINSAEQLSTNIVDEPILTDWHLIGLILLITAGMVAILIIGSAVPQ
TRLKLSLESDREHSNKFDERGVPLDYYVYHCFTTASFVWLIFPYIYLGMLQIIALILAIQ
TRKVCIKILNDSKEVAVIIYITSIVLVELVIVSFALSNFNNIHEVLYDAGLLIVAFTILL
VIFAPKMYHLYKDPKGENCDTSQSMSSIEFGRSGAFTVVSDKERIDQLQKKVLELQSQLD
GKTVLNGERKSVTFIETSMVASFTEANIDFSKES

>PAC:15727954 pep:novel
scaffold:Aqu1:Contig13519:918308:921002:1 gene:Aqu1.229426
transcript:PAC:15727954 description:""
MSPGLYLLTVVIIGPVLASASYNNVSKDQVIIAGLFAYNLSEVAPDRLHMDRTANGSVVE
LAVNMALSEYNESPFSQLRLTLWPYSTECDPARAVWSLTDALRRTDRSEPLVILGPPCED
STNELVRLTSSSTNILTVSYASTTPQLNNATIFRNFFRTVPSFTNLNDALINLLGYFSWE
NVCLILESHPYYNTAAEALSAMNLTIVESIPPALLQYITTNSYCHIFLVYSQPEYLPNIL
CQAYNNRLIGQYYQWIFVGNTEISDLTAEVDSINCSSQELLTAAQSSFVIGFDDDANNDD
PLVGISRSKFNNSLLNVSGVSATSNDLSVAASAYDAMWAIAISLNNSVNRLKERNQTIND
YLPLANKDVSATLFNEFQSVNFRGVSTMVNFTTMTHNTLKPIKISQVHQNSLVAIGLYKT
DTKLNMDYFGHQVQWIGDGPPRDSPVFINESVPLWAQIIMFTVSGIGALVLSFFLVINLC
FREKKVIKASSPHINTLILVGCLLGILSVSVYTLSSIESIVTDIRSIFCNSTLWLVNIMF
TLSFGALLAKTWRVGAVFRNPWSKRRIYKDYVLFLIVLVLLAVDVLILTVWMLTSPLYIS
VRMIQSPSAITELSFCSLSGEGVFFGILLMVYKSFLLLLGCFLATQTRGIKATLFNDSRF

IAIAIYIVFLIVIIGLPMAIFFLLGRLTIITAAEDYTLQAKLAMTMYYNVTT

>PAC:15728280 pep:novel scaffold:Aqu1:Contig13521:272963:278358:1 gene:Aqu1.229752 transcript:PAC:15728280 description:""
MLHLFYSLFISSLFLSSSSGQGVYGDHFNSSSSGDTPIESGSGSDSPATTTMPPTISTAI
PPPTPPPWFPQDCPCPYGKHLVYLVQPSPCYYEHERNSSSVLSLTSPRACDIHTYTGMIA
ADYNFVYNSQPGAPGLTLLNTFCFEPRSYSVITWYPNGKEDLPTMVRNLYYHYKAGNPLH
GVIAPHDQKLSERINYVVGKNIKRLVMSYSTPGLLQNRTVYPYQYTTSPSEIFLNKVRLQ
VLYDFNWRRVAIVSTDDAFSSEAAEQLFSISRDNTSMDINFPGSFLRDPTAIFEQLKEDD
YRIFVGFFPLKESRYIICKAMRMGLTTSQHVWILPGIYDYMWWHDCTCDNNDKFCSCNDC
TEEEMKSAIQGMLFIDASNLQITNVTETQDQIHWFHELFNFTRDFFIFPSIVNITEEDRQ
YLIHSRGTNAYDAIGLFYSTWHAAAQRLLSSTNDVTAICDSMSKPIRDSNDQLDLLSKTM
VDILETHVFCGVSGYYNYKGKNNNDEFGQAKVTQFQDDHEYTIGIYRNYEFSPDEVYCPR
NKLVELNESRITFSPSSLFFSNLTNFNFRQKNFGSSGPYRWKNKNGSFFYNCYTAPTDGA
CHDESCLCFNQIEESGISLSLFIMLIGIITSVLLILFNFHNRNNKHIKASSPKFTLFIII
GALVMYVSILFLALSFSGNFIIKSESSRKIALPVFCELDKWLSSLGMIITLSAMLVRNWR
LHKIFYNPKIANRSYLSDKILIPMMLLLIAPVCLLLIIWSSTDAYKYKEMYEAFNCTDIT
VCFLMVASQSEMKQLGQIVDDSITTELVTYTCSSSPQWRGLLVMYNLLLSCGLIFFSYQN
AKIRTHFSQEGEFAARAVFVVFLFVIPAFAIDLLLILTSTNSLGLFWIRLLLAASFPSVV
LGGLFVPKIIFMMRDKRELEKLKQRQTLSFSYEGKIKEIDHLKSVLSKK

>PAC:15728285 pep:novel scaffold:Aqu1:Contig13521:309083:312389:-1 gene:Aqu1.229757 transcript:PAC:15728285 description:""
MFLSVLLLFMVTGEVVGPRKLYILFVVSRTGSFVSSGVIPMVDYAIQEINNRSSILSNYT
ISYEEVLDSKCHGRTATNLFMDKFFDNPKLTYIVLVGCGCSVATIPVAQLSHYWNVPHIL
PTIDGIPYTLTVFAKRLGWRQLAVITQQESLFIGMTHKLNLLFQESGLILDDSITIETGK
PLPKERLFKWAYKRKMFSPKYVWIFYDSPKIDWFDPNITRSCKPPEIDRILNGSLLLLPE
AYSPSTNSSYISSSGMSPSRFTKLHRYLLRQPQYQGLPLVGIEGSGYDAIWAMSLGLEEA
SRRIAVGNDSGCKLLQGELVPLEEFTYDNPKMGCVLQKSIASVKFNGITGPFEFNKNRTR
IGNTIIIYQFSVNENGIRTLRKPMVRVVNRDGIRNSILFFIEPLSTLWGKIPYDGYAEKK
IREYNSAFVTVFTLLASAGVMFAGVCLAFTIIFRKKKIVKLSSPNLNYLLILGSVILYSH
MYLIVYYFTGYDEIAKQSILCNLSVWSLSFGYSLCFSVILAKSGRVYYIFANPSSKKKSI
KDWMLFIIVALLTGIDVLLLIIETSTRSTRVKTKIVPDTQQFEVAALVSDKDGTLKNYYF
FLCDHSRIFLAVKNGYRVLLQLVGIFFAFHTRKIKIKGLKESTQIGYIIYFNTLFLIILV
LSDYVINLNTDKSSSDTVARMRARVKELEEKVDKLRMNVRKFSLPATTESFGIEEEKEHY
L

Rhodopsin like GPCRs

>PAC:15699092 pep:novel scaffold:Aqu1:Contig1667:21:747:-1 gene:Aqu1.200564 transcript:PAC:15699092 description:""
MDDINNNFTLSEDVNGPLLAAVIGMEMLAGLITNSFVLILTACHLKNWKQPTTVFLSNML
ANNLVVILFTMPLSIITTASGEWIFGSTVSQKESACYFAACIFIFSILTATESLVLLSFD
RFFFIVKALQYKKYMTVNRAFIIVAVSWLLAAFLSMLPFFGFGAFEFAFSYGMCVPGWRG
QAGYAIFSFIVISIFIGSITVTSIWTMCFTRKYLKNAATNISTAASPGNPYAAQERRVIG
LF

>PAC:15699550 pep:novel scaffold:Aqu1:Contig2858:61:666:1 gene:Aqu1.201022 transcript:PAC:15699550 description:""
MEDMDENFTLSGDINGPLLAAVISIEMIGGLIANSFVLILTICHIKTWKQPSTIFLTNML
ISNILIVLFVMPFAITTAASDEWLFGKTYKQKMKVCQFTAFMFWFCKIVITEGLVLLSFD
RFFYIVKSFEYERHMNQKISIIIVTLSWLLAALLTIPPLFGLGRFSFSSSNGICVPHWEG
ESGYVVYMLIVFIIFIISLHN

>PAC:15700596 pep:novel scaffold:Aqu1:Contig5099:435:1619:-1
gene:Aqu1.202068 transcript:PAC:15700596 description:""
MILALIANGVVLVITIYQRKSWKQSSTIFFTSLILAHLVLTLYLPFSIAALAAGEWIIGD
TDEEKKATCDFNGFIILCCAYIIFMTLSLISTDRFLFIVKPHIHKRFMSPRVALVLVIIV
WIVNAAFYSSGFIDGSGVVYQYVVWTFCFTRKFINNQSMIVGESVYASKKKRLFGIFGSM
LLVYGICFIPGIFFGTLLAIIDFPVVLGITALIFFFLAVVLSPVVQAYFRPEINSVIMLK
RKPNPTTTSSSYISPSAALDEHCFHNSTLGIIVITVDHSYLSGLSPL

>PAC:15700739 pep:novel scaffold:Aqu1:Contig5403:607:1542:1
gene:Aqu1.202211 transcript:PAC:15700739 description:""
MDYNFTATGSINGPVLAAVFAVEAVVGFIANIIVLSITLYQRKSFKQPSTIFFTSLILSD
LLDVLVYLPMTTVSTGAEEWIFGSTFEQRRATCVFSGIIFWFILYVTTGVLAAISFDKCL
FITKPYFYKRFMKPWVALTITVALWIIIGLMFTLPLISFEDYYYYGIYFGPCYVGFLGKI
IYSIMFGLWLIFIVTIIVTSVWTFCFTRRFINNQSAIGGENAYNSRKMKLFGIFGSMLLA
YTLCYAPAVIIGFISFGFALPYEVFATTTVALHFAPIVSPIIQAYFRPEITKSLVSFKNA
LCEKLRCSHSN

>PAC:15701137 pep:novel scaffold:Aqu1:Contig6166:26:808:1
gene:Aqu1.202609 transcript:PAC:15701137 description:""
LAAEEWIFGSTDEEKRGTCYFGAFIFWWIVLVISLTLDAISLDRFLFIVKPHLHKRFMRP
WVALTLTIAIWILSAVLSIVHLFGFRLFIDICISSTANIGFVVYIILLSAIVLGIIFITS
LWTFCFTRRFFKDQSVIAGESVYASKKKRLFGIFGSMLLIYGICFVPGVLYFLLYLFIDV
PLWLNIAATICFHFITVTNPIMQSYFRPEIKSIYIVLY

>PAC:15701199 pep:novel scaffold:Aqu1:Contig6273:29:625:1
gene:Aqu1.202671 transcript:PAC:15701199 description:""
MVLVISITLATISFDRFLFIVKPHLHKWVMRPWVALTLTIAIWILSAVLSIVHLFGFHLY
IDICISSTADIGFVVYIILLSAVVLGIIFITSLWTFCFTRRFFKDQSVIAGESVYASKKK
RLFGIFGSMLLVYGICFVPAVLYFLLHLFIDVPLWLNLAATICFLFITVANPVMQSYFRP
EIKSVLFRCCPLKIYVCC

>PAC:15702185 pep:novel scaffold:Aqu1:Contig7896:57:1878:-1
gene:Aqu1.203657 transcript:PAC:15702185 description:""
MERNYTFTGDFSPEAVAGVLSIEMILALIANGVVLVITIYQRKSWKQSSTIFFTSLILAH
LALTLYLPFSIAALAAREWVVGDTDEEKQGTCGFTAFVILFGIYVMLMTLSLISIDRFLF
IVKPHLHKRFMSPRVALVLVIIVWSFDLLFFSTGFIDGSGVEFRYIDYLGVCYAFTTSPV
MAIFRFFFASLIFIIIIITSVWTFCFTRKFINNQSMIVGESVYASKKKRLFGIFGLMMLV
YGICVSPGIFFSTLLAIVDSPGVFTMTALMLFFLAVVLSPVIQAYFRPEINSVIVNIVCR
NMLKKKPNPAATSIVVLSPVVQAYFRPEINSVIVNIAYHNILKKKPNSATTSSSSSAAVD
LRVSSCT

>PAC:15702669 pep:novel scaffold:Aqu1:Contig8595:983:2091:1
gene:Aqu1.204141 transcript:PAC:15702669 description:""
MEGNFTFTGEFSGPAVAAVLTVEMILALIANGVVLSITLFYQRKSWKQSSTIFFTSLILA
HLVLNLLHLPFTITALAAGEWIFGNTDEEKSGFCLFIAYTVWYIIFVISITLAAISFDRF
LFIVKPHLHKRFMRPWVALTLTIAIWILSAVLCTGPFIDNGRYSYDGELGFCTLIGFDIA
SLSLILPTIFLVVGTIIVTSFWTFCFTRRFFKAQSVIAGESVYASKKKRLIGIFGSILLI
YGTPVLLSSHTSGLRSKIGLVLLVPL

>PAC:15702875 pep:novel scaffold:Aqu1:Contig8875:1923:2651:-1
gene:Aqu1.204347 transcript:PAC:15702875 description:""
MDFHTDGNDYFPDDFNGPLIAAALTVEFIGALVANGIVLIATLIQYKSFKVPSTMLFTSL
IIIHLIISVLFVLLWIISAASEEWIYGTTIEQKIAACTFHGCVLNYGIWIIFGTITAISV
DRCIFIVKPNFYKKFMKPKVTLTLIALIWVVALILDTASLYGFGEVAYGEYGACVPRFEG
EIIYVVITITLVIMKICIIIVTSIWTYCFTRKFIQEHSQLADDVYVSRNRRLIGIFCAML
IG

>PAC:15703005 pep:novel scaffold:Aqu1:Contig9021:79:1038:1
gene:Aqu1.204477 transcript:PAC:15703005 description:""
MDIMSFNSTDFVLTGDINSPTYAAVLGIEGVIGIIVNVAVLLMTLYQRKSWNQSSTIFFT
SLLLSNLIIALWYLISSIAVGAEEWIFGNTFEEKNATCMLVAYIIWYGIMDISATLAAIS
FDRFLFIVKPYLHKRFMRPRVALILITGVWLLCSLINTIPFYSTGGYRYFPHSGICSFLN
RTSAYFVVLIVVYAIIYSIIAFTSIWTFLFTRSFIKRQGRSVDRGVYQSKNKRLFGIFGS
MLLFYIIALLPSCIIIFSVFFDLPDGLYAFSICTYGLVAIINPLIQSYFRPEVKATLVLI
ASKIGLKSNKIGTSQATAG

>PAC:15704497 pep:novel scaffold:Aqu1:Contig10507:5186:5849:1
gene:Aqu1.205969 transcript:PAC:15704497 description:""
MILALIANGMVLSITLYQRKSWKQPSTIFFTSLILAHLVLNLLYLPFTIIALAAGEWIFG
STDEEKRGTCTFATFVFSYTIPVIFNTLAAISFDPFLFIVKPHLHKQFMRSWIALTLTID
IWMFSALLNSTPFFGLNDEQFLLCYQHPNSIINLIIFCFLSMCYKSFFKDQSVIAAESVY
ASKKKRLFGVFGSMLLIYGICVVPI

>PAC:15704762 pep:novel scaffold:Aqu1:Contig10729:4864:5633:-1
gene:Aqu1.206234 transcript:PAC:15704762 description:""
MSIPKEFLEAIINNILHFSHTGTLTLLYLPFNIIALAAGEWIFGSTDEEKRGFCSFTAYT
LWYSIPVIVITLAAISFDRFLFIVKPHLHKRFMRPWVALTLTIAIWILSAVTTFTPFIEG
SGAVFMYEGSHGTCTVVILELQFAIVSFVGSLLVVGIIIVTSVWTFCFARKFIHNQSEIS
GDNVYASKKKRLFGIFGAMLIVYGLCFTPGIINYAV

>PAC:15704842 pep:novel scaffold:Aqu1:Contig10790:381:1325:-1
gene:Aqu1.206314 transcript:PAC:15704842 description:""
MEGNFSYVGDFNAPAVAAVLTVEMILALIANGVVLSITLYQRKSWKQSSTIFFTSLILAH
LVLNLLYLPFTIIALAAGEWIFGSTDEEKRGFCSFNAYTLSYSGFVISMTLAAVSFDLFL
FIVKPHLHKRFMRPRWVALTLVIAIWILSAVLGAGPFLDIGHYSYDDKSGFCSLIGFDFA
AFVVILVIISLVVGTIFVTSLWTFCFTRSFFKAQSVIAGESVYASKKKRLIGIFGSMLLI
YGTCSLFVASGYLLQFFIFPPYEIYVTGYIVSYFVTVASPIIQSYFRPEIKSVLVSCCPL
LFTCVCCSFVHAVC

>PAC:15704843 pep:novel scaffold:Aqu1:Contig10790:4493:5437:-1
gene:Aqu1.206315 transcript:PAC:15704843 description:""
MEGNFTFTGDFNTPAVAAVFTVEMILALIANGVVLSITLYQRKSWKQSSTIFFTSLILAH
LVLNLLCLPFTIIALAAGEWIFGSTDEEKRWTCIFSAFILWFGAYVITITLAAISFDRFL
FIVKPHLHKRFMRPWVALTLTIAIWTLSVSVFGSLQFIGIGHYSYDDELGYCTLIDVDVA
SFVVILVIIFLVVVTIFITSLWTFCFTRSFFKDQSVIAGESVYASKKKRLFGIFGSMLLI
YGTSYLFLALGFILQALIFLPYELYITYYIVFCFVTIASPIIQSYFRPEIKSVLVSRCPL
LFTCVCCSCVHAVC

>PAC:15705622 pep:novel scaffold:Aqu1:Contig11404:790:1518:1
gene:Aqu1.207094 transcript:PAC:15705622 description:""
MEGNFTFTGDFNTLAVAAVLTVEMILALIANGVVLSITLYQRKSWKQSSTIFFTSLILAH
LVLNLLYLPFAIIALAAGEWIFGSTDEEKIGTCTSAAFVYSFTIPVIFMTLAAISFDRFL
FIVKPHLHKRFMRPWVALALTIVIWILSAVFSSAPLYGINEYVYEDQFLLCYQRPNGTMS
LIIIFVFLPCVTAVIIVVTSLWTFCFARSFFKDQSVVAGESVYEALGSTDPHHCYVAVCS
CI

>PAC:15706124 pep:novel scaffold:Aqu1:Contig11695:9419:10501:1
gene:Aqu1.207596 transcript:PAC:15706124 description:""
MSISSENNFTINDDVNGPVLAAVFATEMILALIANGAVLLITITQRNSWKQSSTIFFTSL
ILAHLVPNLLYLPFTIISLAAGEWIFGSTDEEKRGFCSFIAYTLWYSIPVITITLAAISF
DRFLFIVKPHLHKRFMRPWVALTLTLAIWILSAVTTFTPFIEGSGAVFMYEGSHGTCTVV
ILELHFAIFSFVGSLLVVGIITVTSVWTFCFTRKFIHNQSEIAGDHVYASKKKRLFGIFG
AMLIVYGLCFTPGIINYVVVRAIVVADEIYLAVVISFIFVTVLSPIVQSYFRPEIKTKIV
FISRKILQKPPQRDSIDYLGNSNNDSSGSKTEINQIRTSTQLKISSDHVLNTEHLSTTVV

>PAC:15706178 pep:novel scaffold:Aqu1:Contig11720:1222:2157:1 gene:Aqu1.207650 transcript:PAC:15706178 description:""
EGNFTFTGEFSGPGVAAVLIVEMILALIANGVVLSITLYQRKSWKQSSTIFFTSLILAHL
VLNLLYLPFAIIALAAGEWIFGSTDEEKRGTCTSAAFVYSFTIPVIFMTLAAISFDRFLF
IVKPHLHKRFMRPWVALTLTIAIWILSAVFGTLPFFDIGQYSYGDELGYCTLIGVDIAAF
VVILVIIFLVVGTIIVTSLWTFCFTRKFFKAQSVIAGESLYASKKKRLIGIFGSMLLIYG
TVYLIVALNFLLRIFIFLPYEFNVTGYIVFCFVTIASPIIQSYFRPEIKSVLVSRCPLLF
TCACCSCVHAV

>PAC:15706179 pep:novel scaffold:Aqu1:Contig11720:4382:5323:1 gene:Aqu1.207651 transcript:PAC:15706179 description:""
MAENFTFTGEFSSPAVAAVLTVVMILALIANGVVLSITLYQRKSWKQSSTIFFTSLILAH
LVLNLLYLPFTIIALAAGEWIFGSTDEEKTATCYFAAWMQWSGGSVLALTLAAISFDRFL
FIVKPHLHKRFMRPWVALTLTIAIWILSAVIGTLPFLDFGHYSYDDELGYCTLVGVDIPA
FVAILVIVFLVVGTIFVTSLWTFCFTRSYFKAQSVIAGESVYASKKKRLFGVFGSMLLIY
GTVYFSTALNILLQIFIFLPFEFNVTNYIVYFMVTIASAIIQSYFRPEIKSVLVSHCPLL
FTCVCCSCVHAVC

>PAC:15709198 pep:novel scaffold:Aqu1:Contig12731:6409:7389:-1 gene:Aqu1.210670 transcript:PAC:15709198 description:""
MDYNFTATDLDNFTATGSINGPVLAAVFAVEAVVGFIANIIVLSITLYQRKSFRQPSTIF
FTSLILSNLLDALVYLPMTTVATGAEEWIFGSTFEVRRATCVFSGIINWFIIFITVAVLA
AISFDKCLFIVKPHFYKRFMKPWVTLIITVALWLIVALIFILPSLGFGEYDYHFEYGPCY
ITFKKNIAFVLFYIATVLIFIIVIIVTSIWTFCFTRRFIRDQSAIAGENVYHLKKMKLFG
IFGSMLLAYGICYGVSFTIGIVSLGVTFPHEVYATGVFVFQFSIIATPLIQSYFRPDIVD
SLKSFKNFVYTQMKKLQYSLPTPSQT

>PAC:15709199 pep:novel scaffold:Aqu1:Contig12731:7869:8867:-1 gene:Aqu1.210671 transcript:PAC:15709199 description:""
MDYNFTATDLDNFTATGSINGPVLAAVFALEAVVGFIANIIVLSITLYQRKPFKQPSTIF
FTSLILSNLLDALVYLPMTTVATGAEEWIFGSTFEQRKATCLFSGVIFWFILYVTVAVLA
AISFDRCLFIVKPYFYKRFMKPWVALIIAVIPWMIIALIMMLPFVGFGEYGYQFDYGPCY
ITFENNMGFVIFRFATLFTFIIVIIVTSIWTFCFTRRFIRDQSAIAGESVYHVKKMKLFG
IFGSMLLAYVICYGVSITIGIISAAVTFPHEVYATGMFVFQFSIIATPLIQSYFRPDIVD
SLKSFKNFVYTQIKRQRYSPPSPIRLQTQHKF

>PAC:15709200 pep:novel scaffold:Aqu1:Contig12731:9312:10292:-1 gene:Aqu1.210672 transcript:PAC:15709200 description:""
MDYNFPAIDFDNFTATGSVNGPVLAAVFAVEAVVGFIANIIVLSITLYQRKSFRQPSTIF
FTSLILSNLLDALVYLPMTTVATGAEEWIFGSTFEQKKATCVFSGIIFWFILYVTAAMLA
AISFDKCLFIVKPYFYKRFMKPWVALIITVIPWMITTLIMILPLVGFGEYGYHFAYGPCY
ATFEKNMVFVIFRFATLFIFIIVIIVTSIWTFCFTRRFIRDQSAIAGENVYHLKKMKLFG
IFGSMLLAYGTCYGASFTIGIISLTVTFSEEVHATGVFILQLNIIATPLIQSYFRPDIVN
SLKSFKNSVYTQMKKLQYSPPTPSPT

>PAC:15709352 pep:novel scaffold:Aqu1:Contig12760:28957:29979:-1 gene:Aqu1.210824 transcript:PAC:15709352 description:""
MDNINDNFTLSDVINGPLLAAAFATEMIGGLITNSLVLILTASHLKTWKQPTTIFLSNML
LNNLVIGICIIPFAIITAAVGEWIFGRTEKEKETVCQVVGCIFTFTILTATESLVLLSFD
RFFFITKSFQYNKYMTVNKALVIVSLSWALAVFLSILPFLGFGVHEFLSGVGLCVAGWNG
QTAYAIVSFLVLCIFIGSIIVTSIWTLCFTRKHIKKTAIRTSIGAERSNHTQERKVIGVF
GMLIIVHLLCYAPIISFGLVESFINVLTPTAYAVAVFILFLLITLIPLVQSFFRSDIRTA
IVKGSMTVIAFTKRCRTTPHQISTVEMIRPSTPITSSSSI

>PAC:15710365 pep:novel

scaffold:Aqu1:Contig12925:43645:44373:-1 gene:Aqu1.211837
transcript:PAC:15710365 description:""
MHFHTDGNYSFPEDFNGPLIAAALTVEFIGALVANGIVLIATLIQYKSLKVLSTMLFTSL
IIIHLIISVLFILLWIISAASEEWIYGTTIEQKIAACTFHGCVLNYGIWIIFGTITAMSV
DHCIFIVKPNFYKRFMKPKVTLTLIALIWVVALILDTAPLYGFGEVAYEEYGACVPRFEG
EITYVVITMTLVIMQICIIIVTSTWIYCFTHKFIQEHSQLADDVYVSRNRRLVGIFCAML
IG

>PAC:15710682 pep:novel scaffold:Aqu1:Contig12965:2:839:1
gene:Aqu1.212154 transcript:PAC:15710682 description:""
IANGVVLSITLYQRKSWKQSSTIFFTSLILAHLVLNLLYFPFTIIALAAGEWIFGSTDEE
KRGTCSFCSFTALCILLIIGMTVAAISFDRFLFIVKPHLHKRFMRPWVALTLTIAIWTLS
AVLSSTPFYGLGNFSFDELLGFCFPLWISVDFIVYSLVIILLLFSVIIVTSVWTLCFTHR
FITQQSQLSNDNIYASKKKWLLGIFGSMLLVYGIYFMPGVIVVILIPLIDVPIALIVASI
ICSFFITVANPIVQSYFRPEIKSAFLSLFKKPSPFVSV

>PAC:15710683 pep:novel scaffold:Aqu1:Contig12965:2968:3888:1
gene:Aqu1.212155 transcript:PAC:15710683 description:""
MENKFTFTGEFSGPAVAAVLTVEMILALIANGVVLSITLYQRKSWKQSSTIFFTSLILAH
LVLNVLYLPFTIIALAAGEWIFGSTDDEKTRTCSFCSFTALCILLIIVMTVAAISFDRFL
FIVKPHLHKQFMRPWVALTLTIAIWTLSAVLSSTPFYGLGNFNFDELLGFCFPLWISVDF
IVYSLIIALLLFSVIIVTSVWTLCFTHRFITQQSQLSNDDTYGSKKKWLLGIFGSMLLVY
GICFLPSVIIVILIPLIDVPIALVISSIICSLFITVANPIVQSYFRPEIKSAFLSLFKRP
SPFVSV

>PAC:15710684 pep:novel scaffold:Aqu1:Contig12965:6737:7678:1
gene:Aqu1.212156 transcript:PAC:15710684 description:""
METVEAVEANFTFAQEFSSPVVAAVLIIQVILALIANGVVLSITLYQRKSWKQSSTIFFT
SLILAHLVLNLLYLPFTIIALAAGEWIFGSTDEEKRGTCTFAAFVFVSSILAIFMTLAAI
SFDRFLFIVKPHLHKRFMRPWVALTLTIAIWTLSAVLSFTPFFGFGHFLYFSRIGSCFPI
WATLEYELYTLALVATNLLIIAVTSVLTFCFTRRFINNQSLITPNVYSCEKKRLFGIFGA
MLLSYVVCFIPSVILIFLFLFPFTMPVEYIVTCFVSVLFVTTVNPLIQSYFRPEVKAIIN
SMFFQKLSKKILV

>PAC:15710685 pep:novel scaffold:Aqu1:Contig12965:7829:8776:1
gene:Aqu1.212157 transcript:PAC:15710685 description:""
MEDNFTFTGNFSTPAVAAVLSVETVAGLVANFIILSITLYQRKSLKQPSTIFFTSLILAL
LVLLLVYLPLSIIAIAAEEWIFGSSFEEKSATCSFAAYIFWYCLMVITLTLDAISFDRFL
FIVKPRLHKRFMRPWVALTLTIAIWILSAILNSAPFFGLGVFRYESWFGSCIPVWISIDF
VAYSLTIIFFLLSVIVITSLWTFCFTRQFMNEQSLSTTPDAYTSQKKRLFGIFGSMLIVY
GICFTPGTIMIILIPIVDSPAELFVTSYVCILFITVANPIVQSYFRPEIKSTLLALCQRK
QANQFQSLELSKHSM

>PAC:15710686 pep:novel scaffold:Aqu1:Contig12965:9018:9935:1
gene:Aqu1.212158 transcript:PAC:15710686 description:""
MNNFTYNGDFNNVAVGAALAVEGVSGFLANTFVLSITLYQRKSWKQSSTIFFTSLILANF
VMVLLHFPFAVTALAAGEWIFGSTDEEKTGTCTFAALTFWYSSIVIILTLAAISFDRFLF
IVKPHLHKQFMRPWVALTLTIAIWTLSAVLSSTPFYGLGNFSFDELLGFCFPLWISVDFI
VYSLVIILLLFSVIIVTSVWTLCFTHRFITQQSQLSNDDTYGSKKKWLLGIFGSMLLVYG
ICFMPAVIAIILIPVIEVPIALVISSIICSLFITVANPIVQSCFRPEIKSAFLSLFKKPS
PFVSV

>PAC:15710687 pep:novel
scaffold:Aqu1:Contig12965:10196:11158:1 gene:Aqu1.212159
transcript:PAC:15710687 description:""
MESNFSFTGSFNRPVAITVLSITTLAALIANSVVLFITLYQRKSWKQSSTIFFTSLILSN
FILNLLYLPFTIIALAVGEWIFGSTDEEKRGTCAFAAFIVCYTVLVQSFTLAAISFDRFL

FIVKPHLHKRFMRPWVALTLTIAIWILSALFGSTPFYGLGKFAYLPNVGTCSPAFTEYFF
VGYSAAIEVAVFLFIIITSVWTFCFTYKFIRDQSAIGEESVYLSRKKRLVGIFGAMLLVY
VICLLPIFTVAILFPVVDISSHVVASVVFVLHLFTVINPLVQSYFRPGFKDLILTVVMKW
PKRRLTNAMLRLNRMPNQSN

>PAC:15710688 pep:novel
scaffold:Aqu1:Contig12965:12342:13573:1 gene:Aqu1.212160
transcript:PAC:15710688 description:""
METVEAVEANFTFAQEFSSPVVAAVLIIXXXXANTFVLSITLYQRKSWKQSSTIFFTSLI
LANFVMVLLHFPFAVTALAAGEWIFGSTDEEKTGTCTFAALTFWYSSIVIILTLAAISFD
RFLFIVKPLLHKRFMRPWVALTLTIVIWILSAVLSCTPFYGLGNFGFTASIGLCIPLLVK
GGFVILAFTILILSLLIIVITSLWTFFFTWWFLRKRSMVVESNIYSSKKRGLLGIFGFML
LVYGISKGPRIIALVLVQFEVPFSSTSQIVIYFVYQLSIIGDPIVQSFFRPGLKQAMVSL
FKNCKK

>PAC:15710689 pep:novel
scaffold:Aqu1:Contig12965:15638:23368:1 gene:Aqu1.212161
transcript:PAC:15710689 description:""
MSNFTYNGDFSNVAAGAALAVEGVSGFLANTFVLSITLYQWKSWKQSSTIFFTSLILANF
VMVLLHFPFAVIAIAAGEWIFGSTDEEKTGTCTLAALTFWYSSLVLVITLAAISFDRFLF
IVKPHLHKRFMRPWVALTLTIAIWILSAVLGSTPFYGLGGFGFNTSIGMCLPFLIKDIIL
AFLILIISLLLIIVITSLWTFFFTWWFLHRRSMVVESNIYLSKKRGLFGIFGAMLLVYGI
SKGPRIISLVLVQFEVPFSSTAEIAIYFVYQLSIIADPIVQSFFRPGFKQAMVSLFKKCK
NNVAVGAALAVEGVSGFIANTFVLSITLYQRKSWKQSSTIFFTSLILANFVMVVLHFPFT
IIALAAGEWIFGSTDEEKRGTCTFAAFTFWYSSLIIVMTIAAISFDRFFFIVKPHLHKRF
MRPWVALTLTIAIWILSAVLSSTPFYGLGDFGFIASIGLCLSFLIKDGSVILAFIILILS
LLIIVITSLWTFFFTWWFLHRRSMVVESNIYSSKKRGLFGIFGAVLLVYGISKGPRIIAL
VLVQFEVPFSSTALIMIYLVYQLSIIADPVVQSFFRPGFKQILGRPIAMYITVISLLQQQ
TIANAASSHNSALLVAEHHKRVVHLACLEEAALIMNNFTYNGDFSNVAVGAALAVEGVSG
FIANTFVLSITLYQWKSLKQSSTIFFTSLILANFVMVVLHFPFAVTALAAGEWIFGSTDE
EKIGTCTFAAFTFLYSSVVIILTLAAISFDRFFFIVKPLLHKQFMRPWVALTLTIVIWIA
AAVLSSTPFYGLGDFGFVASIGLCIPLLVKDGFVILAFTILILSLLIIIITSVWTFFFTW
WFLRKRSMVVESNIYLSKKRGLFGIFGAMLLVYGISKGPRIILLVLVQFEVPFSGTALIV
IYFVYQLCIIADPIAQSFFRPGFKQAMLAWKKLLVMNNFTYNGDFSNVAVGAALAVEGVS
GFIANTFVLSVTLYQWKSLKQSSTIFFTSLILANLLMVLLHFPFTIIALAAGEWIFGSTD
EEKTGTCAFAAFTFWYSSLVLVMTVAAISFDRFLFIVKPHLHKRFMRPWVALTLTIAIWI
LSAVLSSTPLYGLGGFGFNASIGMCLPFLIKDASVISAFLILILSLLLIIVITSLWTFFF
TWWFLHKRSMVVESNIYSSKKRGLIGIFGAMLLVYGISKGPRIISLVLVQFDVPFSSTAE
IVIYFVFQLSIIADPVVQSFFRPGFKQVLVSLFKKCKK

>PAC:15710691 pep:novel
scaffold:Aqu1:Contig12965:26889:27827:1 gene:Aqu1.212163
transcript:PAC:15710691 description:""
MDEVNDTLYTFTGSVNTYVLAAVFVLEFIAGFIFNTIVIGITIYKGSWKKQGTIFFTSLI
LANLLLVILYIPFLVIGLAARKWIFGNTVAEKLASCWFVGFALWYSFIIILMTLAAISLD
RFLFVIKPHFHKWFMRPWVTLTLTIAIWILSAVLSSTPFYGLGTYAYGRWYGSCVPLWVE
IGFIIYSVTISLLVIAVIVITSVWVFCFTRIFIKVHPEVEVPADPDTVICLSKKKRLFGI
YGSMLLAYTVCFAPTLISSIISSSIDIPGEVFFLNVITFFLITVVSPLIQSYFRKDIKEV
IVLCYKKITKKQ

>PAC:15710692 pep:novel
scaffold:Aqu1:Contig12965:28337:29296:1 gene:Aqu1.212164
transcript:PAC:15710692 description:""
MEYHNFTATGDVNGPVLAAVLAVEAVVAFIANIIVLSITLYQRKSWKQSSTIFFTSLILS
NLLAVVGYLPMNIVAIGAEEWIFGSTFEERSITCLISALIIQLNIAVTTEILAAISFDRC
LFVVKPHLHKRFMRPWVALTLTIAIWIFCILLLMLPLLGFGKYSYEFSYGPCIPMLIKSP
VYSFVHAALHIISFAVIIITSIWTCCFTQRFINDQSEIVGDNSVYASRKKKLFGIFGSMF

LAYSFCYGPSYITLMLYPVVEIDEIVYAADMVTFQFITVASPLIQAYFRPDIKNALIWFK
KSIVSQIGKRLYSPPQNEQ

>PAC:15710693 pep:novel
scaffold:Aqu1:Contig12965:30227:31309:1 gene:Aqu1.212165
transcript:PAC:15710693 description:""
MSIQSENNFTLNDDVNGPVLAAVFATEMILALIANGVVLLITITQRNSWKQSSTIFFTSL
ILAHLVLNLLYLPFTIIALAAGEWIFGSTDEEKRETCDFVAYTLWYSIPVITITLAAISF
DRFLFIVKPHLHKQFMRPWVALTLTLAIWILSAVVTFTPFIKGSGAAFIYEGSHGTCTII
LLELEFAIVCFLGSLLVVSIIVVTSVWTFCFTRKFIHNQSEIAGDNVYASRKKRLFGIFG
AMLIVYGLCFTPGIINYAVVRVIFVDDRIYLAIVISFIFVTVLSPIVQSYFRPEIKNKIV
FICRKILQKPPQRDRVNYPVSSNNNSSGSKTEINRIGTSTQLTMSLDNALNTEHSSTTVV

>PAC:15710694 pep:novel
scaffold:Aqu1:Contig12965:44866:45948:1 gene:Aqu1.212166
transcript:PAC:15710694 description:""
MSISSENNFTINDDVNGPVLAAVFATEMILALIANGAVLLITITQRNSWKQSSTIFFTSL
ILAHLVLTLLYLPFNIIALAAGEWIFGSTDEEKRGFCSFISYTLWYSIPVITITLAAISF
DRFLFIVKPHLHKRFMRPWVALTLTLAIWILSAVTTFTPFIEGSGAVFMYEGSHGTCTVV
ILELHFAIFSFVGSLLVVGIITVTSVWTFCFARKFIHNQSEIAGDHVYASRKKRLFGIFG
AMLIVYGLCFTPGIINYVVVRAIVVADEIYLAVVISFIFVTVLSPIVQSYFRPEIKNKIV
CICRKILQKPPQRDSVDYLGNSNNDSSGSKTETNQIRTSAQLTMSLDCVLNTEHLSTTVV

>PAC:15710695 pep:novel
scaffold:Aqu1:Contig12965:48640:49623:1 gene:Aqu1.212167
transcript:PAC:15710695 description:""
MEGNFTFTGDFNTPAVAAVLTVEMILALIANGVVLSITLYQRKSWKQSSTIFFTSLILAH
LVLNLLYLPFTVIGFAAGEWIFGSTDQVKRGFCKFNAYILWYSILNIIMMLAAISVDRFL
FIVKPHLHKRFMSQPKVALTLTIAIWLLAAVLSSTPFFGLGEFGYETWFGGCLPLWTTIE
FTVYTIIVCLVIIFIISLTSIWTFCFARRFLTDQSRLTTENNVYYSSKKQRLIGIFGAMS
LVYLLCFSPGIISAFLTPLTLVPYEVIVLVIVTIELLTIAGPLVQSYFRPDINKVLVSLY
TKMRRLFRRNMQSVCSNRELDTKISYV

>PAC:15710773 pep:novel
scaffold:Aqu1:Contig12972:28306:29304:-1 gene:Aqu1.212245
transcript:PAC:15710773 description:""
MERNYTFTGDFSPEAVAGVLSIEMILALIANGVVLVITIYQRKSWKQSSTIFFTSLILAH
LVLTLYLPFFIAALAAGEWIIGSTDGEKKATCDFNGFIIIHSGYVMLMTLSLISIDRFLF
IVKPHLHKRFMSPRVALVLVIIVWIVTALFFSLGFIDGSGVVFRYIDNVGGCYVYTTSPV
TSIIRYLFASLIFCTIVVTSIWTFCFTRMFMNNQSMIVGESVYASKKKRLFGIFGSMMVV
YCIFFTPAIFFSALLAIVDSPGVLSMTALIFFFLVVVLNPVVQAYFRPEINSVIVNIVCC
KILKKKPNPATTLSTFNSLSATLEFSVSSLDV

>PAC:15710774 pep:novel
scaffold:Aqu1:Contig12972:34323:35321:-1 gene:Aqu1.212246
transcript:PAC:15710774 description:""
MERNYTFTGDFSPEAVAGVLSIEMILALIANGVVLVITIYQRKSWKQSSTIFFTSLILAH
LALTLYLPFTIAALAAHEWVVGSTDGEKQGTCSFTAFVILLSVYVMLMTLSLISIDRFLS
IVKPHLHKRFMSPRVALVLVIIVWSFDLLFFSTGFIEDSGVEFRYIDYLGICYAFTTSPV
MAIFRFFFASLIFIIIIVTSVWTFCFTRKFINNQSMIVGESIYASKKKRLFGIFGSMLLV
YGIFFTPSIFFSALLAIVDSPGVLTISALMFFFLVVVLSPVVQAYFRPEINSVIVNIVCR
KILKKKHNPAATSSTCNSSSAAVELRVSSLDV

>PAC:15710775 pep:novel
scaffold:Aqu1:Contig12972:36940:38446:-1 gene:Aqu1.212247
transcript:PAC:15710775 description:""
METNYTFTGDFSPEAVAGVLSIEMILALIANGVVLVITIYQRKSWKQSSTIFFTSLILAH

LVLTLFLPFFIAALASGEWIIGDTDEEKKATCGFTAFVMIYCGYVMLMTLSLISIDRFLF
IVKPHLHKRFMSPRVALVLVIIVWIVTAVLNSSGFINGSGVFGRFVSLSMIVGESVYASK
KKRLFGIFGSMLLVYGICVSPIIFFSALLAIIDSPGVLGITAVIFFFLVVVLSPVVQAYF
RPEINTVIANVIVNIACHKMSKKKPNSATTSSSSSAAVELRVSSLDFSLVIERMERNYTF
TGDFSPEAVAGVLSIEMILALIANGVVLVITIYQRKSWK

>PAC:15710776 pep:novel
scaffold:Aqu1:Contig12972:43001:44234:-1 gene:Aqu1.212248
transcript:PAC:15710776 description:""
MITVDCSNFSCSSSTFGELSLANEAEFSLVIERMERNYTFTGDFSPEAVAGVLSIEMILA
LIANGVVLVITIYQRKSWKQSSTIFFTSLILAHLVLTLYLPFSIAALAAGEWIIGETDED
KEGTCHFNGFIILFSAYIVFMTLCLISIDRFLFIVKPHLHKRFMSPRVALVLVIIVWIVN
AAFYSAGFIDGSGIVYQYVVWTFCFTRKFINNQSMIVGESVYASKKKRLFGIFGSMLLVY
GICFIPAIFFGTLLAIIDFPVVLGITALIFFFLAVVLSPVVQAYFRPEINSVIVNIVCRK
ILKKKPNTLATSSTCNSSSAAVELRVSSLDV

>PAC:15710777 pep:novel
scaffold:Aqu1:Contig12972:48557:49546:-1 gene:Aqu1.212249
transcript:PAC:15710777 description:""
MERNYTFNGSFSPEAVAGVLSIEMVLALIANGVVLVVTIYQRKSWKQSSTIFFTSLILAH
LVLTLSLPFSIAALAAREWVVGDTDEEKQGTCGFTAFMILFSVYVMLMTLSLISIDRFLF
IVKPHLHKRFMSPRVALVLVIIVWIVPAAFFSSGFINGSGAEFRYIDYLGVCSAFTTSPV
MAIFRFFFASLMFGRFASLSMIVEESVYASKKKRLFGSMLLVYGICFTPSIFFSTLVAIV
DSPGALTLTALMLFFFAVIPSPVIQAYFRPEINSVIVDIVCRKMLKKKPNKLATSSTCDS
SSAAVELRVSSLDP

>PAC:15710836 pep:novel
scaffold:Aqu1:Contig12980:17612:18616:-1 gene:Aqu1.212308
transcript:PAC:15710836 description:""
MESNYTYTGDFSPEAVAAVLSIEMILALIANGVVLVITIYQRKSLKQPSTIFFTSLILGH
LVMILYLPFSITALAAGEWIIGSTDEERQGSCDFSAFIILYCVYVMLMTLSLISIDRFLF
IVKPHLHKRFISPRVALVLVIIVWVFNAAFFSSGFIDGSGVVYQYIPDLGVCYAYTISTI
AAVIRFLLTLVLLCIIVVTSVWTFCFTRKFINDQSEIVGDCVYASRKKRLFGIFGSMLLI
YGVCFIPSAVLSSFLAIADPPGALVVSALILFFLAIVLSPVVQSYFRPEINSVIVNIICH
KIMKKPHTTTSSMCNNSSANRGTNIDSLDINLHA

>PAC:15710930 pep:novel
scaffold:Aqu1:Contig12992:13727:14362:1 gene:Aqu1.212402
transcript:PAC:15710930 description:""
MNNSSTTSTREELHAAVIAAVLTVEMILAVIANGVVLSITLYERKSWKQSSTIFFTSLIL
AHLVLILLYLPFTIIALAAGKWIFGSTDEEKTTTCTFVFYLYWYIIAVISMTLAAISFDR
FLFIVKPHLHKRFMRPWVAQTLTIAIWLLSAIXXXXXXXXXXXXXXXFFVFIAVANPVVQ
SYFRPDIKTFCFSHCPLLSTCLCCSCLHNVF

>PAC:15710932 pep:novel
scaffold:Aqu1:Contig12992:24170:25111:1 gene:Aqu1.212404
transcript:PAC:15710932 description:""
MEGNFTFTGEFSGPAVAAVLTVEMILALIANGVVLSITLYQRKSWKQSSTIFFTSLILAH
LVSNLLLNLPFTIIALAAGEWIFGSTDEEKTGTCDFAAWMLWFGASCMSMTLAALSFDRFL
FIVKPHLHKWFMRPCVALTLTIAIWILSAVLCALPFIDIGNYSYDNDIGICTIVGVDIAF
FILILVIIFLIVGTIFVTSLWTFCFTRSYFKAQSVIAGESVYASKKKRLFGVFGSMLLIY
GISHLLVALWVLLQIFIFLPYEFFVIVYIVYFFVTIASPIIQSYFRPEIKSVLVSRCPLL
FTCVCCSYVHAVC

>PAC:15710934 pep:novel
scaffold:Aqu1:Contig12992:27942:28865:1 gene:Aqu1.212406
transcript:PAC:15710934 description:""

MEGNFTFTGEFSGPAVAAVLTVEMILALIANGVVLSITLYQRKSWKQPSTIFFTSLILAH
LVLNLLYLPFSIIAFAAGEWIFGSTDEEKKTTCTFTSFLYSYTMRIIFMTLAAISFDRFL
FIVKPHLHKRFMRPWVALTLTIAIWILAAVLSSVPFYGLNEYVYDDQLLLCYQRPNSTIS
LIIFNVLFPCVTTAIIIVTSLWTFCFARSYFKAQSLIAGESVYASKKKRLFGVFGSMLLI
YGMGIVPGLVLLSLQHFVDVPPELFISFVIFLFFITIASSIIQSYFRPEIKSVLASRCPL
LFYCACC

>PAC:15710937 pep:novel
scaffold:Aqu1:Contig12992:44064:45005:1 gene:Aqu1.212409
transcript:PAC:15710937 description:""
MEGNFTFTGEFSGPAVAAVLTVEMILALIANGVVLSITLYQRKSLKQSSTIFFTSLILAH
LVSNLLNLPFAIIALAAGEWIFGSTDEEKRGTCIFAAFILWFGASFISMTLAAISFDRFL
FIIKPHLHKRFMRPWVALTLTIAIWILSTVLCALPFIDIGHYSYHDDLGICTIDGVDIAF
FVVILVSIFLIVGTIFVTSLWTFCFTRSYFKDQSVIAGESVYAFKKKRLIGVFGSMLLIY
GISHLLVALWVLLQIFILLPSEFSVTSYIVYFFVTIASPIIQSYFRPEIKSVLVSRCPLL
FTCVCCSCVHAVC

>PAC:15710938 pep:novel
scaffold:Aqu1:Contig12992:45265:46197:1 gene:Aqu1.212410
transcript:PAC:15710938 description:""
MEGNFTFTGQFSGPAVAAVLTFEMILALIANGLVLSITLYQRKSLKQSSTIFFTSLILAH
LVLNLLYLPFTIIALAAGEWIFGNTDEEKIATCTFTAFLYSYTIRIIFMTLAAISFDRFL
FIVKPYLHKWFMRPWVALTLTIAIWILSAVLSSAPFYGLNEYVYDDQLLLCYQRPNSTIS
FIFYVLFPCVTTAIIIVTSLWTFCFAGSFFKDQSVIAGESVYASKKKRLFGVFGSMLFIY
GICIVPGLVLRSLQEFIDVPPKLSISSMTCFLFITIASPIIQSYFRPEIKSVLASRCPLL
FTCACCKFIH

>PAC:15710939 pep:novel
scaffold:Aqu1:Contig12992:46555:47452:1 gene:Aqu1.212411
transcript:PAC:15710939 description:""
MAKNFTFTGEFSGPAVAAVLTVEMILALIANGVVLSITLCQRKSLKQPSTIFFTSLILSH
LVFNLLYLPFTIIALAAGEWIFGSTDEEKTATCTFTAFIYSYTIPVIFMTLAAISFDRFL
FIVKPHLHKRFMRPWVALTLTIAIWILSAVLSSTPFYGLNEYVYDDHQSYYPVIL

>PAC:15710940 pep:novel
scaffold:Aqu1:Contig12992:51121:52032:1 gene:Aqu1.212412
transcript:PAC:15710940 description:""
MESNFTFTGEFSGPAVAAVLTVEMILALIANGVVLSITLYQRKSWKQSSTIFFTSLILAH
LVLNLLYLPFTIIALAAGEWIFGSTDEQKTGTCYFNLFIFWWMVVTIFLMLTTISFDRFL
FIVKPHLHKRFMRPWVALTLTIIVWLLAAVSNIIYLLTFHSDIDPDLCYLLPINVNRSSV
FAIVSTVIVLIIMFVTSLWTFCFTKSYFKAQSVIAGESVYASKKKRLFGVFGSMLIIYGI
CFVPAVVYLIAFLFVDVPVWYSFTAFICYLFITVANPVIQSYFRPEIKSFFVSHSLHHFH
ICC

>PAC:15710941 pep:novel
scaffold:Aqu1:Contig12992:54553:55494:1 gene:Aqu1.212413
transcript:PAC:15710941 description:""
MESNFTFTGDFSGPAVAAVFTVEMILGLITNGTVLFITLYQRKSWKQSSTIFFTSLILSH
IVINLYLSTTIVALAAGGWIFGSTDEEKTWTCIFAAWLLWSALAVLSYTLVALSFDRFLF
IVKPLLHKRFMTPRVALTITIVIWIAAAVLNLWPVLHNFEHYGYLNDLGSCSIIGVDLAA
FVVFLISNFIIFGSIAVTSLWTFCFTRSYFKAQSVIAGESVYASKKKRLIGIFGCMLIVY
GLFYLQSVVSFLLQLFIVVPNQFFLALSVTFSLVTIASPIIQSYFRPEIKSVLVSRCPLL
FTCVCCSCVHSIC

>PAC:15710942 pep:novel
scaffold:Aqu1:Contig12992:57178:58116:1 gene:Aqu1.212414
transcript:PAC:15710942 description:""

MESNFTFTGEFSGPAVAAVLTVEMILGLITNGTVLFITLYQRKSWKQSSTIFFTSLILSH
IVLNLQLSFSIIALAAGGWIFGSTDEEKTWTCIFAAWLLWSALAVLSFTLVALSFDRFLF
IVKPHLHKRFMIPRVSLTITIVIWIAAVILNLWPILHNFEHYGYLNDLGGCSIVGVDIAA
FVVFLVSNFLVFGSITITSLWTFCFTRSYFKAQSVIAGESVYASKKKRLIGIFGCMLIVY
GLFYLQSVVSFFLQLFIVVPNEFFLALFLTFSFVTIASPIIQSYFRPEIKSVLVSCCPLL
FTCVCCSCVHAV

>PAC:15710943 pep:novel
scaffold:Aqu1:Contig12992:60240:61184:1 gene:Aqu1.212415
transcript:PAC:15710943 description:""
MESNFTFTGKLSGPAVAAVHTVALILALIANGIVLSITLYQRKSWKQSSTIFFTSLILAH
LVLNLLYLPFTIIALAAGEWIFGSTDEEKTGTCAFAAFILWFGACVISMTLAAISFDRFL
FIVKPHLHKQFMRPWVALTLTIAIWILSASVFGSLPFLDIGHYSYDDELGFCILIDVKIA
SFVVILVIVLLVVGTIFVTSLWTFCFTKSYFKAQSVIAGESVYASKKKRLFGVFGSMLLI
YGTAYLFVALGILLQIFISPPYELYISYYIVFFFVTIASPIIQSYFRPEIKSVLVSRCPL
LFTCVCCSCVHAVC

>PAC:15710944 pep:novel
scaffold:Aqu1:Contig12992:62266:63210:1 gene:Aqu1.212416
transcript:PAC:15710944 description:""
MESNFTFNGEFSGPAVAAVLTVVMILALIANGVVLSITLYQRKSWKQSSTIFFTSLILAH
LVLNLLYLPFTIIALAAGEWIFGSTDEEKTGTCIFVAFILWFGASVITITLAAISFDRFL
FIVKPLLHKQFMRPWVALTLTIAIWILAAVLNFWPVIHNFEHYSYNYELGYCTLVGVDIA
AFIVFLVAIFFIVGTIFVTSLWTFCFTKSYFKAQSVIAGESVYASKKKRLFGVFGSMLIV
YGTSYLLTAFSFILQLFIVVPNEYYVMLTIAFPIVTIASPIIQSYFRPEIKSVLVSRCPL
LFTCVCCSCVHSIC

>PAC:15711746 pep:novel
scaffold:Aqu1:Contig13069:63737:64684:1 gene:Aqu1.213218
transcript:PAC:15711746 description:""
MDIHGLNYTLGEDVNGPLLAAALAVEMIAALIVNTFVLVATFSQCKSLKLPSTILFTSLI
MIHYVMALIYIPSWLISAAYGEWIFGNTMQVKEATCNFAGFILNYNITFINATLAAISVD
RWLFIVKPIFYKQYMKAKVALIVVASTWITSTLLNVPPFFEMGKYVFSPFGSCEVKFENE
ASFSFLLLVVFFTEASIIIVSSIWTCCFTRRFIREHAQLADESAYVSKNRRIIGIFGAML
VAYGVCYTPAVIVIVTSLFHDVPASYIAASLVFLLTVIIINPIIQSIFRPDVKKVIVKLC
TICRKHQYSPEEPQP

>PAC:15711978 pep:novel
scaffold:Aqu1:Contig13087:72248:73198:-1 gene:Aqu1.213450
transcript:PAC:15711978 description:""
MDVFGLNYTLAEDVNGPLLAVVLTLELIAALVTNTIVLAATLSQQKSLKLPSTILFTSLI
MIHYVMAFIYILSWLISVISGGWIFGTSEEEKEATCNAAGFVVCYSLSVINATLTAISVD
RWLFIVKPNFYKQYMKPKVTLVLVLSIWIFSGLTFITTFFGIGGFVFTTLGSCGPKFKDE
TGFTILLLAIFFPEISIVIVTSVWTYCFTKKFIREHAQLAENNVYVSKNRRLTGIFGLML
IAYVICYVPSLIPIILNQFHDVPAMWIAFGLVCILAMTFINPIIQSFFRREVKEEIKKFC
NIIKCCPHRSNAIMAG

>PAC:15712451 pep:novel
scaffold:Aqu1:Contig13121:67635:68712:1 gene:Aqu1.213923
transcript:PAC:15712451 description:""
MDDINNNFTLSQDVNGPLLAAVIGMEMLAGLITNSFVLILTACHLKNWKQPTTVFLSNML
ANNLVVILFTMPLSIITTASGEWIFGSTVSQKESVCYFAAFIFIFSILTATESLVLLSFD
RFFFIVKALHYKKYMTVNRAFIIVAVSWLLAAFLSMLPFFGFGAFEFASSYGMCVPGWTG
QAGYAIFSFIVISIFIGSITVTSLWTMCFTRKYLKNAATNISTAASPAAYAVAFVVLLLL
TVLIPLVQSFFRRDIRDAIVKGHRTFFRSIKQEKTYHQSSSTQTVKPSNATTSSYTI

>PAC:15712452 pep:known

scaffold:Aqu1:Contig13121:72652:76703:1 gene:Aqu1.213924
transcript:PAC:15712452 description:""
MDDINNNFTLSEDVNGPLLAAVIGIEMLAGLTTNSFVLILTACHLKNWKQPTTVFLSNML
ANNLVVILFTMPLSIITTATGEWIFGSTVSQKESVCYFAACIFIFSILTATESLVLLSFD
RFFFIVKALQYKKYMTVNKAFIIAAVSWLLAAFLSMLPFFGFGAFEFSFSYVISIFIGSI
TVTSIWTMCFTRKYLKNAATNISTAASPGNPYAAQERRVIGLFAAYAVAFVVLLLLTVLI
PLVQSFFRRDIKDAIVKGHRTLSRSIKRGKTYHQSSSAQTLRSACGLFNHKASHLSFLNS
IMSLSGESDKRVDPTTNKVVLVGDETVGKTSMFLRFKTDRFIETTSSTRYLAEHYKEWTV
RGTKVQMTLYDTAGMERYTSTIPPTYFRHARAVILVYAVDNQESIGNIRNWSESFSKHRI
GDTVDSLKVLLVGNKVDLNRTVSANRVNEVAEFCGIDNSLKYEISTKNNDGGFDELFDDL
AYLLSDTPIERRKTIRATSSPDDEEMKKKKALCSKCS

>PAC:15712786 pep:novel
scaffold:Aqu1:Contig13148:95889:96875:1 gene:Aqu1.214258
transcript:PAC:15712786 description:""
MSDVFYNFTESNEGVYGPAVAVILGLEMVAGLFANAVVLCITLMQRKSLSQPSTIFFTSL
TAAHLLMVLLYLPVSVIAIGAEEWIFGSNFEEKKITCSISAFVFWYSVLLMTINLAVISF
DRFLFIVKPHFYKQFMRPTVALILTIAVWILSAALNSTPLYGLGAYQYGPSYGSCIPTWR
NVPGYLSFMAAIFLIVLAFIIVTSLWTFCFTRKFMHEQEPSQQSNVYQSKERRLFGIFGA
MLIVYGVCFLPSVITGIMTIFVILPGQVYAMDMILFQLITSGSPLVQSYFRPDIKERLTF
IYKRSLQMMKNIFYSRKKNNLSQDEQLS

>PAC:15712925 pep:novel
scaffold:Aqu1:Contig13160:52203:53790:1 gene:Aqu1.214397
transcript:PAC:15712925 description:""
MDDDLMSGSGNGLINETEFDFSCRTSVLSTTNVTDQEFWTFRGNLGSGTIAIAFFESIIF
IVALSWNLFIFVTYLLKYRLLKEPANIMLFTLSIVDLLVCILIIPFPIIVVAANGEYIFG
NSDVVRCIICQVQGYFFILSTELSLHLLAILSIDRCILLSNPLKYKDIKKVWTTVVGILV
IWVFCFLLALPPAFGFGEWEFNRSFGVCIPRWTPFRNSLYMILLMIEGLIPIITLAVTNV
WTFKIVNRFLKKNLERKKSFRATREEVAVEKSTHRSQQNQLVKVFGALFIANIISWTPLL
SLTFAIAATDGDGIPAWLLIVGWFMFLLNPTVHPILESFFIKELRTRVNRASNSVKQQVR
RASRSILKMATLDSFKDIPTMNDEDESEKSRRVFGLKRKSTSQSIGNASVNTSMTDASPP
DSPMGITRANTLTNSGRFPHRNRRSSSPKDNIIGTEILPPSPLLTPVSAALPRISEDSQL
ALTDIAKSQNNVKPKASSITDLSGKKKKRHISITVPGEKDVYRPEDRDS

>PAC:15712931 pep:novel
scaffold:Aqu1:Contig13160:69230:70549:1 gene:Aqu1.214403
transcript:PAC:15712931 description:""
MDFFGSGDDGLNATSNYTTNYTDFDCLKSELDPNGITNRTFWSLRDTVVDGGIPSAVIQS
LILAVALGWNLFIIVVFILKRELLKEPANILLFTLAIVDVLICLIVVPGPIVVTAANGEF
VLGRNDQIRCAICDTQGFFFIFLTVLSVHTLALLSIDRCILLSNPMKYPRYRKVGLWVLF
IVLVWLLCLIISMPPAFNVGFGQWEFNRNFGLCLPRWIPPTYMGFVFLEGLIPITVIIIT
NIWTFKIILSFLKRKQVRRRESRLTSLSRKESEAEVQIKQQQKQLVQVFGALLIATIIAW
VPLLTMLFVLIGTDGEGVPDWIYLVCWFFYLINPLVHPILESLFVKELRTRIDKTKTNIR
RASQSVMRLASRSTIKDIPEMSESTPYTKSRVFSFSSNPYHSERKSDSLNGSIRNGSPPA
NDDNDNVFTPTSKVRFSIV

>PAC:15713478 pep:novel
scaffold:Aqu1:Contig13191:66817:68024:1 gene:Aqu1.214950
transcript:PAC:15713478 description:""
MENANFTLSDEINGPLLAGAIGIVTVVALLTNSFVLLLTLCHFKAWKQPSNIFLTNMLLS
NLLISVFLMPLCVVTCATGEWITGATDAQKLKTCQATAYIFLYNLIVETESLTLISLDRL
FFIVKSMEYHKYMSTKKALLIVLLSWLLAAILSTPPFYGLGSFEFAESYGICVPGFEGQL
GFSIYLFLILLSLGGIIVISSTWTFCYTRNFLRRRNSRKTARNSVYLSQKRKLIGLFGTL
VVIHILCYSLMLSLAVFGPFFTISPPWYAAAFFMLLLMTNLSPLAQSYFRYEVRNFVHSL
FIKIGIVKLRASTLVQQERTATTELPSPTETRRDTIHSTNCQFLNGESEIDKHVV

>PAC:15713938 pep:novel
scaffold:Aqu1:Contig13214:46818:48119:1 gene:Aqu1.215410
transcript:PAC:15713938 description:""
MDSLDDDQVLSANENFTISPSISGPAIAFFLSLEMGISLAINSTILGIIFLHPKPRSLIK
APSNIYLTSMLLVNLLATLTVMPMIIIASASGEWIFGGTLEEKVSSCRFAGYMYWYVIFL
LIVTLAIISVDRWMFIVKSTLYKKMMTTPVAIGVVITAWLLTAVLNTTPLYGFGMFAYSS
SPGSCFPVWKDQKAYLAFFVLVFLILISIIAASSIWTFCFSRRFIRRMSLRNIDASGEAA
SVYTFRWRKLIGIFGTLSIVYVVCFTPSLLYLLIRLATPIPLQILPAILIFFLLITILNP
LVQIFFRRDIKNVILDFLAKTPCYTKEEVDYEHRSRTRSNFELQHALSLTAMGKDVANGG
SALDVSPDEGHSSDPKSSGKVVVKFEDSESVPVQANGATSVGGDATNDSASSESVSNGVS
KTNGGDILGSTAV

>PAC:15713939 pep:novel
scaffold:Aqu1:Contig13214:48793:49833:1 gene:Aqu1.215411
transcript:PAC:15713939 description:""
MDYEDDETLFILEENYNFTLNPLINGPALASIVGLELFAGFITNSLLLALTVFQWKNWKL
PSTVFLTNLLLCNLLLVLLVMPFTIITGATGEWVFGRNPVEKRTVCRIIAFIFWYGITVF
TFGLVILSFDRFFYVVKAVAYKNRMTIKKAVTIVIATWVAGGALNITPFFGMGKYSFIAC
CGVCVPRWAGEPEYIVYILIIYVTCILSIVVTSIWTCIFTRRFLGKDERRTRFVSQNTRS
HLYATKNQRLLGLFGMTVLVHLLCYLPGISSSFLELFIPVPVQLYTTMYVAFLMAATLGP
LVQSFFRRDIRETLSQLCGEKFKVFAVNRSFISSSSIKVNTNSTCL

>PAC:15713940 pep:novel
scaffold:Aqu1:Contig13214:53879:54397:1 gene:Aqu1.215412
transcript:PAC:15713940 description:""
MIGGLIANSFVLILTICHIKTWKQPSTLFLTNMLISNLLIVLFVRPFSITTAASGEWLFG
KTYKQKVKVCQFTAFIFWFCVIVITEGLVLLSFDRFFYIVKSFEYERHMNRKISIIIVTL
SWLLAALLTIPPLFGLGRFGFSSSYGICVPHWEGESGYVVYMLMVFIIFIKS

>PAC:15713942 pep:novel
scaffold:Aqu1:Contig13214:57845:58461:1 gene:Aqu1.215414
transcript:PAC:15713942 description:""
LRKMEDMDENFTLSGDINGPLLAAVISIEMIAGLIANSFVLILTICHIKTWKQLSTIFLT
NMLISNLLIVLFVMLFSITTAASGEWLFGRTYKQKMKVCQFTGFMFWFCVIVITEGLVLL
SFDRFFYIVKSFEYERHMNQKISIIIVTLSWLLAALLTIPPLFGLGRFSFSSSYGICVPH
WEGESGYVVYMLIVFIIFIISLHN

>PAC:15713944 pep:novel
scaffold:Aqu1:Contig13214:61345:62064:-1 gene:Aqu1.215416
transcript:PAC:15713944 description:""
MLYFKACGYRLVLKNKEEESMDAVHNNFTLSPDINGPLLAAVISIEMIGGLIANSFVLIL
TICHIKTWKQPSTIFLTNMLISNLLIVLFVMPFPVITSAFGEWIFGSTLDQKESVCKFTA
IMSLFCVAVATEGLALLSFDRFLFIVRAFQYNRYMSINKSLIAISISWAIAGIISIVPVL
EFNVYEFAYSFWYLCSWFRGTGWFCSICFYLSSTASGEYHSNFSMDVHIYEEISQNKKD

>PAC:15713945 pep:novel
scaffold:Aqu1:Contig13214:62301:63302:1 gene:Aqu1.215417
transcript:PAC:15713945 description:""
MEDENNFTLSGDINGPLLAAVISIEMIGGLIANSFVLILTICHIKTWKQPSTIFLTNMLI
SNLLIVLFVMPFAITTAASGEWLFGKTDKQKVKACQFTAFMFWFCVIVITEGLVLLSFDR
FFYIVKSFEYERHMNRKISIIIVTLSWLLAALLTIPPLFGLGRFGFSSSYGICVPRWEGE
PGYVVYMLIVFIIFLLSIIITSSWTMIYTRRYLNMERQRLQLFDVNNDGNTNDIYASRKR
RVIGLFGMIMIVHLLCYLPSMIVALMELVTAPPPQLYATIFLLFLLLTVLSPLVQSFFRR
DIRGTVVKLTGWCSEVAKLFNKKYTRIASSSEN

>PAC:15713946 pep:known
scaffold:Aqu1:Contig13214:63443:64519:1 gene:Aqu1.215418

transcript:PAC:15713946 description:""
MRNSNEYLKYFLFRPKSSSSMDDIIELNHNFTLSGDINGPLLAAVISVEMIGGLIANSFV
LILTICHIKTWKQPSTIFLTNMLISNLLIVLFVMPFPITTCSNGKWMLGETEAEQVKYCY
FAGYLYWYSVLLITQSLVILSFDRFFYIVKSFMYERYMTGKRSLSIVAFSWLIASILNIT
PFIGLGSFGFINSYGSCGPLWEEEMGHVIYTFIIFIFYIGSIVTTTLWTYCYTRRFLQSE
RLRTEFISRIPQQCSVYISKEKRLIGLFGMMMVVYLTCYAPGLVAIVAVIFTPLPQPVYA
SVYVLFLLITVLSPLVQIIFRRDMREAVSKIIKCQRKEGASKCERQNSRSFMLTHLHT

>PAC:15713947 pep:novel
scaffold:Aqu1:Contig13214:64616:65617:1 gene:Aqu1.215419
transcript:PAC:15713947 description:""
MDDEQQDVLLRNYNFTLSADINGPLLAAVISIEMIAGLIANSFVLIITICHCSSGTWKKP
STLFLTNMLICNLFTVLLVMPLNIISLASRGWIFGVTVDQKLITCNLAGYIYWNSVLLIS
LSLMLLSIDRWIYIVQAMRYDSLVTPRRALLAIIITWSLAIVLNVTPFFGFGTYNFLESY
AGCSPIWEGHVGYVVYMLIIFVSIVGTIVVTSCWTFCYLFMYIRRRKERSSIGPGRKRSI
DQYVAARRKLIVLFGVLLFIHFISYLPGIITAGVVIFVSLPKEVYATSYILFLSITTLSP
LAQSCFREDIRSKLCKKKEEPPGTVDSSPFTDN

>PAC:15714975 pep:novel
scaffold:Aqu1:Contig13264:50611:51555:1 gene:Aqu1.216447
transcript:PAC:15714975 description:""
MEMDGNFTLSDRRDGIALAVVLTIEVILAAIANGAVLCITIYQRKHWRQPSTIFFTSLIL
AHLVMMLLYLPFTITALAAGEWSIGRTNEQRIGTCFFSIFLYMYTVLIVLMTLAAISFDR
FLFIVKPHLHKRFMKLWVAVSLAVAIWILSAVLNVIPLFSFEELTYDDTFGPCYPSYLNT
LAYALFIGIIFVTMIAIIVVTCTWTFCFTRKFLKDQSIIAGESVYSAKKRRLFGIFGSML
LVYIICFTPGGIHLALIPIIDTPFGLYATSLASYHFITIANPLVQSYFRPEIKSVFVSAY
HKMSPVSVTVNDTV

>PAC:15714984 pep:novel
scaffold:Aqu1:Contig13264:99897:100877:1 gene:Aqu1.216456
transcript:PAC:15714984 description:""
MERNYTFTGEFSPEAVAGVLSIEMILALIANVVVLVITFYQKKSFKHSSTIFFTSLILAH
LALTLLYLPFTITSLFAGEWIIGSTDDEKQGTCDFSAFMIITSAYIMYMTLSLISIDRFL
FIVKPHLHKRFMSPRVALVLVIIVWIVNSIFFSSGFIDESGIAYWYVAPLGICNALTISQ
AMAVIRFLVILILLGIIVVTSIWTFCFTRKFINNQSMIVGESVYASKKKRLFGIFGSMLL
VYGICFIPGAFLSSFLAILDAPGTLIISSLVFFILALVLSPVVQSYFRPEIKSVIVQICR
KMMRKPNSTASKNSAVTRDTNVDSVT

>PAC:15714985 pep:novel
scaffold:Aqu1:Contig13264:104307:104864:1 gene:Aqu1.216457
transcript:PAC:15714985 description:""
MERNYTFTGDFSPEAVAGVLSIEMILALIANGVVLVITFYQKKSFKHSSTVFFTSLILAH
LALTLLYLPFTITSFFAGEWIIGSTDNEKQGTCDFTAFMIITSAYIMYMTLSLISIDRFL
FIVKPHLHKRFMSPRVALVLVIIVWIVNSVFFSSGFIDGSGIAYQYVAPLGVCNALTISQ
AMAII

>PAC:15716328 pep:novel
scaffold:Aqu1:Contig13317:77929:78861:1 gene:Aqu1.217800
transcript:PAC:15716328 description:""
MEGNFTFTGEFSGPAVAAVFTVEMILALIANGVVLSITLYQWKSFKQSSIIFFTSLILAH
LVLNLLLNLPFTMIPLAAGEWIFGITNEEKRGTCHFAAWMNWSGSYVLSFTLAAISFDRFL
FIVKPHLHTRFMRPCVALTLTIAIWILSAVLGTLPFFDIGHYSYFDKLGLCSLVGVDIAA
FVVILVIIFLVVGTIFITSLWTFFFTFKAQSVIAGENVYTSKKKRLFGIFGSMLIIYGTG
YLIAALGFLLRIFIFLPYEFFITSHIVYSFVTVASPIIQSYFRPEIKSVLVSRCPLLFTC
VCCSCIHAVC

>PAC:15716337 pep:novel

scaffold:Aqu1:Contig13317:147680:148621:1 gene:Aqu1.217809
transcript:PAC:15716337 description:""
MEGNFTFTGKFSGQAVAAVFTVEMILALIANGVVLSITLYQKKSWKQSSTIFFTSLILAH
LVLNLLHLPFAVIALAAGEWIFGSTDEEKRGTCTFASYLYWYIVDVISMTLAAISFDRFL
FIVKPHLHKWFMRPWVALTLTIAIWILCAVLGTLPFINIGHYSYDVELGFCSIVGVDIAA
FVLILVIIFLVVGTIFVTSLWTFCVTKSYFKDQSVIAGESVYASKKKRLFGIFGSMLIVY
GTAYLIAALGFLLQIFIFLPYEFYVTNYIVFCFVTIASPIIQSYFRPEIKSVLVSRCPLL
FTCVCCSCVHSIY

>PAC:15716338 pep:novel
scaffold:Aqu1:Contig13317:148885:149805:1 gene:Aqu1.217810
transcript:PAC:15716338 description:""
MEGNFTFTGEFSGPAVAAVLTVEMILALIANGVVLSITLYQRKSLKQSSTIFFTSLILAN
LVLNLLCLPFNIIALAAGEWIFGSTDEEKTATCIFAAFVYSYTIPVIFNTLAAISFDRFL
FIVKPHLHKRFMRPWVALTLTIAIWILSAVYSFAPFYGLNEYVYNEQYLLCYQHSNSIIS
VIMFIVFLPCVTTAIIVTSLWTFCFARSFLKDQSVIAGESVYASKKKRLFGIFGSMLLIY
GICVVPSLLLLAFQQFIDVPPELFICIRVGFFFITIASPIIQSYFRPEIKSALASCCPLL
FYCVCC

>PAC:15716428 pep:novel
scaffold:Aqu1:Contig13321:92542:93519:-1 gene:Aqu1.217900
transcript:PAC:15716428 description:""
MKRNYTFTGDFSPEAVAGVLSIEMILALIANGVVLVITIYQKKSWKQSSTIFFTSLIMGN
LLMTMSYLPFSIAALAAREWIIGSTDEEKQGTCDFTAFVMIYCGYVTLMTLSLISIDRFL
FIVKPHLHKRFMSPRVALVLVIIVWIVNPLFFSSGFINGSGIVFQYINNVGVCYAYSDSP
IAAAFRLSLIIILLFIIVVTSVWTFCFTRKFINNQSMIVGESVYASKKKRLFGIFGSMLL
VYGICYIPGTFFNSSIAKANAPDKFNISALVLFLLALIVSPVVQSYFRPEINSAIVNIIC
RKMRKKRNRTTPSNASKTNTNSFDL

>PAC:15716430 pep:novel
scaffold:Aqu1:Contig13321:95007:95984:-1 gene:Aqu1.217902
transcript:PAC:15716430 description:""
MERNYTFTGDFSPEAIAGVLSIEMILALIANGVVLVITIHQRKSWKQSSTIFFTSLVLGN
LVMTTLYLPFTITSLAAGEWIIGSTDEEKQGSCDFTAFVIIYCGYVMLMTLSLISIDRFL
FIVKPHLHKRFMSPRVALVLVIIVWIVNAAFYSAGFINGSGIEFQYINNIGICYAYATSP
IAVVFRLSLIVILLSIILITSVWTFCFTRKFINNQSMIVGEGVYASKKKRLFGIFGSMLL
VYGICFIPGTFFSSSLAKVNAPDKLNISALVLFLLALILSPVVQSYFRPEINSVIVNIIC
RKIMKKRNRTTPSNASKTNTNSFDL

>PAC:15716431 pep:novel
scaffold:Aqu1:Contig13321:96385:97362:-1 gene:Aqu1.217903
transcript:PAC:15716431 description:""
MERNYTFTGDFSPEAVAGVLSIEMILALIANGVVLVITIYQRKSWKQSSTIFFTSLILGN
LVMTMLYLPFSIAALAARE WIIGSTDEEKQGTCGLAAFVMIYCGYVMLLTLSLISIDRFL
FIVKPHLHKRFMSPRVALVLVIIVWIVTAVLNSSGFINGSGIEFQYIDNVGVCYAYPTSL
IAAVFHLLLIVILLCIIVVTSVWTFCFTRKFINNQSMIVGESVYASKKKRLFGIFGSMLL
VYGICYIPGTFFTSSIAKANAPDKFNISALVLFLLALILSPIVQSYFRPEINSVIVNIIC
RKMRKKLDPITSSNAIKTNTNSFDL

>PAC:15716432 pep:novel
scaffold:Aqu1:Contig13321:99445:100431:-1 gene:Aqu1.217904
transcript:PAC:15716432 description:""
MERNYTFTGDFSPEAVAAVLSIEMILALIANGVVLVITIYQRKSWKQSSTILFTSLILAH
LLIILVLPFCIAALAAGEWIIGSTDEERKGTCDVSAYILLYSGNIMYMTLSLISIDRFLF
IVKPHLHKRFMSPRVALVLVIIVWIATALFFSSGFINGSGTVFKYINNVGACYGYSASPI
MAAIRFSIITILLSIIIITSVWTFCFTRKFINNQSMIVGESVYASKKKRLFGIFGSMLLV
YGICFAPGTFLSSFFAIIDAPDKLNISALVLYLLAFILSPVIQSYFRPEINSVIVNIIYH

KMMKKLNQSYYQTTSSNTSKTNTNSLDL

>PAC:15716433 pep:novel
scaffold:Aqu1:Contig13321:101088:102068:-1 gene:Aqu1.217905
transcript:PAC:15716433 description:""
MERNYTFTGDFSPQAVAGVLSIEMILALIANGVVLVITIYQRKSWKQSSTIFFTSLILAH
LFMILYLPFTITALAAGEWIIGSTDEEKEGTCDFTGFLIIYCVYVMLMILSLISIDRFLF
IVKPHLHKRFMSPRVALVLVIIVWIVTAVLFSSGFINGSGIVYQYVENLGGCYVYTTGPI
ASVIRYLFASLILSIIIITSIWTFCFTRKFINNQSMIVGESVYASKKKRLFGIFGSMILV
YGICFAPSVFLSSFMTTIDLPDELHVFSLVLLFLVVTLSPVVQSYFRPEIKSVIVDVICH
KMMRKPNSSDPGVTQDTNINYNTIGP

>PAC:15716434 pep:novel
scaffold:Aqu1:Contig13321:103086:104042:-1 gene:Aqu1.217906
transcript:PAC:15716434 description:""
MERNYTFTGDFSPEAVAAVFSIEIILALIANGVVLVISIHQRKSWKESSTIFFTSLILAH
LLMLLVIPFSITSLAAGEWIIGSTDEEKEGSCGFTAYITYCSVYNTYVTLSLISIDRFLF
IVKPHLHKRFMSPRVALVLVIIVWIVTAVLLSSGFIDGSGLTYQYIDNIGGCYVGITSPI
ASIFRSSSASIILSIILITSVWTFCFTRKFINNQSMIVGESVYATKKKRLFGIFGSMLLV
YGMCFIPATFLQSFLTIIDAPDKLIVSSLVLFLLGIILSPVVQSYFRPEINRIIVDVICH
KMMHTPTPTDPNTSNNLA

>PAC:15716436 pep:novel
scaffold:Aqu1:Contig13321:108135:109154:-1 gene:Aqu1.217908
transcript:PAC:15716436 description:""
MERNYTFTGDFSPEAVAGVLSIEMILALIANGVVLVITIYQRKSWKQSSTIFFTSLILAH
LVLTLYLPFSIAALAAHKWIIGSTDEEKKGTCDFNGFVILFSAYVMFMTLSLISIDRFLF
IVKPHLHKGFMSPRVALVLVIIVWIVNAVFFSSGFINGSGMVYQYIDSQGLCYANTTSPI
MAATRFSMAMILLFIIIITSVWTFCFTRKFINNQSMIVGESVYATKKKRLFGIFGSMILV
YXXXXIFGSMILVYGICFTPGTFLSSFLAIIDVPDQLTISSVILFFLAIILSPVVQSYFR
PEINSVIVNIICHKIMHKPDHNLTVKRDASCTNIASFDF

>PAC:15716437 pep:novel
scaffold:Aqu1:Contig13321:111574:112554:-1 gene:Aqu1.217909
transcript:PAC:15716437 description:""
MERNYTFTGDFSPEAVAGVLSIEMILALIANGVVLVITIYQRKSWKQSSTIFFTSLILAH
LVLTLYLPFSIATLAAGEWIIGSTDEEKKGTCNFNGFIVLFSAYVMFMTLSLISIDRFLF
IVKPHLHKRFMSPRVALVLVIIVWIVNAVFFSSGFINGSGMVYQYIDSQGLCFANTTSPI
MAATRFSMAMILLCIIVVTSVCTFCFTRKFINNQSMIVGESVYASKKKRLFGIFGSMLLI
YGICFAPGTFLSSFLAIIDPPNQLIISAVILFFLAIILSPVVQSYFRPEINSVIVNIICH
KIMDKPDHNLTVKHDSSCTNTVSFDS

>PAC:15716438 pep:novel
scaffold:Aqu1:Contig13321:112825:114269:-1 gene:Aqu1.217910
transcript:PAC:15716438 description:""
MERNYTFTGDFSPEAVAGVLSIEMILALIANGVVLVITIYQRKSWKQSSTIFFTSLILAH
LVLTLYLPFSIAALAAREWIIGSTDEEKQGTCDFIGFIVQFSAYVMYMTLSLISIDRFLF
IVKPHLHKRFMSPRVALVLVIIVWIDLSMDLFGRFVSLSMIVGESVYASKKKRLFGIFGS
MILVYGICFTPATFLAAFLVIIGPPNQLIISALILFFLAIILSPVVQSYFRPEINSVIHL
KPKSILEMIL

>PAC:15716439 pep:novel
scaffold:Aqu1:Contig13321:117887:118867:-1 gene:Aqu1.217911
transcript:PAC:15716439 description:""
MERNYTFTGDFSPEAVAAVLSIEMILALIANGVVLVITIYQRKSWKQSSTIFFTSLILAH
LTLTLYLPFSIAALAAREWIIGDTDEEKKATCDFNGFMILYCAYNMLMTLSLISIDRFLF
IVKPHLHKRFMSPRIALVLVNIVWIVNAAFFSSGFINGSGMVYQYIDNVGLCYAFTTSPI

MAGTRFSMIMILLCIIIITSIWTFCFTRKFINNQSMIVGESVYASKKKRLFGIFGSMILV
YGICFAPATFLSSFLAIIDSPNQLIISAVILFFLAIILSPVVQSYFRPEINSVIVNIICH
KMKHKPGHNLTVKRDASCTNTASFDF

>PAC:15716440 pep:novel
scaffold:Aqu1:Contig13321:119695:121318:-1 gene:Aqu1.217912
transcript:PAC:15716440 description:""
MERNYTFTGDFSPEAVAAIFSIEMILALIANGVVLVITIYQRKSWKQSSTIFFTSLILAH
LLIILVLPFSITALAAGEWIIGSTDEERKGTCDVSAYILLYSGNIMYITLSLISIDRFLF
IVKPHLHKRFMSPRVALVLVIIVWIATALFFSSGFINGSGTVFEYINDIGACYAPVVQSY
FRPEINSVIVNIICRKMRKKLNRTTPTAGEWIIGDTDKEKKGTCGFNGFMILYCAYNMLM
TLSLISIDRFLFIVKPHLHKRFMSPRVALVLVIIVLIVTAVLLSSGFINGSGIVYQYIDS
QGLCYANTTSPIMAATCFSMTMILLSIIVVTSIWTFCFTRKFIKIKKYKQQQVKTYKDYR
NTTTVQYY

>PAC:15716509 pep:novel
scaffold:Aqu1:Contig13324:109378:110541:1 gene:Aqu1.217981
transcript:PAC:15716509 description:""
MNVTEVPGFQKGLFVFFWLISIGLNTLCSALLMIAVHKRKSWANILLLSIALTGIAAVLL
GMPLSIVSLFINDLLIDEGALCQYQVIVFNWYILMSFFLVVFISIDQYLAVLYPFMYNTK
ILRHPKRSLKIIRILLFFVTSGTLSYSIIGSFLSTIAVMTPSNICYYDFLAVDTPSKIFF
IVNTIAMGLMILVILTCTILLAIKMYRMFLKAEDPKDTFQVQKANFGHFLLVIIIVFMAA
ASLFLVVHILYVAGYSVSVYLHYLSLCLVLSNSLLFPLIFILIRASVRQILKEIWKKLLY
CREDKETTGKRQDKAVQLKQLKKSY

>PAC:15716748 pep:novel scaffold:Aqu1:Contig13334:5516:6466:-1
gene:Aqu1.218220 transcript:PAC:15716748 description:""
MSLFGNDSDGFGFTGNVNAPTYAAALAIEAVAGLVANFTVLAITLYQRNSFKQSSTIFFT
SLLLANLIMVIYILITSITIGTEEWIVGSTHEEKTATCAFFAYMSWNSYMTMSMTIAAIS
FDRFLFIVKPHLHKSFMKPQVALFVTIGIWLLSGVINTTPFYGLGEYGYASYFGLCSSLL
DRKGSNIAYLFIHAVIFMSIYASIFITSLWTFCFTRRFMQSQSEIARNSVYVSKRKRLFG
IFGTMLLAYAIALSPTYINSIVGVFYDIPLEVFVISCIVYNSITIINPIIQSYFRPEIKI
VYKTIYTIIKTKIRRQ

>PAC:15716749 pep:novel scaffold:Aqu1:Contig13334:6674:7678:-1
gene:Aqu1.218221 transcript:PAC:15716749 description:""
MSLLELNSTTFTATGEVNGPAFAAAFAIEGLIGLIANITVLAITLYQRKSWKQSSTVFFT
SFLLANIITVMYMLVSSISIGAGEWIIGSTFEEKRATCTFTAYVIWLDSMALTMTIAAIS
FDRFLFIVKPHRHKYLMKSWIALIVTISLWVLSAVVNIAPFYSHDEYKAGIYDSQIGICL
KNFIILVTSLWTFCFTCRFMQTQSEMPGNNSVYVSKKWRLFGIFGTMLLAYIIAVIPAYI
FGVISIFYTLPPAVNVTAALAYGSIVISNPIIQSYFRPDIKIVLTAIIKKIKLKVTRRYQ
NTTQTDANMELE

>PAC:15716877 pep:novel scaffold:Aqu1:Contig13338:4914:5852:1
gene:Aqu1.218349 transcript:PAC:15716877 description:""
MDIHGLNYTLGEDVNGPLLAAALAVEMIAALIVNTFVLVATFSQCKSLKLPSTILFTSLI
MIHYVIALIYIPSWLISAAYGEWIFGSTIQVKEATCKFAGFILNYTLQVKSFTLAAISVD
RWLFIVKPIFYKQYMKAKLAVVLAVIIWIGSCLSVATPFFGIGNYFFTEFGSCEPQFLGE
LGYSILMLIFVLTALCIIIVTSIWTCCFTRRFIREHAQLADESAYVSKNRRIIGIFGAML
VAYGVCYTPGIIVIIASLFCDVNAAAFAAVLVFFLTVIIINPIIQSVFRPDVKKVIVKLC
AMCRKFHHSPEA

>PAC:15717007 pep:novel
scaffold:Aqu1:Contig13341:69084:70064:1 gene:Aqu1.218479
transcript:PAC:15717007 description:""
MSFEHNFTTTGNVNGPALSAVLALETVTAFIANTVVLSITLYQRKSWKQSSTIFFTSLIL
SHLVMVFLYLPLCVIAVAAEEWIFGSTFEQQIATCSFAAYVIWYCVLVIQMTLAIISFDR

FLFIVKPRIHKRLMRPWTALAFTIGIWILAAILNSGPFVGLGEYEYDDTYGSCVPLWQGN
NGYVLYMLIISMISITIILITSIWTFCFTRRFINDQSQVSGDSVYVSRKKRLFGIFGSML
LVYGICFLPSIISGGITTVVILPDPVYAVNMVTFQFITVASPLVQSYFRPDIKAAIISFF
ALFAPALKKWQGLTSRSHETSTSLPA

>PAC:15717374 pep:novel
scaffold:Aqu1:Contig13350:136964:139713:-1 gene:Aqu1.218846
transcript:PAC:15717374 description:""
MDEQRLEYLEFYNQSCNVSTINSSSSFGPPIAVIRIIGSILSILGSTSIIIAVFLTGKLS
NMEIHPLFSLAVGDFILSCLWLFGGSLWLDTYQDRYSDIHPGLGLCYILAISTTIVNMVT
SFLTIVYSLHAFIVMRQLSKSRGHISRGRTCKQHLITAFVYLVAWSLPLLLVLPVTESMI
GLDIANNACWCFIDFFNTRPGGGPNTEISDSDDLATIIAVYSGVTFVVTAIAIIALYAGT
LHMARKVLKHQKTLNSEINSSKVIKKLAGRAAAFIIIYLICGLPFLAGAIDVWKENTPLH
LLEKPDQTILFWQAIFGPLQGFLNAIVYGWSRPEFRRLFVTPCKKLRRRMRVKDTHK

>PAC:15718112 pep:novel
scaffold:Aqu1:Contig13373:75281:76844:1 gene:Aqu1.219584
transcript:PAC:15718112 description:""
MFLPHYDYDHGNNLSADQGDVSPTQDIGFIIALNISVGIVCLLSILGSTSIIFSYILYKE
LRTTGRFILLNLSVADLIVALANLLGAAMSPRFRNVNDPSDINDSSSLLALSCKIDAFFG
LFATDSSILWTIVLLSYLYITLHCFRPSRSCFKVSMTICMIICWGLPLAISVWFISKNFF
GFQPGFSPGFCTFTGDNDGKDIYRPIVGYEMFLYPSIIFLTTISIAFFCHAKCKKKPVEN
YHYIEHHLNSIKAAERKLLFVPIVFIFIRIWGLITDVTTFYLNDNQIATLRNSKISPALI
FMAGIGDSSQGFVNGIFFCLLTPPVRQRLLLLLFKCGRICRCYCCSRYETLVNDAYITPT
TSRSLTISYTNSTASRGGGVEGEGDGDNSTLSRSLGTISADISLYGSNEIN

>PAC:15718483 pep:novel
scaffold:Aqu1:Contig13383:203940:204863:-1 gene:Aqu1.219955
transcript:PAC:15718483 description:""
MSLNSTDFVLMGDLNSTTYAAVLGIEGVIGIIVNVAVLLMTLYQRKSWNQPSTIFFTSLL
LSNLIIALWYFMSSIAVGAEEWIFGNTFKEKNASCLILGYMFWNGGMIIVATLAALSFDR
FLFVVKPYLHKRFMRPRVALILIIGVWLLCSLINTTPFYGFGVYRYQSRSGICSPSYEAS
GFFYVLYLVVVYSIIYFVIAFTSIWTFCFTRRFIQNQVEIVGSSVYESRRKRLFGIFGYM
LLSYVIALLPSYITGIISMFYQLPANVHLGTVVANGSFIISNPIIQSYFRPEIVTVIKNL
WNKIRNT

>PAC:15718484 pep:novel
scaffold:Aqu1:Contig13383:206185:207896:-1 gene:Aqu1.219956
transcript:PAC:15718484 description:""
MSFNSTDFVLTGDINSPTYAAALGIEGVIGIIANVAVLLMTLYQRKSWNQSSTIFFTSLL
LSNLIIALWFLMSSIAVGAEEWIFGNTFEEKNATCLVVAYLIWNCSIITMMTLAAISFDR
FLFIVKPHLHKRFMRPRVALILIIGVWLLCSLINTTPFYGFGVFMQDQAEIVGNNVYQSR
RKRLFGIFGYMLISYLIALLPSYIDGAISMFYYLPACVRLGFVVTYGSFIISNPIIQSYF
RPEIVIIIKNLWNKIDDHGLVNFHPLDVRVDESLETLLLYIDNAIQYGEDIDVKVPKLED
DDNEDS

>PAC:15718485 pep:novel
scaffold:Aqu1:Contig13383:210830:211729:-1 gene:Aqu1.219957
transcript:PAC:15718485 description:""
MMDIMSFNSTDFVLTGDINSPTYAAVLGIEAIIGMIANVAVLLMTLYQKKSWNQPSTIFF
TSLLLSNLIIALWYLMSSIAVGAEEWIFGSTFEQRNATCLFNAYLLWSGRMIATMTLATV
SFDRFLFVAKPYSYKRFMRPRVALILVIGVWVMSSLINTIPFSGFGVYGYFSLNGICTLL
YAYSGLYLIILFVVYAIIFVIIAVTSFWTFCFTRRFMQDQAEIAGGSVYQSRKKRLFGIF
GYMLLSYVIALLPSYIIGTLNMFYHLPAYVHLGVVVAYGSFITSNPIXXXXKSYHSIIF

>PAC:15719539 pep:novel
scaffold:Aqu1:Contig13412:154418:156225:1 gene:Aqu1.221011

transcript:PAC:15719539 description:""
MIDGQLNDKNNKLNKTSDLHLFTTSGDNIKHQWDLSIPSFSTACNLLRVHPYYIEQYKKT
FQTEFSKDKCSFFNPEAVAGVLSIEMILALIANAAGEWIIGNTDEEKQGTCGFTAYIILY
CAYVMFMTLCLISIDRFLFIVKPHLHKRFMSPRVALVLVIIVWIVNALFFSSGFIDGSGV
LFQYLDNSGACYAFTTSPFTAVIRFSLASIILCIIVVTSVWTFCFTLGESVYASKKKRLF
GIFGSMLLVYGICFTPTFVNAPDKLIISAFILFFLAIILSPVVQSYFRPEINSVFVDIVC
HKIMNKSNQTMSSSRQFTIV

>PAC:15721359 pep:known
scaffold:Aqu1:Contig13450:70950:73394:-1 gene:Aqu1.222831
transcript:PAC:15721359 description:""
MTSSELLSLSSTSTEISSPTCTSIAVTVTPTVTQTLVCIGETVSSTSIAVSTTTSTPTIT
STSTTSTTPTPTEPPFDCNTCYDSEECNWCLSNDCEISGCEKYTTCCDDFKKRRRRDTQA
NETIILASSSIMDSLDCSSVQVTITPSVTVSLTCFQASTFPSPSPSITECENEPCSNLIC
SPVVQSAVTSGSVTSTVVIPSSSSSLPSPSSTLRSSSSSSSTSDQNSAFVGTPTPTCTSLN
VAQGSFFEEMKDFVSCSPGNDPFNPCTDLIDNNLLRAGMWLVIILSIGGNIVVLSATLLY
FISRYLKNHKKPHLMYFLYINLAMADLFMGIYLLTIAVVDLDTIGDYSRHAIEWQTSAGC
RFAGFCAIFSSLLSIYTLLVITVERVYTIKFALQHKRFHKQTVTVSILVGWVLTITLCIL
PMVGLSSYERVGICLPFEARETADQAYIVLILVLTGIASFAIMFCYVLLFYLVVCNNRSI
TKTLSGREELKLALRMSLLVMTDFACWAPIALFGLTAVFQKPLINVTDSKILMVFVFPLN
SCLNPILYSFSTRKFRSIVFSAFDRCNVCKKCCAKKQIKQNSSNEEYSTGKSNSRRSSED
VTVNGPFLNQRQAHRRGTELSLISGFTTSSRRGSTLSGGSDEETPPMHSLIHTGRPERSS
QSSLGSDNSYLSEQGESVINTSTSSLGSLKDRRFSLASLSGHVSQLTALPEEKEEDLPCE
KHRESTHSSSDSFNENIRLEYKPVLDDRKLPTNPSFIPLNEGTTTIYINECIDDDDDDDD
DAKGHQRSEEEQTIVTMYNDAEGIEIQETELTFT

>PAC:15721762 pep:known
scaffold:Aqu1:Contig13456:263938:287969:-1 gene:Aqu1.223234
transcript:PAC:15721762 description:""
MASTRYLPGATSVEIPLRDTDEVIELDIDQLPEGDEVITILRDERAPLHTWVTLALHYYK
QDKWSEFEKLLQTSRTDANISYDDHEKDQMMALDSLAAHYVQKAKKERDKETKKEYFSKA
TLLYSTADKIMMYDSRHLLGRAYLCLLEGDKMSQSEAQFNFVLQQSPDNLPALLGKACVS
FNRKDYKGALVCYKKALRSNPNCSGTVRLGMGHCFMKLGNIDKAQCVGALVGLAILELNN
QQHDSIKRGVELLSKAYTIDSVNPMVLNHLANHFFFKKDYVKVQHLALHAFHGTEVETMR
SESCYQLARAFHVQGDYDQAFQYYYQATQFAAPGFVLPHFGLGQMYLARQDSENAAQCFE
KVLASQPGNYETLKILGSLYANSPSSEKRATAVTHLKKVTEEFPDDVEAWIELGGILEAT
DTEGSLKAYEKASQLLTETVGTDIPPEILNNIGCLHFKLGQYNEAQSHYDQSLDRCTQEC
MQDEEYYNSLMVTVRYNMARLHEALCEFEKAETLYKEILKEHPRYIDCYLRLGCIARDRQ
QIYEASDWFKEALQKNQDHADAWVLMGNLHLAKQEWGPGQKKFERILQNPKTKGDTYSLL
SLGNVWLASIHQPHRDKTKDKRHLDRALSYYKDVLHKDSHNLYAANGIGAVLGHKGFYRE
ARDVFAQVREATAELPDVWLNLAHVYIEQKQYISAIQMYENCLGKFYNFHNTEVLLYLAR
AYFKAGRILDCKTTLIKARHIAPHDSLLLFNLALVQRWSASSTLKNLQSTLADVLSAVRE
LEMAQRNFVFLSREGDRLKFDLQFASHEAKRCADLLSQAQHHVARARKSEDEERELREKQ
EKEMETLRQKQIEQEEKLAKERERQLQLQEQARQEFIQKTQNLLHFTREASPPPKPSRKK
RGTADVQSDDPENEASASGPQRKRRRRNNEEDTGDTAASGGKGRKKKRFISQAFVSSSED
ESGGEEKKTEETAVEEREGESHEGEEEVGERSLQSEDSSEESTSGSESESGSGSNDEVRQ
EEEGKEEDEEEAKGEEPKEGSSPEPQEDTTAEQEVEEKVTQHNVSNRRRIESDDDETLAI
DCRRLELTAVPEFLQTFSNYSTFKQISMYLDYNNISGIGTNDLSLMKNLTRLTITHNQLS
YIEDNAFSYQDFFNILDLSFNLLTSESFANDSFNSTGSRVATLKITDNLLTHPPRALAKK
NRHMSMSTLFLDNNPMGPVLKSDAFEGYAQDIRELFLNNNDFNEIDVKFLEGAHISDKLY
LRNAGIREIRPRTFWKHTSLKHLYLDNNDLSRQESRNSFVKMDLKTLNLKGNFITRIFNE
TFRDLKSIRELILDSNKINRIDAQPFRKMTLLRKLYLGNNEIDDSNPYFLGDLQSLTSLD
ISRNRLTEFPDVSGLPILTTVLASYNNIKELKEATFNTSEYLKKLQLSDNRNLEVIDRNA
FKRNNQIEMVDVSFSGLKRLPSFETFPQLYELRLEHSKLTELPKDLCTIAPNLRLLYAYD
NYLESIPDLSNCTKLIHIELYYNRIKAIPEFAFKGLSDLQTLKLYGNRITTVHRNAFRGL
NRLYELDLSHNQITYLPHGLFFNLTNLRKLELQFNAITNLPDNIFSDLNHLELLYINDNA
IRTVGSVLFHGNMTWLQNLSVSNNPEMDSFPIPTDGFPFLHSLGMSNLPLIFNVPTVINI

PRIQTIDFTYSYHCCLFKDYAPPYRALTLEESENTGDPDVLFTFQNTPAEEVTLAPEVSD
TLLFHGSDPFTHDNAGDISPQQFIRELLEYQRRFNVTLVSTQNGQVIVVDNSNHSIVARL
KDEESRYLRTILPSFFVNREVYCTPPPSSLTPCDNLLDPQPLPVLVWVIWFTAVIANLAV
LFVMIVSKEKLEVPHFFICNLAFADFLLGVYLAFLGAVDIRTRGKGFYKSALTWQTGPGC
QTAGFIAIFSAELSVFILTFLTLERLHTIAYSFKSGRLRLRNATIIVFICWILAGILAAL
PLFDVNTYSEVAVCLPFRLSNIRDKLFIALILTVNLTAFFIILSSYLHILRLFCRSRASC
TEQGNKREKIVISFKMGMLVLTNLICWLPLAVVGYAAIVDQHIINFTVAKFFIILIFPIN
ACLNPFIYSIFTKQFLSRVRGLCKRKDTMQHVSNNNSFYRLSFRRNSIASAASDTCSSTL
RTNSPRFDIDLDMLSRRQSRRSFSVQLASPPLPQPIVGLASPAPYMGRRYSSPAIFGMES
GLPEAQGLEGRGEIPRSHSKCLSIVQEESDESDTEEAAASHSPRRRRRSSDPELLNSALP
GGLKDLLNARSNKVSDERRDSGLVPSFEGNSPLHYQSVSPPAQSSTLIRTSGEGHSRDDA
TSTNYRIPSLSSPAAASRVHFKEYIPRRSSPLSTHSIRIINPHTAKQSKHNVKESQV

>PAC:15722199 pep:novel
scaffold:Aqu1:Contig13465:46025:47249:-1 gene:Aqu1.223671
transcript:PAC:15722199 description:""
MSNNSTDYSYPCGLPPDFPPSFGIPPAINYIQAGIALVTGIIGLLLNIFILFIIIKYRSL
HQRLMFIAIQITIVEIAYSLLVPPAIVVSGIARDWLLGEAMCNILGIVNDGFAYFRFMMT
FILTLDRFVSVFLPFFYEKHSKRIIYSLLSLVYFTTFFRVLLPVKGIMGCYIYAYRLRRA
TRRIVPENLSAESQRGNETKILKESFQFFTNITHGAFFAVVMLVGRTSFNSIPVVDAIAI
MKDKEFREAAKKLILSIRERLDEFHSLSVEEWKPKH

>PAC:15722200 pep:novel
scaffold:Aqu1:Contig13465:47402:48655:1 gene:Aqu1.223672
transcript:PAC:15722200 description:""
MNNSSLESSYACGLPEDFPPSLNIPPAINYIQAGIALVTGIIGLLLNIFILFIIIKYRSL
RQRLMYIAIQISIVEIAYSLLVPPAIFISGIAREWLLGEAMCNILGIVNDGFAYFRFMMT
FILTLDRFISIFAPFFYERNSKKILFGLHGLVYCTTFFRVLLPINGIMSCYVYVSTNKIC
TAFSGCSLGCYYFVFVSIILIIVVGALLPLSMYVILFIKAYRVKKATKKMVPAPSTPSEE
MKSCTSQKGSVVYPELASAPSAFDDDLTDQKTKRQIRPSVTSSKSRHLSLSAENKKSLQV
TITMFILLLSVIGCTSPAFVLYMVQFLSLHDQKTFFILIMLLGRTSFNSIPVVDAIAIMR
DQQFRKSIKLCFRR

>PAC:15722205 pep:novel
scaffold:Aqu1:Contig13465:58133:59373:1 gene:Aqu1.223677
transcript:PAC:15722205 description:""
MDANYTCGLPPDFPPSLNIPPAISYVQASIALVTGIVGLLLNAFILIIIIKYRSLHQRLM
YIAIQIIVVEIAYSLLVPPAIFVSGIARDWLLGEAMCNILGIINDGFAYFRFMMTFILTL
DRFVSVFFPFVYERNSKKILIILLIMVYFTTFLRVILPLKGVMGCYTYVPTNKICTAYSD
CSTGCYYFVLASVVTIVLIGAIIPFCMYMILFYKAYKVRKISSQAVTTHSPSPSFSSIAT
VQVNMSTIKGSLPRGRESSICEHDLEKSRKSISGDENFQVTLTMFILLMSVIGCTSPAFV
LYIVQFISLHNNSAFFIVIMLLGRTSFNSIPIFDAIAIMRDKQFRSSAKTFLGTLRKCI

>PAC:15722301 pep:novel
scaffold:Aqu1:Contig13466:127368:127973:1 gene:Aqu1.223773
transcript:PAC:15722301 description:""
MESNSTFSEEFSGPAVAAVFTVEMILALIANGVVLSITLYQRKSLKQSSTIFFTSLILAH
LVLNLLYLPLFIIGLAAGEWIFGSTDEEKRGTCYFGAFIFWWMVLVISLTLAAISFDRFL
FIVKPHLHKRFMRPWVALTLTIAIWILSAVLSIVHLFGFRLYIDICLSSTADIGFVVYII
LLSAIVLGIIFVTSFWTFFFH

>PAC:15722303 pep:novel
scaffold:Aqu1:Contig13466:143707:144618:1 gene:Aqu1.223775
transcript:PAC:15722303 description:""
MDGNLTFTGEFSGPAVAAVLTVEMILALIANGVVLSITLCRRKSLKQSSTIFFTSLILAH
LVLNLLYLPFRIIALAAGEWIFGSTDEEKRGTCRFFDVFIYIWMVLVISMTLAAISFDRF
LFIVKPHLHKRFMRPWVALTLTVAIWILSAVLSSIYSFGFQLKFDSHYNCSVTISISLGV

SAIILAFIFNGIIFITSLWTFCFTRRFFKDQSVIAGESVYASKEKRLFGIFGCMLLVYAI
CFVPGVLYFLLLLFINVPLWLNIVVIVCFLFITVASPLIQSYFRPDIKSVLVLYCPFHFC
VCC

>PAC:15722304 pep:novel
scaffold:Aqu1:Contig13466:158017:158931:1 gene:Aqu1.223776
transcript:PAC:15722304 description:""
MNFNMESNSTFSDQFSGPALAGILIVEMILALIANGVVLSITLYQRKSLKQPSTMFFTSL
ILAHLVMNLLNLPFIIIALAAGEWIFGSTDEEKRGTCYFKDFIFWWMVVVMLTTLAAISF
DRFLFIVKPHLHKQFMRPWVALTLTIAIWILSAVLSIVHLFGFHSYTDICLPSTTGIGFS
VYAILLSAIVLGIIFVTSLWTFCFTRRFFKDQSVIAGESVYISKKKRLFGIFGSMLLIYG
ICFVPAVLYFLLRLFIDVPIWFIIVAIICFHFITVINPVMQSYFRPEIKSVIFTCCPLHI
CVCC

>PAC:15722305 pep:novel
scaffold:Aqu1:Contig13466:174002:175763:-1 gene:Aqu1.223777
transcript:PAC:15722305 description:""
MVYSRETEGMESNYTYTSDFSGPAVAAVLTVEMILALIANGVVLSITLYQRKSLKQPSTI
FFTSLILAHLVLNLLYFPFFIIAFAAEKWIFGSTDEEKKGTCYFVDFTFWWILLVISITL
AAISFDRFLFIVKPHLHKRFMRAWVALTLTIAIWILSAVLITILGIIFVTSLWTFCFTRR
FFKDQSVIAGESVYASKEKRLFGIFGSMLLIITGCIKCILNLIA

>PAC:15722306 pep:novel
scaffold:Aqu1:Contig13466:176289:177188:-1 gene:Aqu1.223778
transcript:PAC:15722306 description:""
MERNFTFTGNFSGPAVAAVLTVEMILALIANGVVLSITLYQKKSWKQSSTIFFTSLILAH
LVLNLLYLPFIIIGLAAEEWIFGSTDEEKRGTCYFNDFIYWWMVIVISITLAAISFDRFL
FIVKPHLHKQFMRPWVALILTIAIWILSAVLSSVHVFGFHLYIDICFSYSDNIGFRVYFV
LLSAIVLMIIFITSLWTFCFTRRFFKDQSVIAGESVYASKKKRLFGIFGAMLLIYGICFV
PGVLYFLLLLFIDVPIWFIIVAIICFLFITIASPVIQSYFRPEIKSVLVSCFLLCNCKC

>PAC:15722307 pep:novel
scaffold:Aqu1:Contig13466:179848:181571:1 gene:Aqu1.223779
transcript:PAC:15722307 description:""
MEDNYTLTDQFSGPAVAAVLTVEMILALIANGVVLSITLYQRKSLKQPSTVFFTSLILAH
LVLNLLYLPFYIISLSAGEWIFNGSDEEKRGTCYFALYIFWWMVLVISTTLAAISFDRFL
FIVKPHLHNRYMKPWVALTLTIAIWILSAVYTTFYIFGFQFDHHQFDSQYCSLPSSSSRG
FFGLFAALIILGIIFVFSIWTFCFTRRFFRDQSVIAGESAYASKKKRLFGIFGSMLLIYG
ICLVPAVVYSLLYLFIDVPIWFIIIGTISFLFFTIANPVIQSYFRPEIMGVLSWLDNKHT
VFGRFFMDVAQKISEVKTDPRSNNPYDEIKVI

>PAC:15722410 pep:novel
scaffold:Aqu1:Contig13467:295897:296859:1 gene:Aqu1.223882
transcript:PAC:15722410 description:""
MSFNDSFIAMGTINGPAVATVLAIEGLIGFIANIIVLGITIYRKKSWKQSSTIIFTSIIL
AHLIMIIMFLPLFVIAIGAEEWIFGSDDMQKRQMCLFLNFVEWTMKLLIQIMIAEVSFDR
FLFLYKPHHHKKYMRWWVTLTLTTVLWIVAIILNVLPLFVDDNDFSYIFTYRPCIPRRQV
SSYYFLVDLASYILIYVFIFITSVWTLCFTYRFIHNLSEVAGESVYASKKKRLFGIFGSM
LLAYFIGFAPFTIAIIIRSFYGVPIEVFATTEVTFQMTHIATPLIQSYFRPEITNTLISF
KAFLRQKIHSARYDVRNPEI

>PAC:15722868 pep:novel
scaffold:Aqu1:Contig13474:41881:42879:-1 gene:Aqu1.224340
transcript:PAC:15722868 description:""
MERNYTFTGDFSPEAVAAVLSIEMILALIANGVVLVITIYQRKSWKQSSTIFFTSLILAH
LVLTLYLPFTISALAAREWIIGSTDEEKQGTCGFTGFVNLLSIYVMLMTLCLVSIDRFLF
IVKPHLHKRFMTPRIALVLVIIVWSFDTAFFSSGFIDGSGVEFRYINYLGVCYAFTTSPV

MAIFRFFFASLIFIIIIIITSVWTFCFTRKFINNQSMIVGEGVYASKKKRLFGIFGSMMLV
YGIFFTPSIFFSALLAIVDFPGVLMITAVIFFFLVITLSPVIQAYFRPEINSVIANIVCC
KILKNKSYPLAISSTCNSSSAVLDIRVSSLDV

>PAC:15723144 pep:novel
scaffold:Aqu1:Contig13478:344429:345397:1 gene:Aqu1.224616
transcript:PAC:15723144 description:""
MNYSSDEVNGPAVAAILIIEMALGLVANSIVLVITFTQRQSWKQPSTIFFTSLILAHLVL
LLLYLPFFIVSSIAGEWVFGKTLQEKRDSCSVAAYIIWYSALVITLTLSVISFDRFLFIV
KPNQYKAFMKQRVALCLTITIWLLAALLNSTPLYGLGEFGYRSYGTCVPLWEDNRGYVFY
MLIVFALAVSVIIITSVWTMCFTRNFLSHQSEIAGNSVYTSKKKRLFGIFGTMLIVYVIC
LSPGFIVGFLSQFVDMPEEFYLTMIFCFVAITFVNPLVQSYFRVDTVMCIKCIHKKIERN
KSSTCYINDDTVSTRFKSKSEP

>PAC:15723376 pep:novel
scaffold:Aqu1:Contig13481:356027:357182:-1 gene:Aqu1.224848
transcript:PAC:15723376 description:""
MDYNFTTTGNINGPVLAAVFAVEAVVGFIANIIVLSITLHQRKSFKQPSTIFFTSLILSN
LLNVLVYLPMTTVATGAEEWILGSTFEQRRATCVFAGIISWINVYVITGVLAAISFDKCL
FITKPYFYKRFMKPWVALAITVTLWIIVALMFTLPSLGFGEYDYLFDYGPCYIAFRRNRG
YVIFCISLLLSFIIIIIITSIWTFCFIRRFIRDHSAMEGDSVYNTQRLKLFGIFGSMLLA
YGTCYGPVIIIGFISFAIQIPEELDAATIVAFHAIPTMTALIQSYFRPDITKPLASFKNA
ICSKLNAAHSSSN

>PAC:15723377 pep:novel
scaffold:Aqu1:Contig13481:357495:358439:-1 gene:Aqu1.224849
transcript:PAC:15723377 description:""
MDYNFTATGSINGPVLAAVFAVEAVVGFIANIIVLSITLHQRKSFKQPSTIFFTSLILSN
LLDALIYLPMTAIATGAEEWILGSTFEQRRATCVFAGIISWINVYIITGVLAAISFDKCL
FITKPYFYKRFMKPWVALAIIVVLSIIVTLMFTLPSLGFGEYDYLFDYGPCYVAFRRNRG
YVIFCISLLLSFIIIIMVTSIWTFCFTHRFIRDHSEKGGDSVYNTQRLKLFGIFGSMLLA
YGTCYGSVIVTAFISYGIQIPEEVDAATIVAFHANPIVTALIQSYFRPDITKPLKSFKNA
ICSKLKKMACSFSN

>PAC:15723378 pep:novel
scaffold:Aqu1:Contig13481:358690:359643:-1 gene:Aqu1.224850
transcript:PAC:15723378 description:""
MDYNFTVTGNINGPVLAAVFAVEAVVGFIANIIVLSITLHQRKSFKQPSTIFFTSLLLSN
LLDALVYLPMTTVATGAGEWIYGSTFEVRRATCIFSGTSNWFIVFITIGVLAAISFDKCL
FVTKPHFYKRFMKPWVALTVTVALWIIIAFILALPSALGASDNDYGFRYGACYITLEDHP
AYIGFCSLIFSIFIITIIVTSIWTFCFTCKFIRDQSEIAGENVYHLKKMKLFGIFGSMLL
AYGICYGVSMTIGILSVSIAFPNEVHATSMVAFQLNIITTPLIQSYFRPDITKSMTSLKK
FVQTQIKKLRYSPSSPE

>PAC:15724271 pep:novel
scaffold:Aqu1:Contig13491:40566:41698:1 gene:Aqu1.225743
transcript:PAC:15724271 description:""
MSLNSTDFLLTGDINAPVFSAILGIEAVIGIIANIGVLLVTLYEKKSWTKSSTMFFTSHL
LANLNVSFSLLLFSIAIGAKEWIFGITPQGKNQICIIVAYTYWNSALIIAMTLSAISFDR
FLFIVKPHFHKEFMTPRVALILTITLWLLGSMINTTPFYGIGQYGYFSSYGICTNIFQNT
SMFYLTLNVSIYSAIYLTIAVMTIWTFCYTRGFIKEQATMVGSDGVYLSKEKRLFGIFGS
MLIAFIITVLPGYTIGFISAIYPLQDNVYLVNIAFNDSIIMTNPFIQLYFRPEIKSLIGS
LTKKIRNKFKMKQHSTRIRVVPINN

>PAC:15724373 pep:novel
scaffold:Aqu1:Contig13492:85968:87038:-1 gene:Aqu1.225845
transcript:PAC:15724373 description:""

MEMDEMTILEENKNFTLSEDINGPLLAAVIGLEMITGFLTNSFILVLTICYLKTWKQPSN
IFLTNMLLNNLVIVVIVTPFSIVTCASGEWIFGNTLSEKVAICEASAYFFTFSTTVAIES
LVLISFDRFFFIVMSFEYKKYMTVNRAIIIVIISWMLAAILISTPLYGFGNYRFSNSYGM
CVPNFRNLGYAAYGSTIILVLILSIIVTSTWTYCFTRRYIKRRSSASSLSYNSNEYQSQN
RKLVGLFGMLIVIHILCYTLFVAVSALRPFYTAPRQLWATTFVLVILMTSLSPLAHAYFR
YDIRSFLHRLYMKIKGTCICSCVGTSATQKEATQSFDTSIMKTNGIHEHKTQLSIS

>PAC:15724923 pep:novel
scaffold:Aqu1:Contig13499:218831:219745:1 gene:Aqu1.226395
transcript:PAC:15724923 description:""
MDGVNYTLSEDINGPLLAAVLTLQMIGALVANGIVLIATLSQYKSLKLPSTILFTSLIIM
HLVMALLYIPSWILSAASGGWIFGRTMQVKEGTCKFAGFILWYIILVIYMTLAAISVDRW
LFIVKPQLYKQFMKPKVALTIIASIWILAALINSTPFYGIGRFRYAIYGSCVPTFEEQFV
YVVLMMAIFLAISAVILVTSVWTYIFTKKFIQEHSELEESSVYVSRKRRLIGIFGVMLIA
YFVCFSPGYIFGFLSQIYDLHDLSYAIALVFFMMITIINPIIQSYFRPDVKKAFVKLKIK
CLNI

>PAC:15724924 pep:novel
scaffold:Aqu1:Contig13499:220902:221882:-1 gene:Aqu1.226396
transcript:PAC:15724924 description:""
MEKSNFTLSEDINGPLLAAALAIQMIGALVANGIVLIATLSQYKSLKLPSTMFFTSLIII
HLLMALLFIPFYMISVAAGEWIFGRTVEEKEGTCKYVAYIFWYIILVIYMTLAAISVDRW
LFIVKSQLYKQFMKPKVALTIIVSIWILAALINSTPFYGLGEFRYSAYGCCVPVWEGQFG
YLLFIASFNFIVLCVIIATSVWTFIFTRKFIQEQAALADSCVYVSRNRRLIGIFGAMLSG
YAVCFAPGFIVAVLSQIFDLELSAYATVLSLFINVIIINPMIQSYFRPEIKKVLTAFYAK
CKRFCIRSSVHNNTEAGKSFTVTSYK

>PAC:15725490 pep:novel
scaffold:Aqu1:Contig13504:239296:240444:-1 gene:Aqu1.226962
transcript:PAC:15725490 description:""
MDLDSSFLDYANCSDQNDTTPLFINLTVVSNVVIAFHATFISLVIVTGLLANSTVLVLVA
KDKRLRHRSIVVGLNIVFVDILLTIFYHGVILTNCLSKGWSYNEQPEPDLICRAYGVLTT
VLLNIRWFGIAVLTTDRFLTVKFPFRYEKYSRRFLIVASLLTWTIPPGLASLLSLSLVSY
SFRANIPTCLPSCINATYRMACALINTGIVMIMFIIGCIIPSGMYIWMYRKARKMRTKYT
LGELALNVTTSVAVKNIRKAQNTRSQYDRQAMVTVFLIFLSILFTSSPSFLFLIVRQISI
CIFFKIPVVIHFIVTDIFIVSTALDPLVLMKNHDFRTVIYELFHKKCWCRHHVTDGRTSS
TEAETQSSSANINDMHETNLKN

>PAC:15726227 pep:novel
scaffold:Aqu1:Contig13509:476758:479483:-1 gene:Aqu1.227699
transcript:PAC:15726227 description:""
MEEEYIENDNFTLSEEINGPLLAAVIAVEMVAGLIANIFVLALSCFHCEIYKKPSTVFLT
NMLVANLIMIVVVMPVPIATCVSGEWIFGSTVSSKLASCEAMGTLFAWSTLIATESLVLL
SFDRFFFIVKAGKYNEHMTVKKALIIVVASWILATILVSPPQYGFGGFNFAESYGLCGPS
FRSVGFSVLIIPLPVTFMLHLMLNLPQVL

>PAC:15726228 pep:novel
scaffold:Aqu1:Contig13509:480547:481672:-1 gene:Aqu1.227700
transcript:PAC:15726228 description:""
MEEEYIENNNFTLSEDINGPLLAAVIGVEMVAGLIANIFVLTLSCFHCETYKKPSTIFLT
NMLVANLIIIVVVMPFSIATCVSGEWIFGSTVSSKLASCEAMGTLFGWSTLIATESLVLL
SFDRFFFIVKAGKYNEHMTVKKALIIVAASWILAAILVSPPQYGFGGFVFANSYGLCGPN
FRSVGFSVYGFFIIGSLIISIVVTSLWTYCYTKKFIKEMNSRMLRESVYFSQHTKLVGIF
GTLIIVHIFCYSLFLSVSVVRPFVAIPRQLWATTLVFLLLITILSPLVQSYFRSETRNFI
QNLLLKIRLSKSHPTLPTRMTVTSLIKLKAPSQTLSE

>PAC:15727588 pep:novel

scaffold:Aqu1:Contig13518:220905:222439:1 gene:Aqu1.229060
transcript:PAC:15727588 description:""
MEDNFTFIGEVGGPAVATILIIEMILALIANGVVLSITLYQRKSWKQPSTIFFTSLILAH
FVLNLLYLPFIIVTFVAREWVVGSTDEEKKWTCYYITFIFWWMVLVIFMTLAAVSFDRFL
FIVKPHLHKRFMRPWVALTLTIAIWIFSAIYSIVQIFGFHLYIAICLTPAAGIGVFFFLL
SVTSIILVIIFVTSLWTFCFTKSFFKDQSLIAGESVYASKKKRLFGIFGSMLLIYGICFV
PVIVLPLWFDIFATICFLFITVANPVIQSYFRPEIKIS

>PAC:15727589 pep:novel
scaffold:Aqu1:Contig13518:223413:224838:1 gene:Aqu1.229061
transcript:PAC:15727589 description:""
MESNFTLTDGISGPVLATILIVQIILALIANGVVLSITFYQWKSLKLSSTIFFTSVILAH
LVLNLLYLPFRIIALAAGEWVFGSTNEEKKVTCTFAAFFFWWMVLVILMTVAAISFDRFL
FIVKPQLHKRFMRSWVALTLTIAIWILSAALSNVHIFAFHLYIAICIRPSNSIGLITILG
IIFVTSLWTFCFTRSFFKNQSVIAAGSVYASKKKRLFGVFGSMLLIYGICFVPGFLYFFL
SLFIDVPVWFETFAIICFDFITVANPIIQSYFRPEIKTLALIAYGAVVTIYPTLSKEILF
KQSLLYHYLLPLHLLE

>PAC:15728240 pep:novel scaffold:Aqu1:Contig13521:7527:8483:-1
gene:Aqu1.229712 transcript:PAC:15728240 description:""
MSFNSTDFVLTGDINSTTYAAVLGIEGVIGMIVNVAVLLMTLYQRKSWNQSSTIFFTSLL
LSNLIIALWYFMSSIAVGAEEWIFGNTFEQKNATCLFIAYLFWNCSMNSMMTLAAISFDR
FLFIVKPYLHKRFMRPRVALILIIGVWLLCSLINTTPFCGFGVYRYYSPGGICSTSYETS
GFFYLLFLVVVYSIIYFIIAFTSIWTFCFTRRFMQNQAELVDSSVYQSRRKRLFGIFGYM
LLSYIIAILISYITGIINMFYYLPSYVSLGVVVVYGSFIISNPIIQSYFRPEIVIIIKQW
WNKIKRSSRNQDRDLNYS

>PAC:15728243 pep:novel
scaffold:Aqu1:Contig13521:18775:19746:-1 gene:Aqu1.229715
transcript:PAC:15728243 description:""
MSFNSTDFVLTGDINSPTYAAVLGIEGVIGIIANVAVLLMTLYQRKSWNQPSTIFFTSLL
LPNLIIALCYLMSSIAVGAEEWIFGNTFEQKNGSCMFVGYILWNGIMIIAATLAAISFDR
FLFIVKPHLHKQFMRPRVALILIIGVWLLSSLINTIPFYSTGGYGYFPPNGICSFLNETS
PYYFVVLILVYAISYSIMAFTSIWTFFFTRSFIKRQGRSVDSGVYQSKNKKLFGIFGSML
LSYILSFFPSYIVGFFVYFGLPDPDGLYAFTFCTYGLVVIINPLIQSYFRPEIKEVLASF
ANKIGLKSNKILPVRIQVEPKPD

>PAC:15728244 pep:novel
scaffold:Aqu1:Contig13521:24043:25020:-1 gene:Aqu1.229716
transcript:PAC:15728244 description:""
MDSMSFNSTDFVLTGDINSPTYAAALGIEGVIGIIANVAVLLMTLYQRKSWNQPSTIFFT
SLLLSSLIIALVYLMSSFAVGAEEWIFGNTFEEKNGSCIFFGFFLYYGGMLNAATLAVIS
FDRFLFIVKPHLHKQFMRPRVALILIIGVWLLCSLINTIPFYGFGVFGYLYDNGVCSLIY
EHSGLYLLVLLVVYPMIYCIIASTSIWTFFFTRSFIKRQGRSVDSGVYQSKNKKLFGIFG
SMLLSYILTLLPSYFVALLVFIFGQLDSAFAFGIGTYGLISIMNPLIQSYFRPEIKETLV
LFAKKIGLKSNKIHPIEPQVAVTAD

Adhesion like GPCRs

>PAC:15701718 pep:known scaffold:Aqu1:Contig7151:312:1956:-1
gene:Aqu1.203190 transcript:PAC:15701718 description:""
NISNPRCVYWNFTTAAYGGRFYYVGGRGNWSTDGCNTTFNTTDDDLSFIACHCDHLTNFA

CLVDVSARVNEPTQPPQYVTVTLEVVSIVGVCLSIGGLVLTIITLVIFKKLRAREASKFH
IQLCLSLIFMLAVFVSGIDRVSVRAGCITVGVLIHYFALVAWMWMGAEALLMFQKLVIVF
TDITWKYLVGISLICWTLPLLPVIITLAVDVDFYIYLYEPENQSGFCFISDMIPFFAAFL
LPVFIILIFNVIIYVIIIRVLILHTIRKNKRMSKSSFKTSDAIKMILSYSGILILFGLTW
VSAVFTFISEPNTSFIVQFFFAFFNAFQGFFIFFFFIILSSDSRDAWRSLLCPGTLRDEM
KPQTSTTLKSKTLNSKTMSLQKKNVPRNAILSFGGKNMPNEKQTDFTDNIIFENEANFED
EVFSIDDEFMMSEFNNIRFDRQLSARRKHLVEKIEIDFFDVAKDD

>PAC:15705926 pep:novel scaffold:Aqu1:Contig11591:6943:9517:-1
gene:Aqu1.207398 transcript:PAC:15705926 description:""
MATRAIRQCRRNGVWDTHDISNCADPKITAEFVSIADTNVTAENVVQVAQNLSEVVMTAS
QPGDQNEYNLRNVSSLLVQTANLFSSPNVITVLSTKEVSMATKSTVEILDNVQEWPSPVV
ATQSNNIIQSFERIVEALISQENFTNLIIIETGIAFQGLRVQKTNFSSITFSGSPLIDSD
TNGTVTCSCNHLTNFACLVDISTRTGNLPPPRPVTVTLEIVSYIGVCLSLLGLVLTILTL
LIFKSLRQRDTSKFHIQLCLSLIFMLIVFVVGIDRVSVRAGCITVGVLIHYFALVSWMWM
GAEAVLMFQKLVIVFTNLTWKYFLIVSITCWGLPLLPVAIVLSVDVDHYITFYEHTEEGF
CFLGQLNMFLTAFFVPVMIILILNSIIYILVLCLLILHTLKKNKRLQKSTMKLSQAIKML
LSFTGIMLLFGLTWIFAVFTFTSEPGVSYTVQFFFAFFNAFQGFFIFVFFIVLSNDSRTA
WKSLLCRCMMNNEQQTSKYNLSSLNSKKKRFPNSNTYSSSETGNERCLPSLSKEDNVSAS
QEKKSDDLDLVQLKGTHEKCDSTSTHECSSL

>PAC:15707415 pep:novel
scaffold:Aqu1:Contig12272:16391:17429:-1 gene:Aqu1.208887
transcript:PAC:15707415 description:""
MLIVFVAGIDRVSVRAGCITVGVLIHYFALVSWMWMGAEAVLMFQKLVIVFTNLTWKYFL
IVSIICWGLPLPPVAIVLSVDVDHYITFYEHTEEGFCFLGQLNIFLTAFFVPVMIILILN
SIIYILVLCLLILHTLKKNKRLQKSTMKLSQAIKMLLSFTGIMLLFGLTWIFAVFTFISE
PGVSYTVQFFFAFFNAFQGFFIFVFFIVLSSDSRAAWKSLLYRCMMNNEQQTSKYNLSSL
NSKKKRFPNSNTHSSSETGNERFLPSLSKEDNVSASLASQEKKSDDLDLVQLKGTHEKSD
STSTHECSSL

>PAC:15711607 pep:novel
scaffold:Aqu1:Contig13059:50341:52905:1 gene:Aqu1.213079
transcript:PAC:15711607 description:""
MTVTILCPNGPNGATASRICSASSDWESPDVMMCATTDVTNGFIELSKVNITVDNFHSAA
FNMSSLVENATRTLADQNIENINVISTVLEAIISVLLKSKSAQLIIETTRYFVLMLNSLM
EWPIGVTNVLINNIIQSFEEFIQVVFEQKNFTVIKIAEENILFRAERVILPKNIKNVTNS
ENIDVVSTLYKKATFFPVHEEVNSSSSVMTVVGSAVISIIVGGIPDGTELIDPVKIILAL
NKENVTNPRCAFWNFTGADGIGNWSIDGCTTVVDPNDLSNVSCYCDHLTNFAILVDISTR
TANGTSPPFGVRVGLEVITYVGVCLSLVGLILTILTLLVFKKLRKKDSSKFHIQLCLSLC
FVLIIFVTGISRTSVRPLCISIGALIHYFTLVFWMWMGAEAVLMFQKIVIVFTNITWRYI
LIVSIVCWIMILLAVDNGYYVTSTKNSEFCFIGKMVPLLAAFLSPIFLIIIINTIVFTLV
IRTTLKNAIDRKKRMSNSSLSVSEVLKMLLSFTGIMILFGLTWISAAFTFISEPSVSYTV
QFIFAFCNAFQGFFIFIFFILLNKEYRKLWKAHFVQCFPKATIHSDYKSGHSTKSKSKNE
NNYAISSHVSLNDTNKEEMKDTSL

>PAC:15712029 pep:novel
scaffold:Aqu1:Contig13091:39856:58653:-1 gene:Aqu1.213501
transcript:PAC:15712029 description:""
MELTCPLLSALPFLFFVLFSALDLAHSQSCSNNGSVRLTTNSGYGAVELCFNGTWGSICR
DFWDNNDASVVCSQLGFSPYGAIASPVYRSSTLSSHHNFNFTCNAKNDVAESNCTDGEVR
LIGGSNDYEGRVEVCINRAWGSICGSSFGTADANVVCRRIGAIPNGRFASRVGSLGFPSG
TATYPLLGSLYCFGTEPDLTKCNQNYLHARTYSFCRSRSNDAAVRCELRGGRFVPIVGAM
KRQLWRVGNWGSLSAVPGIDYSRWPVGLTVNCTGTEDNIWDCAYSLTDEIPNSCSRFSPA
SIYCYNSTIPDNCTTGDVRLVGGNAVNQGIVQVCINHYWGTTCRQSWHNSETMVACNQLG
FQRYGGMQVDSRTFELVTSRPNLFTNMYCNGRENSLFDCSRNMFISSLVSRTCGSRNYDV
AITCEVPCTSGDIRIAGGPTSLIGRVQICSNRTWGTICSDYWDTTDARVVCKQLGYSNYG

AIAIRNAFSDSLWPTHITDLNCIGDEDSIWNCSFNRQPPRNCFWDASVSCQCKRSNNTNV
TSCKTGDIRLVGGQTIYEGRVEICIDRVWGTICGSNWGIPDSNIVCKQLGHIGLGSIAYT
TSNKFGRGIGPIHLTNVRCSGRESNLLSCSHTPYISPSSCTHFNDVGVKCEASCENGTIR
LYSNSDSYFRRYGRVQICINNVWGTICDQSWDDNDASVVCRMLGYSPYGATALNRTFTEG
VWPFHIRELNCTGSEDSIWDCPHDNRPRSNCGTTNDAAIPSDCSTGDLRLRGGSTQYEGR
IEMCYNGLWGSVCYSGFGYNELLVVCNQLGYQRFAVRASSAQFGSSRLPVFTLSGRCTRS
HTGNLTSCAQKTLPGPFCYRSNEARVICKARCTDGDMEYDTSSSYGHVRVCINGTWSLIC
ASGLTRRDANLSSVLCSSLGFSQYGATYSTSYINNGFYFYFVYDVICNGSEKNVLDCQRV
LSNVRSCFQPVTVYCQKEGIEAPVNCSDGDIRLYGGAAPNEGTLHVCSSNSWNNNTRTIH
RLTNFQFPITVQHRECRGSEERFSDCPVRSLISHSTPCVVDAVNITCSAPCVNGSVRLAY
PDNYYYGQVLVCIGGVWGSICRNEYWNHADASAIALPSSQNGDANYISILSGLNCTGDEA
SFTDCIVDSNAPVCSRFNYATVICPVPAGEYSNCTDGEVMLVGGDTEYEGTVQICLNRAW
GTVYPNSWNINSAQTVCNVLGYTAIGALPTRNAFHGRGTGPVLFSSIACSGPQQSVFDCS
VSLNTIGREHYNDAGIQCQPFCNSGDVRIIPDNSRMPNEGVVEVCVNRTWGTICDRSWNT
TAANVICRQLGYSPTGAIIISSRYYNDYKPVHIGDINCTGSEGSFTNCLLNYTTQAGCTG
FFNDARIRCAVLPETNCTAGSVRLVGGNTPEEGRVQICFNNHWGEICPLSWTTPDALVVC
RQLGLPTVGAYFTTEFGKGDILTVNFTTAYGCRGNEPSLSNCSFSVSPACSLFTYHAGVH
CEAHCSNGDIRLRADHRYDNFGRVEVCLNGTWGTVCTNGFDSNSASVVCNQLGYSPYGAI
AMTRSESILPHNIYNVSCTGDEESLLNCTYSTKPVAGLTCGTSDDAAVICQDLSVEYSNC
TDNEIQLVNGPSPNEGTVQICHNGAWGVLCYYRYSNAINQAVCNQLGFQTEGAVATFGRY
SNVPILYSSVACQSTDKYLSDCTFTGQIGSSRCGSKSIIGVRCHHCTNGEMRIVQRSNQL
FPQYTVGRIELCVNGSWGTICSDFFDNSDASVFCRQMGYSPLGAVPLVGYFPEGIFPFHI
VDLNCTGDEESIGDCPRNGLLSEYTCNGNNDAAVSCIKNEEAQYANCTDGQIRLLGGSNP
LEGRAEICHNSMWFGLCPTFQSTYSYNAICKQLGHLGQNARSSLDPFFKLPVVPYFHKQF
SCSQTEDNLNNCRIVSTLCPPQNNVYLHLKCEERCKNEDIRLQGVTGQDNAGRVEVCVDG
EWRSICQDDFEYTDASVVCKQLGFSPYDNNATIDSSCGNGDVRLMGGANELEGRVEVCYN
KMWGSICGTFWRSNSANVVCNQLGYQGSSTSAFRGYFGEGLGPSITSQVSCGGDESSLLF
CTNDTFSALRCGHLSIANVRCQESCADGEVRLNGSSASSIGRVELCIAQVWTTVCHMNWD
LMDARVVCRQLGYSPYGAVPTYNCFTEGQLTFGITDIHCNGTEDNLLNCSHSQEPLLHSC
RSHEDAGVNCQEMSSVVRANCTNGEVRLVNGSGPHEGRVEICINEAWGSICSNEWDDKEA
DIVCRQLGYLPLGARVKSFGPGSSPIFLSNLYCIGTEDNLLECHQQPCRTHNCSNANDAG
VVCERPCTNGNIRLGDDISGLRGRVEVCVNMSWYTVCHHGWTSKEASVVCHQLGHSKHGV
LAATTVFDYEWPVGLFNLHCNGSETSIWDCNFTTTNDVGQTCGRFDDASVFCIPDSAYDN
CTYGDVRLVGGDTDYEGNVQICSNNTWTSVCSGRSWTSFYASIVCRQLNYDIGDCYDDYD
YYDDYEDDRNKREIDDYDNEESSSCRSKALFNNYFNTNYTPAFIYSDFRCRGREDSLLDC
FKFTDFERLLECSATTAIAGVRCSGIRVCPQLPRPGNGSVSIRGSIAYYSCFRGFRLNAT
NNTRYCQDNATWNVVDCGLLPNPEGGKVGIERSTLGGIAFYACQANFSLGGIPVRRCLEN
STWSGIAPACFNQTVPNDIPGRSVIFEYIYQINIEIPSLEPPPSRPPPGQFAGRKKRATS
FRLSTHERFILSLLRETNGTHIVNCTVMQSDDGSDNGPLRPTREESSSSNQPVMCNITSY
STTDLSRSITSTVFSGLLSDAEQLNIVQSSIVYGIGGFCEPNITNSSLYGSNGWPKTGGG
SSTTTSCQYGHYLYWDRDDIDAPVHRYCQPGGEWSSPNYTLCRDTRPYVSILGQRHSVDY
NVTFQEGESVDIICQTQNALRIPGDIITWYDPKGDVVPHESSLINGSSFSEGVTISDFKQ
LRVDIFSPFSTLTSYYNRALGHSISPSRSIEGTYVCEGSNSHGSRSISVTININAPNGPY
SSVFIRGNLTNITNFGSITEEQYKLAVIEDIILDNITTSNKLHRVNCKFVALRPEDQNLA
NYSRIDCSIREYPPGNASLELGQSVTKNFDIENSTLVSVTGFCNYEESVHSKYGNYMWSE
SYGGTSHTKPCPNNESKSIIRHCITNGDGWGDIDFTPCRELYIRFSSNFTSVIPSGNSSA
VLDNIRIIIELAVPAKASCGQVEDESDSSNSRIECVITGTMESPPTAATLQMLTNNITSA
LVSRQYTVLRSTATSEDGFCDAQTEITSSPDRGRYEWPEAQSNTRKSLPCVYGPGRARAT
RYCLSRGNWSVSDTSNCATALTLKFRNLENVITEFNVTVENLEAIVTNLSVILNNTEDTA
DRNRDNLQVVLKILRETGDIFTESLEEFDIETIEMIVNNTVNILDDVEEWPLPVIQPSVN
IIIQSFEAIAEALVTKANFSNITIASEDILLRGERIMRDFFNNFGRKFTVSTENNGLNVE
SEKANENGLLPEESASISVPASIISLIPNKENVNVIFVAYNNTFLFPIRTGRRDSNQTST
RDLAIGSQVIGFSIPGIPEGTELPEPVNISLRLANPKTGTLENITSPTCVFWDFKAAGGR
GNWSTSGCRTIVTDSSFSTVRCECNHLTNFACLVDISARTEEPTQPPKAYRTTLESLTII
GVIFSLVGLTLTAITYLIFKKLRVRDASKFHVQLCISLFLMLLIFVSGIDKISNRAGCIT
VGIFIHYFALVSWMWMGAEALLMFQKLVIVFSNITWRFHLVVSLICWGVPLLPVIITLSI
DPDNYITYYQDTSQVEGFCFISNTGAFFGAFFVPILLLIIFNVVVYILIIRVLVLHYLRK

KKRQSKDTMSAKEILKLMLSFIGIMSLFGISWIFAVLTFTSKSPRAAFGAQIMFTLFNVF
QGFFIFIFFVVLNNDTREAWKNLLTPWKNNRDYSKKGSSSKATLSTTSRGHDKSSKSSHF
GKKGALDHNLKQRNQSLPSIHSLDCNVAVPNIYSMIDDEETTINEPPSVLYEEVKKEKGE
QEEEEEGDKAYLNGKAIKDVRIERKFTVRNTHQIEKADLDFKSKDDDDDSADDDADAF

>PAC:15712840 pep:novel
scaffold:Aqu1:Contig13153:44375:50033:1 gene:Aqu1.214312
transcript:PAC:15712840 description:""
MICESLTLESPRIIVPPSNTTGDLYGTVVLNCTVSGTPQPRVTWFKDGREISDQNLDEYS
FVIRELSLPDRGFYQCQAVSEMPNGTIQTTNRTGPVVVNINGIVQYTASLYVPESKRDSL
ATATPDESRRILQEFVDEVNEIVAGKILAGGNELFFIELLASSQYNSSDHTVPVLLTIIT
KERGSLELLIKVRDQLKKLNIGFKVDGIFDRFDGCSSETILIPSVTDPSLAISLTWPETN
IGEEAVVWCPCGGVSLGNGTLYARRYCGGNFTDGREWGMGHFSPCNFTDRARRICKLAEL
PPKDLVKRLTSENIDDFDPREISVLSSVLLSAQDGVIGQTNASKDMFSVTSSIAMLDSDT
LRMAQIQSSAPSRLLQNLEIVTNNLPLVDTDSVLTKLPAFTIEVRKVEKGDFQGETFEIG
NKEGTQRQRQATGGVNAASISVPPSLLEEAGELEDGGVAVSSIYHRSTSLFVEEAGKSNV
GTSVISATLVHNGTGVSVRNLSSPVVIRFEKPPKQSGLINHFTCSYWSESTLDWSNHGCH
LVNDSNEFIVCLCDHLTNFAALLDVSPEPSSEPVDTDIGKALNALSYIGNIVSLFCLGIT
IITYLKSRKLRRSDHGQLLLNFCLALVGLYLSFILSLHSTSIDPLCAIASILLQYFLLVS
FCAMATEALNLYVKLVIVLGKGISRYVLKAALFTWVTPVFVVLFCFAPGYKYYMNDNFCR
AEYIPFYIGTVAPFILIYLFNWAMFTMIMAQLLRKKCRKKFAESYSDETGMSFKQQLLIA
ITLSILFGLGWGFGLFVSATIFTSATIRNIFSVIFILLTSFQGLFIFIMHCLRSAEVRKE
WQRWIFRITGVKRFEPPSSVTNSSVIRRRSTKSSSFSKSTLRREGTVESDESGPITERTI
ELSLGKALDDIVFPVPPIELKKVSELFPSTSGPHFGHDTDSVNCVSLDNPFQFSTCDAPD
SIRSPLSPLAPHQISHLDNPLTTSDSDKNGVINDSISSHYVF

>PAC:15712844 pep:novel
scaffold:Aqu1:Contig13153:60195:69264:-1 gene:Aqu1.214316
transcript:PAC:15712844 description:""
MLCLVTGMLFLLIAFCPIGAQGACNSLPEPNVKTLTVAVGATFTFRISYNRCLQNGVWSS
YTNPNLANDPSFTVTVEQDSSLFWSSTLSSVANVSKIGQYYFTIYNSNQESFSQTFTLII
ETPPQVSIMPMMKLNAVQGDNASLVCTSFSSPVTIEVICNSHEFTNSSDVFVSVYFAAYY
LTKPPTMIQELAGGNISLFCSVTANPLPPVVYWLTSNGLNLTNISTSVVASPKVIEKCKA
DGLPPPSITWLANRRQIGGLSSTFTITTVATDSVRQDVPTFTSRLNATDLLPTHNGSYEC
VVENQEGTARIQYNISVIPTATPPAEPYFFASYNRTFYHEFGSTVLLSCPVTGYPQPNII
WFKDDVLLVGAITEDYEIKKLDLNTRGIYRCEATNELGSIKSDNMTVKIKGLVQYLITVK
FSKEFIDNFFGHETRRRRQDDDDTYKATEERLNALIGRVESDLMVLLKENNSLVFEFVSM
VHHGIATSLLQPIQLLLTILSDTGGSDLLKSIVEDSFNKLKQKLAMAFETDGNPFSSTVA
RFDGCTPATTVIPSPNGDPNLDFAFPWPETDLSQLITLRCPCGPEGFDIGVIRNATRKCG
GNFENGAVWESPIDEACNFSTSARELCALASLEEPVEVLTGLDNITASTEDIGSLELSIS
TVFVENLTTIVRGDRTFTETFLTVTDNLLSINENAIIESNEEARTSGRLLQSFEGVVDDI
PVKSTDKNTIILRTKFAVTVQELNTNSFDDDDSLIFSVTNNTKNFSSANSVSFSDDLNDP
SGQIVIPGSILMNVTLNSTVRLTQTAFDTDALFLRRNSLLMKYSYLQVGSILVSASFVDY
NISNQEDLINITMKKDKIHHINAICSYWNYTADDYYGNWSNYGCSVLYEDSDIVICGCNH
LTTFALLLDVSPEDDPNRPPPFTEFATALTFIGCAVSILCLVLIILTYSIEKRLRNTNNG
KLLINMSCALVGLYVSFIPASYAAPIEELCVVVGAFLHYFSLATFLSMASEATILYVELV
KVFASGKGNMIMKAILVTWVTPFFLVVLTLAPDYKNYVYDRLKQQCKMKSQTGNANLGST
TAKQLMLMITLSVLFGLGWGLGLASTSKFPIQWLRYSFEVAFIIAVTFQGLFIFILYGIK
VVKVRKTWLKWFYLVTVQREKAQKVEYSLKSTSYSMRQSSKKLKAMTYSLGTYDSGNVEL
KKVNHQSQFNSMSPSPEPADKPAITDSTVKKLLILEEGKLKTEKLANGCKSEDLDNKSGQ
A

>PAC:15712845 pep:novel
scaffold:Aqu1:Contig13153:69456:78702:-1 gene:Aqu1.214317
transcript:PAC:15712845 description:""
MPHLITRIGLLVLLAFIYTAGASCTVSAPSGPRTIRFGSQHTITCQFSANCFSSSWTKDG
LSLSNNPNYTVTSGSTRSSISFNASLSDTGDFVCNVIKFHFSDSSIKRYFRIFVEKAVEV

HIYPNAINSVEGEMVSINCTAIGAPTPSSISWYRNGLPLNPAPESNKSTDQQTVTSRLVL
QPLERNDEGVYSCSAFNAPSSIGFKTAHSSDVLVTVYYLPLFILQPPFIVEGIAGGNTSL
FCSVTANPLPPVVYWLTSNGLNLTNISTSVVVSPKVIESTLTLENVQLSDELNYSCVVEQ
VIPSNNNRTLIKAQTQLLIAYPPQVSSPIHNVTVNASDEVTLLCEGFGNPRPVIEWYFIG
KDGNEVQIHNASRSLPLSDRTIVSNLTLTNVTIDQEGSYRCKGTNNVTNLIGSKEDTLIH
LTVHSPPQLIRVNSSSNYSLLVNESITLSFSINFASPPVKSEQVAWTLTTVNREIKELNS
SDQFQISADGLSLTIFSVSLEENGFIQSTVYNEVGTDSKGYYLLIGTSPSVSILPRHVGV
AIGTRNISFTCSSSGHPIPLLTWYANDQELSLSQDKYSSLYTNTNGTSVLTLFDLGLSDA
ARVTSIPNPPVNSSSIAFKRIDNESLSQVDIIYDESMRLVNISFTNISVSDNGTYQLTMK
NLAGEDTIDNISLNIKALPQIYSTVYSILLQEGDSSFIECKADGLPLPSITWLANRRQIG
AFSNTFTITTVATDSVRQGVPAVTSRLNATDLLPTHNGSYECVVENQEGTARIQYNISVI
PISLSFCDHHPCSNGGTCVNGPSGYLCICTEGWTERNCTKFATEKSSPQFLSYNRTLTFY
REFGSTVLLSCPVTGYPQPSIIWFKDDVLLVGAITEDYEIKRLDLNTRGIYRCNATNELG
SIKSDDMTVKIKGLVQYQANITFSQKYIRSFIDQEFRKKRQVEETFKATEERLNKLIGTM
NSEMRKILSTNKSIDFNYISLELLSSSNINSLLDSITVVLSLESKSRGTAELTFAVKNAF
SLLNGRLEALSHISDSVHVDDVRRFDGCPTEQTTNPSPNDDPSLAFILNWQETDLTQVAT
LRCPCGPEGFDIGVVRNATRRCGGNFKTGAVWEPPIDEACDFSIAARKLCSLASLQNPEK
VLAGLENITAISSDMGPLELSISISLIDNLKSEITGNTELTDSYFTIADNIISIDSDVFI
ESNEESSTSKRLLQSFETITENYPIPTDETTIVLRRRFIVVTQGLNVSRDRNNNFSLSIA
TNGNGFSNTNHPVSLNNNVTDPLASIFIPGSILSNVISEATSTIRLIQTAFDNDLLFLRR
SSLLNKWSHLRVGSILISATFAQHDISNQEDLITITLKKDKDLKANATGVYCSFWDHSSD
DYYGNWSNYGCSVLHEDSDIVICGCNHLTTFALLLDMLPNEGTNTPPLPYQKFVSSVTFL
GCAVSILCLVVIILTYSIEKHLRNTNNGKLLINMSCALVGLYVSFIPASYAAPIEELCVV
VGAFLHYFFLATFLSMASEATILYVELVKVFAKSKHGMIIKAVIVTWVTPFFIVGLTIAP
NYKNYVYEESKFCRPTQWQFWIGFISPFVMIYLYNWIMFLIIIVSLFKQQCRTRGGGNSN
LQTSFKKQVLLVITLSILFGLAWGIGLVATSSISIQWLRYTLELSFVIAAGLQGILIFIF
YGLRISKVRKTWLKWIYIVTSQHVKAVEIDQSLRTTYMIRRSTRLKQSTYKLGTYADTNV
ELKKYKLPLSQSTTANSYSSSTNSSATTADTPLADSVKEREKVVHFIDNNDKLKEDKLTA
TDEHRQVVDDEIDSRQITDVVEENDASQENDASQENLEKISISDLVPEPV

>PAC:15714390 pep:novel
scaffold:Aqu1:Contig13237:64596:65582:-1 gene:Aqu1.215862
transcript:PAC:15714390 description:""
MTTTIHSSSQVHCTSNHLTSFAVLVDHSGVIETVSDGESLALSIIGYAGPVISIIALIIT
LILLILLRKKLQKGPLLYVHVNLSLSLLLALLVFVLGIELPKSIPWLCSVVAGLLHYLFL
CVFCWSLAEGIMLYILLIKVYGSLADKWYLLLPLGWGVPIIIVGISAGINYDLYGTENYC
WLSFNKGMLWSFVGPILLIILVGDLLYFITIIINYVD

>PAC:15714837 pep:novel scaffold:Aqu1:Contig13258:6528:11841:-
1 gene:Aqu1.216309 transcript:PAC:15714837 description:""
MSPLLSCAITVALLLSVIRTGESQLSIFGSNGRIRNNSYAINDRYFFVLSCHSNQQNSVT
WLDSNGNPVLEESQFTNSSFTKASDGSRNLVNGVSAQDFRQLFLVPNLGLGVSILRFYLN
TEGDVILPNSSIAGTYTCRSGQESQAVDVSVRSPSGSFAQLFVRLTALLSNITNVNNEDY
RLAVLEGKMFFLVSLGLNLTTDPIRTISCSSVPLTPPHIGNNSLGRFECNVREHPPINSS
FVQSVSQAFVNGARDNPNFQDLATNVNGFCNYNQTVSLQYGIHTWSESVGTVVLALPCSN
RPGHNVTRLCQINGTGWENPDYTQCETSNNCPSQTIVNDHGTFEWPVTSTGVLASLPCPC
SPIGAIATRECRSNGVWGSPNISSCVSSRITDEFTILAEADVNVNNVVEVTQNLSDLVDM
TTERVDQNDINLLSVASVLNESANVFSSFRFIILLSIEDVSMTTESTVEILDSVQEWPPQ
VVANQSNNIVQSFERIVDALISQENFTNLTIIETGIAFQGLRIQRINFNGITFIGSSSNE
SLLVDTVELSKFSSLDELDLATISLPTSISNETTADDINLAFTLYTQSTLFPIRDSVPDT
VVGSSVISASVGGIPDGTVLSDNVTVNLRIVVEMVEDTSSRIGGTMVPPHTVTVALEIVS
SRIGGTMVPPHTVTVALEIVSYVGVSLSLLGLVLTILTLLIFKSLRQRDASKFHIQLCLS
LIFMLIVFVVGIDRVSVRAGCITVGVLIHYFALVSWMWMGAEAVLMFKKIVVVFSNITIK
YLLVLSVICWTLPLLPIIIVLSIKEDYYITFYSNTNDQVGFCFLGHLTVFLTTFLVPVFL
ILIFNLVMYILILRVLILHTVRKKQRQGKSTMTPSEAVKMILSFTGIMFLLGLTWIFGIF
TFISEPGVSYTLQFLFAFFNAFQGFFIFIFFVVLSSDARAAWKSLLCPWSIENKQKTSKC
HLSSDKKTMIDKKSDFSDFSKNSSDYSSLPPSLPTPFLPSLSENENRVDATPKSIGDIEF

KGARITRRSTRKHTHHIEEVKLDFFDSDTDDYFENDF

>PAC:15715470 pep:known
scaffold:Aqu1:Contig13286:116289:118263:-1 gene:Aqu1.216942
transcript:PAC:15715470 description:""
MSVDVYNNGGSRVPVAGLSDPVMVSFTVTSSNSMSECAFWDSTSFTWSNDGVTTVRSTTG
ITCMATHLSSFAVVQRGPVSSSTTPTPTPTGVTPPTEPVTEEYSPEPILFVGLIISILLI
LVAVIGHLASDAQRHCPEGRLLATSHITLMILYFVFVLATAATYTDSTGFCGTFAGLFHY
FSLVYFVWLMFEGLFYAVKLRMGIGGNVFTRHFFIVSLIVALVLPAVIVIISAAAGYDYY
TVENAFCRPQMHLFYYGYILPMIVIYLLALLNYIISFFLIYCSGMTSAEKQFRKDSVISH
YYVGLILFIIFGVVWVFAITGTQYNITGPGSTALQYVFGFFSMIHALLLVILTFARTSDT
RLAFRPVTRMIPGQKGQYDFAHDPSVGKATRRETYGLDSSGKYLTNETEAATTAEKEPLA
SGDVDDEPRYVTTTMTEKEPEASTVVANEEALATEEEDKKTEL

>PAC:15715471 pep:novel
scaffold:Aqu1:Contig13286:137526:150978:-1 gene:Aqu1.216943
transcript:PAC:15715471 description:""
STNIDLVCSTNYQSKTGLPYPLVGSAKDYGVNFIPNYITDFSCPADSSYADSSCNYTTNT
QSCAGYSGPSPDDCGSGNIQIQQGSYGYGHLNNNQYLVTGYPQICVTYDYIPICRDGVSE
EELRYICYRTGYYGYTLGPVIGSESNFTAPNSTYSATNLSCPVFSEYDNCNYTTFNGNCD
TRGGPILLTCLQVPSVCRHGDVRIVDGPNNYTGRPEFCYFGTWRSICGIGFDLYDAAVTC
SLAGFNHDLARNLSAGSYGYNNLPPVLAGLQCTGKEGSFSNCTPINGDLLSSCNYTQAAA
VACEPVILAINTTINGTATLGPNTNLTLTCTLDYQFLSGDFQLNSIGWYRNGARLSSVSG
RYNLTESEVEQYRCRYYGYYGCDNETVAVRLVLAFENLMLSDAGEYDCRVNLTDFNVSRT
VVNSTTETLNIIRDGTSSTSSVIVSTSVSTEVFSSTIEPSTTSVSTGVFTSTIELSTTSV
STEVFSSTIEPSTTSVSTEVFSSTIEPSTTSVSTGVFTSTIGPSTTSVSTEVFSSTIEPS
TTSVSTEVFSSTIGPSTTSVSTGVFTSTIEPSTTSVSTEVFSSTIELSTTSVSTEVFTST
TSFSFSSSVSISASSFSVTPTPSSTPAVGEPCYSTNNARFGNASRFTFYPGSQSVYTGRV
EVCPRSRVYTPVCNDAINDQFAYAFCRRINYNSYPVYELLIGTESDYSVYNTTSLMILPV
DVTTSTSECSDGNSVRLLNYTRVYNRYADFETVSSGRVSGCVNSQYVDICADGNSDLEAI
AQRACNDRDYNSRATIIPWTSDYSMSSSGIVLRNVSCGSDVYSLQGCTYEISNITECPSG
ILAVTCETTCSNGRTRMVNERVEYTSNGSARVSGRLEICINRDWVSVCDDDTLSEPLADI
ACNSIGNGFNSGVFLPSNTSLYGSNDRVYTNFTCEFARSCYSSYQSLYYSSFITTADGRY
GFIGRISTCINMQEAVYCNNTITSSNIDLVCSGNYQSRTGLPYPLVGSAKDYGVNFIPNY
ITGFSCPADSPYADSSCNYTTNTQSCTGYGGPSIVTCINGPYSCGSGNIQIRQGSYGSGR
LNNNQYLVTGYPQICVTYDYIPICRDGVSEEELRYICYRTGYYGYTLGPVIGSESNFTAP
NSTYSATNLSCPIFSEYDNCNYTTFNGNCDTRGGPILLTCLQVPSVCRHGDVRIVDGPNN
YTGRPEFCYFGTWRSICGIGFDLYDASVTCSLAGFNHDSDSNFQFSMARNLSAGSYGYNN
LPPVLAGLQCTGREGSFSNCTPINGDLLSSCNYTQAAAVACEPVIPRSCYSSYQSVYYSS
FITTTDSRYGFIGRLSTCINMQEAVYCNNTLTSTNIDLVCSTNYQSKTGLPYPLVGSAKD
YGVNFIPNYITDFSCPADSPYADSSCNYTTNTQSCTGYGGPSIVTCINGPYSCGSGNIQI
RQGSYGSGRLNNNQYLVTGYPQICVTYDYIPICRDGVSEEELRYICYRTGYYGYTLGPVI
GSESNFTAPNSTYSATNLSCPIFSEYDNCNYTTFNGNCDTRGGPILLTCLQVPRMCYHGD
VRIVGGPNNYTGRPEFCYFGTWRSICGIGFDLYDASVTCSLAGFNHDSDSDFDFSMARNL
SAGSYGYNNLPPVLAGLQCTGREGSFSNCTPINGDLLSSCNYTQAAAVACEPVILAINTT
INGTATLGPNTNLTLTCTLDYQFLSGDFQLNSIGWYRNGARLSSVSGRYNLTESEVEQYR
CGYYGYYRCDNETVAVRLVLAFENLMLSDAGEYDCRVNLTDFNVSRTVVNSTTETLNIIA
GPQLISSPQNYTVINGSNVMFNCSFTSELMMTAQWYYASLTGTTNGSPLTSGVRLVVSPT
VYNGVNQYSTSLSINTAMVPDIGVYQCVGSSTNGIATGYGSLIVLVAGTIDVTAVAQSIE
EGNTLTLTCTVSSSYPEVSTFSWFRGSSLITLTNRVTISTGSTIDTSTGLYTSTSQLMIQ
NTLTGDSGQYLCRVPPVFSLPSLLDRIAVSITPRNECIIDGGSTPCVNGYCVDEVNDYYC
VCPDNFFGRNCETPVVINSAPVIVTPPMDQDVPLGSLVNLTCVATGAPQPVITWYLEGTL
IPGANSPIYIIDSIQPAQRGTYSCRAMNSEGMVQATATVTINDIRQYVTTLTASSSRKRQ
VQLPPAIEAVYNQTRDTLVGNELSPGSGIIVADITYALASDGAGVLFIFTLRVPPMANTD
GITNSLNALTADIAANYPGISFSPVMRFDGCSPETLPVTAGSSLNIMWPEVDVRVVARPI
CPCGDITTNFGAVGIRRCNGTFSAPAVWLTPNTTQCIFDQTTTRLCQLSMNDNAEEVATQ
LTNITTNTQEIDPVDIGLIGTLAEEVAEDASDPEVLDSLMSTMNNILDAPDSVLEESQQS

SNGSQRIVNVIEAMARTANITRGGSVTVVRPNVAIEAQAVEASTFNGAEFSIGTTSTGTL
DENTTSSSTNVNSVSIPPTLLTNVGVNGSARIGFSAITNSKLFQPRDTSSNTPPVRVESI
VLSIDIYIDGGSRVTVQELSDPITLSFTITLNTGSSRRRRQATPSNTISECAFWDTTSFT
WSTTGITTVPTATGVNCIVTHLSSFAVVQRDAPVSSSTTPTPTPTGVTPPTEPVTEEYSP
EPILFIGLIISIVLILVAVIGHLASDAQRHCPEGRLLATSHITLIILYFVFVLATAATYT
DSTGFCGTFAGLFHYFSLVYFVWLMFEGLFYAVKLRMGIGGNAFTRHYFIVSLVFALILP
AVIVIISAALGYNYYAMENVFCRPQKFLFYSGYVMPVAVLYLTTLISYCVSFFLIYCFEM
TSAEKQFRKDSVISHYYAGLILFIIFGVVWVFAITGTQYNITGPGSTALQYVFGSFSMIH
ALLLVILTFARTSDTRLAFRPIARMIPGRTGKYEFAADTRGAQETYGLDISGKYLTDETK
MARADEKEPLAVSNIDEDDEPRYVTSTSVTVVVNEDALEGDEKTEL

>PAC:15715659 pep:novel
scaffold:Aqu1:Contig13294:88816:94720:-1 gene:Aqu1.217131
transcript:PAC:15715659 description:""
MNTLTSLHFYTDSCNGMSSSHLKISKCIFPDACMLEYDGQNISCTVSKKEGPHFYSLTLN
VTKGESISFIWKQYFTAQSLYCDIWALDDVAVSIEYLGQSRVIYNETFNNPSGWIFYNGR
ESDDSIECDNRNGKCLFFSDGQGEANRTATSPLFDVNLVEIVTLPSVFSSNVLCDANDET
LIHISFLFQLHNFTQPLPITDSFPNTILLSLSTDQYEAPLAYYAPVVNAGHFGIMPMPTS
SLSGILPSSTPLPLFCESNGTWPKTSANQNVSGFCHNGIINANRFCNRDGVWEEIICHTS
EDFNRISQEIKQDPNLALSILNETREAFSSREAVVLLNMIVTSVENVTEEHTEQVIGISE
SIIRMKNISNNKEQRRIAGTRLLLILDELALKLDSNNSVILSETITQLTFLFGVQRIENK
VACSIIATVMQYMFLVTFMWMLMEGAILYIALVKVFTTRTRHYAVAFTLVCYSVPAIYMM
LVVPIGYLVDTGNNQSNHYLLYNNDELVACWLSYETGFIWSFIAPVILIIILINIGFFIMA
IVIMKRHKKAQPNQSFYNDIKYWIKCSMSLTCVMGIAWLGSVLFFREELLFIAYIMTVLI
AGQGIVIFVLYVPLSKHVRAAYSKWWNDKKANSEIIGSLSLRASNLLRPFSFQQTSVSVA
IKADQYLPKHCENGTSKDSSDTLNKARLDQILNESGMKDESFIIANEIDDDYLTVTDS

>PAC:15716310 pep:novel
scaffold:Aqu1:Contig13316:88085:89670:-1 gene:Aqu1.217782
transcript:PAC:15716310 description:""
MVNGSDVGPASFPSEKHLLVRTTVMIVCSLSMIGAIFVILSYVCFKSLRSQARQILVNLS
LMDFGIGAANFFGAIINFDQYYKDMNNGTVLTGTHAMDSLCKTQAFIALLTTYCSVFWTT
SLAVYMYSLVFLNLKSTKKMRLCLPACYVLCYGLGIGLSIWLILTNRLGHSPYNSSGWCS
IIIISPGNKNVDFMAAVFGYDLWIYLTFFICAVVYPILILQMNIEFKGAIQLIGSKAFWK
KALEFLNFKLFLIPIAFLLLRIWSCIVNIVFVYCGVDPDSLPHWVSMFLIIMSGIGDSGQ
GLANGLIFVFLTSSVRDKFLPRCLKKCKHTIHINAEERSLVLSSDKLDEEARRVSPNSIV
TPTSSLY

>PAC:15716311 pep:novel
scaffold:Aqu1:Contig13316:89980:91114:1 gene:Aqu1.217783
transcript:PAC:15716311 description:""
MDFGVGLFNFLGAVINFNQYYTYYDPKTGLVTADSHPTVEKICISQAFLAHFCTGASVLW
TAWLAVYMYMLVFQVYFGSKKFLSVGYAICYGLPLGMSVWLLMTDRLGYSIYNEAAWCGL
IVSNSTALTGLPDYFAITIGYDIWIYLTIILTATIYPVLFTYLHIEYGQARKLVGGIQYW
REALVFLDVKLIAIPIAFVILRMWTAILNILIIYAMIPPDNIPTSISTALIYLSGIGDSG
QGFVNGLLFVFLTREVRKRLFCKRRHKQEDKRQLLTSENQRIHGNVRCNERQRTYSPSES
LRVESDVDG

>PAC:15716762 pep:known
scaffold:Aqu1:Contig13334:88161:92636:1 gene:Aqu1.218234
transcript:PAC:15716762 description:""
MNTNNDTYSLLVALTLITQKVQGDKMLLSSVRHVLDSLGQKLSIQISVTAVERYDGCPSS
FTIIPSHSGNPNLTITIVWPETNIGVLAVVDCPCGTNGTSGGGKLQATRYCGGDFTNGAV
WDAPDVMRCNFSDLARAICRLKDLPVGERVRELETLTSDSSALGPTEVAASVSVLVSATG
ELEGDITLTTTFLDTVDNILVVNQKVLQESQESSNTSSRILDAVENVVGDITITNSSEPV
VITQNSFAVLVQQIDLEELDESSQAFSDVSPKNKPTERSELTLFLDSISYVGIMVSIVCL
IITIASYLLSKKLRSSDHGQLLLNLCFALLGLYLSFIVALHSKDTNIFCAFSGAVLQYFF

LVTFIVTAAEAIDLYINLVIVLGHKIDHFVLKATLVSWIAPVFVVLFCFSPDYKSYISDP
PNFCRAFKAPFYIGVVVPFVLIYLFNWIIFVMIIVSLSRKTLSLKLNNVKTKKESKILKQ
QLMIAITLSILFGFGWGLGLLVTEDIYTSKTVRDLIASVFVFLTGFHGLFIFVIYCLRSK
EVRFVWKNVFLCKKGKEFSTTNFNRIQKTSTGTTTMMSHNLAVSLKKDQTCKFEEGSQMR
VHDYTKKNEPREKELNKSEEHINLNCIPNDDAQATLRFYTKKYQDQFNIGETEAISKVDS
KSQEPQYSVGNFGNDNKRNDSHEVDEEHF

>PAC:15716767 pep:novel
scaffold:Aqu1:Contig13334:124318:150547:-1 gene:Aqu1.218239
transcript:PAC:15716767 description:""
NMYRANLQFFTACTTGSIQLVGGSHDWEGRVKVCNNGLWATVCDNNWDSTDAAVVCRQLG
WGTRTEQLLTNCTHSTAHNCGHSKDAGIACHVCTPGTVRLVNGSNSNEGRVELCQNGRWG
TVCDDSWDNTDAIVGNESVFLANVACNGAEMFLANCTHDFDVLSYNCSHSKDAGVMCLSQ
CVMYIMRLLICGIGCNDGSIRLVGGTNSKEGRVEVCSGGVWGTVCDDFWDNTDASVVCKQ
LGYHSGTAFGSAYFGQGTGSIVMDNTHCNGTESLLKNCTHITEHNCNHNEDAGVKCVFCT
PGTVRLVNGSNSIEGRVELCHNGRWGTVCDDSWDNTDASVVCRQLGYGPGTAFYDAYFGQ
GTGSVVIDDVDCNGSESYLTNCTHTTEHNCNDKEVAGIRCSCEFGIVYRGVMTKKDQMSQ
AVAVKTLKEFLIHNNGSVEYSPTIIGSVHYVCQAQGSSRGTYRSLSIIATYTPNPGQSQT
RGIFQLMCSSESWEPDLDGGIPVPVSGLIDIPTRTDCLLCNHSFGSDRCHACITGSIRLT
GGSHDWEGRVEVCNNGSWGTVCDNNWDSTDAAVVCRQLGWGTSGTARTNAYFGQGTGSIL
LDNVQCTGTELFLANCAFTSNHNCRHSEDAGVSCSIPLPIMHSPGCIDRSIRLVGGTNSM
EGRVEVCSGGVWGTVCDDSWDNTDASVVCRQLGYGPGTAFGSAYFGQGTGTIVMDDVHCT
GTESYLTNCIHITNHNCGHSEDAGVRCGSCITGSIRLVNGLHDWEGRVEVCVQGLWGTVC
DDSWGSPDAAVVCKQLGWGTICTPGTVRLVNGANSNEGRVELCQNGKWGTVCDDSWDNTD
ASVVCRQLGIGTFGIALSNAYFGQGNVSILLDDVACDGTEQFLANCTYTSNHNCGHSEDA
GVMCLICIDGSIRLGGGTNSKEGRVEVCSGGVWGTVCDDLWDNTDASVVCRQLGYHSGTS
FGSAYFGQGTGSIVMDDVRCNGTESYLTNCTHTKSHNCGHSEDAGVRCTPCITGSVRLVG
GSHDWEGRVEVCVNESWGTVCDDLWDSTDAAVGTGSILLDDVQCTGTEQFLTNCTHLSTH
NCIHHEDAGVACHVCTPGTVRLVNGANSTEGRVELCHNGRWGTVCDDSWDNTDASVVCRQ
LGIGTFGTALSNAYFGQGIGSILLDDVACDGTEQFLANCTYTSNHNCGHSEDAGVMCPGC
IDGSIHLVGGTNSKEGRVEVCSGREWGTVCDDLWDNTDASVVCRQLGYDSGTSFGSAYFG
QGTGSIVMDDVRCVGTESYLTNCTHTVYHNCGHSEDAGVRCTLPASTPSDLIVTNETISS
LTLCWNALGSIDADGYVVNVTIGTSIIQTVQIEGGNSNKTRLEGLASGTIYSITIRAYQQ
LLGPASSAILGQTLQVCTAGSIRLVGGLHDWEGRVEVCVQGLWGTVCDDWWDSPDAAVVC
KQLGWGTSELIHCDSYLQYIIKGGTALSNAYFGQGTGSILLDNVECTGTEQFLTSCTHLS
THNCNHYEDAGVACHVCTPGTVRLVNGANSNEGRVELCHNGKWGTVCDDSWDNTDASVVC
RQLGLGTCELDNIMYCEYSSFLVGTXLSNAYFGQGNGLILLDDVACDGTEQFLANCSYTS
NHNCVHSEDAGVMCLVPPTVIGTNIDAVLKGSEVTITFNILRAFPLVSLDDIIWSFISIN
DAESRPILLNVTCAEMNEAICVDSKRYERYNFMFDHNLTTLTIQDVQVEDYGTFILSASN
SAGVGSYNHSLSVIVPALLGNVKGVVFNETKNQSQVINITMNCTADGIPKPGIIWFYNDA
ILNLKPRVTIVQQDLDRTIRDDVVHPDGTSVISILSISNANQRDDSGRYVCQAENIGGRS
LLTPPNDLIINVVPAKLLYTAGVSLEKERRQNVSFNCTADGIPMPTIVWRKDGHLIIPFH
KRSITVRSSTGFRSSIIPGVLQTTSILTISDLTGSDDGRYSCRADNKANIGTVLIKPFIL
TIVEHARSVWKRAFFGVTGRDFTELSLSTFNYVRGKSNGSTNTPMRSPHRKVSSEKSLST
RDSFSEGKTFYKKGGSGQSTQHECTKKKEEEVEGEIKELEVRAVDNDYAAIEDTGFDFET
DGQGTLRFYTKKYQDHVETQFDSLNEGKSTDEAKKEEVPLSKEGNGHKLHVNVPSLLQEE
EPVKKKEAISVKSFPDDDEDEKRRLREEEDKAYDAYHK

>PAC:15717362 pep:novel
scaffold:Aqu1:Contig13350:58244:75248:-1 gene:Aqu1.218834
transcript:PAC:15717362 description:""
MAMACLYLVAGVAFLLAGRTIAVAPVVAPLDGSQQVAVSPEPHNLTFVVAPPVFPSNISW
TYHNGLNTEIIDENTNPFLSFSPSRLTLTLSLPLSQNEGTYTLTATNEDGSSSAFITLDI
KSRPAFITLPSSQNKHERENVTFPCFATGDPTPSIEWIFNDTFVINSSNEKYQIGLFGES
NFGSLTVQDLEFPNTGVYICRASNQFGSLNATAVDLGVQVLPSVTSQPSDIAPVAGGVAY
LRCSVRGFPPVNVYWTKNITNVTELDSVVRYTTNSVYDHSSLTTHSTLTLNGSVLSTDDP
PATSVSDSTVRVNQSFSTTFNCSSFGIPPPSLFWYFNGSNLESGGNVVVTRTSFVNESDL

YIRSIELAIVNADRAAREGSYTCVAVNNVNNLISSPENVTTQVFVQVPPRLVLPNGPSVL
GVVGSSVTLRFQVEDASPDVLPERTFWSFQGTPLSSNLSDRFAYSMDRRSLAINDLTHQD
EGLYSVMVVNEAGTDSSEINLHIEAAPVLVSPFLTDSIVLQGNSTELRCSITSEPQPSVQ
WLFNSNPLNLSISRLSVGFFNGSEVREYFSVLSISEATMGDTGDYTCLGPNDTLSIRGFS
VSLSCQTFGSPTPNITWLKGSMAVSSSPSISITSIRNGFTRESVLTFLNLSFSDAEDYTC
LAQNDLVTLTSGNSTPAQLIVNQPPLLASSTDEFSIFNQSQTVNISCHFLGLPPPSISWS
RSDSADLSDSVKFTTFISSSEPPEGGVISSSTLQVTDVGESDTGNYTCTAANRATGPMEP
LNITSHSLRVLVQTSPLVNRVSAQTEIGVLTRNITLVFSVSRDVPAVVPIDIKWFLNGTE
ILSDDRHMFSDSRESLSIYNLTLPFDEGEYSVVASNIIGNGTESLFLDVESFVGNPARMI
GSFFSLILQRLSFKIISLCLCLFLFPVAPIITSNSGRVNTIEGNNATFHCLGVSEPVHFT
SWRFNGTDISPDSIKYSIIGNNTSNSTLIVHNVSLTDQGLYACHADNVHGVDSAVSVLFV
QVPPYISSPPSSSTVLVNTSAAFRCCSGGIPLPSIQWLKNGTIIDEANRTSFTIQESTYE
NCSVLNIANPVFSDRGEYACVSRNQLAEELMAINPPQVFRLSQATYTRNESDSFTIECTS
FGVPLPTLYWVPGPLNITNRIGDGDLFLTNGELASFLSSISGHTNRLVNLTDQCPMNRGP
SVCRGNVESSNDTNVECDRINGSLCPVSCGIDITSYNETRDDGRPVSVSRLTLCSLLKSE
ELSYTCVAVNNVTNDIGTPEAVSANLVVQVEPYVFSSQTDTQIAFRGHSTTLDFNISLAS
PDVPPEGITWSFTHRFLDISEIITGSGDHYEFSESHRSLTLFNLTTNDTGYYELTATNPA
GTDRHTIELSVQALPEIIRSTRSPVITVTDSVVPLDCFADGSPLQNVSWTFTNASGVLLF
EIRFVIDNDTARTGYTEYLPLVEGITRTYIMERFSISDNTVPSAMEYGRLSIRDINAFDA
GVYTCTLTNEYNLMEPDTYSVQVQVQLYPSISTHPQSTQCTSGDSCSLNCTVTGVPAPNI
TWYHDGTLVDDHSIQFTYTYNQTTSVLNIASVNYLTNSGRYHCVGNNTLYSPGSAESNEA
MLTVLLNITRHKCRVSSLYLVTNPSKVLSLPSHTIINETFTATLTCIVTGNPRPTVTWSI
GSRALASSNNGKYVITTNNSMGGLLFNVTSVLNISNVNKSDEAVYTCSSENEVRNNIGVN
RIAISRLTIQVPPTVVGMNKESVEKGSEIIISFNITRASPPVSLDDIIWSFTSINDVQSR
PMILNVTCAEMENATCVDSQRYQRYNFTFDHNLTTLTIQDAQVEDYGTFALSVSNPAVPA
LLGNSTGMIFNKTKTQLDEVINITMSCTADGIPKPGIIWFYNDAILNFKPRVAFVPQDLN
ETIRPDVIHPNGTAVISILTITNANARDDSGLYACQAENIGGRSLLDPPNNLIINVPVEN
YCESSPCQNGGTCNNLERGFYCICGRVGQAVYSGDTCEIRTESEIAPRFLSVTSVDIFVA
LFKPVTLRCSAEGLPNPSIRWYRDGVFVEGSLGPSPNEYVIQEIRLEDRGNYHCVASNAV
GVVASETVLVNINDVIEYIANLTVQVSSLREGVNITSAADTIVNTINSGIAGEEIGSNST
FFFIVRNGIPLNRTTNITVPIKITIRLQEDTPNDILRRNVEDRVSKFGHHLEISDNQGVR
RFNYCPPDNTDIPSPDGDPALRLIIEWPEILFGRVQNVSCPCDFQLSSTVLLATRNCGGN
LITGAAWERPNIQPCNFSVTTRQLCRLANLSSSELLNGLDNITGNIDKIDSAGVTVSATA
INSVVDDVISNDTLVNTTMNVFNNIQGVSRQELVIAQRTSDSSEKMLRSLFNITNTLPAN
TTILRDRMAIMTGSFLNSSISSSHNSTSNATVMLPDGFLRDNRDNPFVFSVFTSDSLFIP
NEDDGSTVGTPVLDFTLPDQDVSNLAEPIQLMFPNFTPPADGSRIPVCRFWDTENNEWSS
DGCSLVGGNICSCDHLTSFAILLDVAAEEPPTAAEAEVFQYLTYIGLTVSIICLIVTLIT
YLGTPKLRNSEASQLLIHLSFALLGLYFTFIIAAQGDRLTKPVCGIIGALVHYFFLAVFF
IMAAESVDLFIKLVIVLGPKIQRFILKTAIIGWIAPLFVIPITTFILTSTCGPSGFPLYI
GILLPFSVIYIFNYTIFFIIMISLIKKSASSKFSEAKKKNRKAEIKKQCRVAFTVSVLFG
LGWGFGVLASSAIGSTPVRIIFNSIFTIFTVFQGFFIFLLYVVLSPNARNIWKRWILRKE
ATKSTEASSSVGPSTSRTTQKTNASNNYGRKAGGIGGGKGTLYRNVYSAAKGKSSSPYIT
KDLISSEFASSQPVVEELKERLRFRSIDDEDEDRTFMNPLDDLGDVMSLVSDTQSLHETT
FSFPNPNPPSSQSQEEDDEERDDLGDIEEGPGKCSTFKNPLRQSLHRTTSRSSRPPDETE
LLSQSTSTDEPAQTVTLDLNAINGFTLSNGSSSLSQSQGLSGHDGSLLIRDEQVSEL

>PAC:15718102 pep:novel
scaffold:Aqu1:Contig13373:33197:34455:1 gene:Aqu1.219574
transcript:PAC:15718102 description:""
MESDNSSAEIPISEPPYCVGGHLNIIPGYIRVIAAITCALSILGAALIIILSYVFFKDLRN
KAREVIVHISAMDLLTVAANLFGIIINFDSHLYHEDPLVLPPHYETYNTLCKGQATIAVF
GNLSSVLWTIAIAVYFYICIMTDNKKTAKRSLYFFYLICYGLPLLIVVWFLPTGKLGYAP
VGGSGWCSFVIDSNPTEKLDSSKKLDSRDIITLFFGNSLWMYVSFIIIPFICISLLVYLR
LKTKKKEISNQDKLRMNMIEVKLLAVPIIFVFLRIWSELLGIITVYSNNKSCTLVTILLV
LGGIGDSGQGFFNALIFCLLNAKVRSAYFKCIKKSYKACKTWKCEKIEMKSRLIVNPDEI
DTDQASTQE

>PAC:15718113 pep:novel
scaffold:Aqu1:Contig13373:77074:78805:1 gene:Aqu1.219585
transcript:PAC:15718113 description:""
METSCNDSLLDGGNAYYDVFLKISIGTMCTLSAFGSSLIILTFILFKDIRTIPRQLLFNL
SIADLLTALSNFSGIFTNFDKYLNYCLVPLEDQNAGIQSACIAQAATGQLTTNSSILWTI
CIAFYMLIIIVFQKESIAKKLLPLYYILSWGLPFAVTVWFSFVGYLGYDPETTPGWCGIV
GSKLITDGNFTHKEKLVFPVVIGYTGFVYAAFFLLPIMYIVIRCHIKILFKRRPDGKMRI
HLQAESKSTDLKMIFIPLIFIFIRVWSLVVDSFDYYVDRDLKYKFKNSTVGAIFVLAKGL
GDSSQGFANAILFIGFTQAVRWKLWRPTQRAYKRYCRCCRHNRDELMMSRVTEATPLITD
DSTGTVYATENINDSCYEANKSFAQ

>PAC:15718116 pep:novel
scaffold:Aqu1:Contig13373:82903:84346:1 gene:Aqu1.219588
transcript:PAC:15718116 description:""
MISNNSDYPSWSINSDIDSAFYKNDPILRGVVIAACILSGVGALLVIFSYVCSRQLRSQA
RLIIILHLSFMDFGVALANFIGLSVYFNKYYLESYKNEGDFDDVTPVVRYSCMTQAIVAVY
CNNSSILWTLSVAIYLYFRIVTNPHSPEKFFRILLYIMYAVNYGLPLVLTIWLALTKRLG
FSPFDSSGWCSLITYRYIEMESYKPVKVDVYASFFGNDMLIILTLITIPVLYFSIKRNAK
KQLSSVATENLRHKLNSIELKFTAIPLVFLFLRLWSLILSIMYDYAQVDDKHVPQKLKII
LLYLSGIGDSSQGTANSIIFIFFTEKARSMLCALFCCFCTYFLKQTTVQSDMHEDSQEKE
PILTNSTSHQEYKAKGQTLYIIQSH

>PAC:15718143 pep:novel
scaffold:Aqu1:Contig13373:173182:177811:-1 gene:Aqu1.219615
transcript:PAC:15718143 description:""
MTRRSIHDVLFILLVLSLECRIYVSLSDGTNNTSCWTGGVQIPCATLDLAIQGAATLQYN
CSSGILINLSPGTYTLDTTSLLEQQLLRNNVSIIGMRDGSGYEEVSITCLHVSSSSYNWL
RHIAFQYVSLYNCNNIPVTCTEPNDCPISQDMYSYRFELNVSFTNLPLSCSSHQALSSDG
CKCESLKFTVSVIDEANNDVYDWKNDLYVCTDDTCSIAINNNSVYSLSSLCFNEEIINVT
MEKEVYIGTLGPINASINLTANVTVTIMVILIIILNIKLTNGLINGIVFYSQMVSIVYPE
VTTDVPSYWFLPNTFFNLDFTPFLGNYPLCIAPHMSPLGAISFWYVVGFYPLLLLLLLYV
WITLYDKGYKCVVLVTRPLHRCMARFWSMTGIEPSFTHSIASIYILCFTLLTIISIKILR
FNPTDRSLFFYDANQNPTDHLFFFYDANQKYFEGPHIAATLFSVLILLLFILLPTLYLLL
YPFKWFHKLLDWLHLRKQLLISLGDVFTGPYKNGTNGTYDYRFFAGGLIFGIILPIYIMS
MFGKKIFYYYKRRKDTLRVNTEEGDDNEPLVVDEDWIADRMENPQKYNERHVPVRLDDLT
RDTDEPTDEPTDEPIAIDKAATYGSWLSYPQIPKKMVGNTSTFPTVANVTVMAPFTPDKA
LRSVLGVTCCLSILGSLAVILTYFCFKSLRTTPRLVLVHLSFMDMGVGLANLVGLCVNFD
SYYFPHPSSPDSYEFGLPKLNNVSGVIHYSCIVQGFAAIYFTLGSFLWTISMAVYLYLRI
VHNQLPGAARRALFLCTIISYTLPIGVVMWEGLTRRLGYSPFSSEGWCGNQLIDLSTGKR
QVLMDFFGYQVWVLIIFIIVPVLYVSALSFICQELKKTRSQFGIARDLYKKALHKIDYKL
LLIPVAFLLLRIWSQIDVAIYVYIKTSLPTFWHGLFTYLSGIGDSGQGFVNAILFVVLTK
KVRAKLFPCCKRLRKEEDETSSIKKPLEGNSAKYGLQYKSTSRLNSFSPMS

>PAC:15719847 pep:novel
scaffold:Aqu1:Contig13420:79410:83987:-1 gene:Aqu1.221319
transcript:PAC:15719847 description:""
MMREAVLVFVLCVLSLSVAQDCPLPTKAEIEAVLPPLLVVSSGGQSYSPNVTKGSVQYVC
LAQGDIINTYKEVALIAIFTPNPKEPEQTNLFTLGCNSDDTLQVDTQVGQDNTNGLTSNE
IASIRLPQSIISIIENDRIDLAFTLYNKSVLFPIREPPPNTIVGSSVIGARIGGVSDGTK
LPDPVVINLALRRIANITNPRCVYWNFTTAGGRGNWSTDGCNTTVNATTNDLSFINCHCD
HLTNFACLVDVDVSATTNGATEAPQDSNLILKVVSIVGVCFSLVGLLLTIITFIIFKKLR
AREASKFHIQLCLSIIFGSIVFIVGIDRVSVRAGCITAGVLMHYFSLVSYIWMGAEALLM
FQKLVIVFTDITWKYLTAVSILCWTLPLLPVVITLAIDRDFYIFLYEKDENGNQSGFCFI
TEMIPFLAAFLLPIFIILIFNVIIFVLIIRVVILHTIGKKKRMNKSPLTTSDAIKMLIFY
FGVLILFGLTWFIAVLTYIIDPNIPYIIRLIFAFFNGFEGFFIFFFLVILSSDSRNAWKS
LLCPWTVRDGTASKVVKNKTSNIQVTLPQAKNEVSLVSINRKDKGKDFVENLSIESETIL
EDSGFSNDDDSMISKSGSIRFDCHFSTRRKHNVEKIEIDFTHGDDDNGDI

>PAC:15721507 pep:novel
scaffold:Aqu1:Contig13453:24876:27179:1 gene:Aqu1.222979
transcript:PAC:15721507 description:""
MLIFFAVFTVSWSQEQSNCQVLTDPSEYTTTSAGLVSFQGQTLPVIGNTTSGEPIVCPTQ
PQTYFPYSTAFQIPGAVFLPISIIANLLAIGLFELLRCLDVAKITTFDITVINFLVSTLL
FNAILLIHETFYQLILNTVSCEHIAFFSHLFTLAMFISYTFVGLELGRVSIQKELTVNKE
RLLFLLDFLASWILPFVVVASLVGVNFSVSNSVMYGIRKDGKLKCWINQGGVAVAFLAAP
IILTLFVIPIFIGVQVLGTTLRMKDKKSKKGQKEREDMMPRLAASLFALVNQRKKKKREA
PRLPIEITRKQQICYVLIYLVGYVAQILKAIGILVVTFTDINSTHIIITGQLLDAVLILA
FALAGKSIRLSLKDLFKSKVPKKIKKKKRRESSSSSSSSSSSSSEDNNDKVKPMSRGQSES
IEMKRTGELDARAQEGITASKPLPPTSRLEQAQFGERERQLQQQLLASLPGLVPQREQGV
MSQQPYLIQPTSSVLQQPLQVIAQVEPQPQQVVSPGVPQILQSVSPVQPYFIAPFPQNIT
QGQLLEPTPRVQQALPPKAFQPQTQTIQPLTILPPIQPQTQTIQPQTQTIQPQTQTIQLP
QAQTIQSQNIQSIPQTAQLQLQTIETQIQPQTSEAVIQLQAQDIQPQAPVRSQSIDELQS
ESLQGSKSIETQPQPQTATEEPNPTPSVPPTSLNTTTGPKLPNPDMEPAPAPPKLDFPPI
TKEPLIQNPFGTPLTELSRDAGIVSKGGKPITKASPARSSSLTNLTI

>PAC:15722211 pep:known
scaffold:Aqu1:Contig13465:76461:79843:1 gene:Aqu1.223683
transcript:PAC:15722211 description:""
MAQVQAGVRSMIMLLLVLVTVFSVSGQPSSPAKKPSTSVVTNSTAVEPTAVQTSPAPTSS
SSYFVETSTFFLSSTPTGTPAPGHARRRCTIFGFWKLPNVSNCYGGSIGNVGLQAGSFFG
NNNQTDVDGALYLVNQLNNLTRPRMPIPPDELNTLNYVVGQTVALLRRQGGPGASQAMDL
FDAFNNVLDRNNSAGWRELQQSQSSSEELLSNAEEYALYIASTINGTDDTTVFSAENLVL
RARKFSRSRFKKFSNSRFPNKSDLVNSSVSDITNSITIPADYLEARANDSDDGDVSVAYM
LFPSIQDFLPSDENYSIPSVVLSAQVITSDEDLTGDIETESVLLESPIEITFSLDLDIDY
NEDNESLVFNCVYWNFSSDFNTTASSRWESEGIDTSFANESFVRCLSSHLTNFAVLVSVV
DKESTTTIVTNGTDATETPTELEDHLLSVFSYIGCSVSIMCLLATIVCLLTLKKGLFEKP
VNFIHLNLSIALLLALILFVGGVETAVNIPELCKTIAVLAHYFFLSAFSWMLCEAIMLYL
LLVVVFSRLIKMWYIFLILGWGLPIIPVAVTLGVRFDEYTSEHLCWLNTKHGIIWSFVGP
ILAINTVILVIVLVQIARSMHAKKRRKTITKRFNNEPEKKNHSEAFKIAKGMIVLLPLLG
STWIIGIFFVSENTTVFAWLFVIFNSLQVWGRIQKRLKQKKRERESSMYTTKTHLPTKVS
TMSMTTENTVL

>PAC:15722288 pep:known
scaffold:Aqu1:Contig13466:38476:68570:-1 gene:Aqu1.223760
transcript:PAC:15722288 description:""
MAAQREQEEETLFNDEDAVSKLEALVFNSLSLGQWESARGHLISLSSHEKGRKTVKEILR
SIVLNPKAVWSESKSIPSENHLVLLALQAHAELCQDDVEGIPSAVQRQHEFKLLLHSCTL
CEGYTTQISEWLYGYYTTHCVGSTPHSLTPLHTFHATIQKFLLSVLQSNPSVAHVLISYL
TVANRPHYLTNNELLQLLYIDCIDSYLGRLEKVCRKASTTEDPNLHKDVAELSSKIHSIL
SLVDPLPDMGRFFKRLDSTLVKLIQLDFKNSKKEGLFTFSSLYSCFVGRSSPMLIQRLQD
IEEYLKGHSGGQVEEEVAKEYSSNEERWESELYKTLRSNHHFLERIMDAGLKAISSGSTD
RLADIFSDPMLTCIKPLLLLLGWDRYPDVGSGQKLLDVLWPEAQKAEPLLLSGCCKLAFQ
VGLVQWCLQKARPFLPVSPTPMTSSAGDVFQGLKNHSILYVLHHSTPLSSLDPYEVIDIL
KLRPDSSLNLPAMSPPFDIIMSPKQHSSSSTSLQPEQERDINTFRGFCSLKLIMEAVWYS
ILLSNGIQTFVSTGTTGEPKKEAGDGVLARPKSKVSRKLDLGQSDELSAGDDLVSSLSST
SNTSLYATEVTRRLEEAKSHLSAVFPLNFRLEVLENIFSLLFLVSDDIQSPEEGGSDHEA
TDPTSSIHSTSSPDNSSFSYSNSIAFIRKQHGFLLDERTSRDILLLLNDSIFELSAAKFS
LLSSPGKPMGSALPPGKEILSSIPPALLQQRFGKLQKDIYEAKWRLELVSSKTGLSSLSS
STLSSPESISSYESLSDTSEQEDDIEEHKKEPDLIRKGRPHDLFNEAGKRPTTPFKPSFP
LLAKEGSQIPRKTDEASPKKPTERYIRKSPSADEIYESSGHCADVEEGSPMPLAKKKKIR
SHSGSEFKIRKIKSQPVQKGTGIICQMLASPKSLLCKCLRHGNYLKAHEVVKMFGMENEI
GDSLVKFSEQLKEVRQELVSHTQASSLPSAAVTPQKQVVVNGSSGLQVAILNATDCSSTL
DSLNRLVLPTVLHKMLFAGNEDLDHSHKEMPLLNSLEENVPSLVILDLLISSKIDGHTSK
KIVNMAVEKSSVVLDCFSPKLSDNLVGGRRSFQERRSASLLDKPLQGPLSLLHTLSEVSG

YFLLSIPLAAPFPLSCQPIVSPHCLLTQFLYPIRVDLIQSWRDFSESYGEKRESVESVIA
KSCIDTDILDVLSKKESARGINPVFNELSIAMRSLPQDGDTPKAEGGESSVQYLLHVSGY
LSKLVHLLRNSLGLSSAQVSPSQMLSVLRESPTDLIGKLVFEKNINPERLEQFMTDLPHL
KIVEVIVKCCCPHIPNDSQRLSSYPLMNECIYPLNVINGEESVSPNGNGSEDENDPCLLS
HKLLGKMTTTLKGLCQTDGGKILALDLRNAYKMTEMKKVLSCTSKLASISLSSLKNEPNR
QIAFFCNVTNLLYCHAIMYIISVSDDISATGLSLASLESDKLAKIAYFSRVGYVIGELGL
VSLYDLHYSLLRQNLSLPVLDKERKVKLQPLKTIYEPWKEYAPSQPDPRVLYVIGTGSLS
SPPPRLMQVEHLHSDLESAEIKFLRATVSLDVQKSTVRIPKLLDDHRNDFISRKSRFEPD
NPYRHLSNIALLKYIKGKLLKEQEEQIGQILDLYENTSKRSKASHDLKIEVIDEDYTLGY
DFPRRGDGQSPLLPRSHSPGKKNRRLYSKIKHYTFTHETMYFVQSQAPLLASLVYLLCPP
ENMDDIGILNESSLTESIISKEEKEQGESSLVQSFMPRKAKIRQIPEVKRSLSLTDDKNP
VGSFLGWETTLEKILSLFRASSPLKKFLATRLVCFKGLLSWDKSAQECPDDQQEDDKSIN
LRKLSMLPGDSSDISQACSFVLRKLLRHGLISEGIAFLRNEPLICNSVVMQTVTDLVCSA
GLVHETKERESISQEAIDQPVLVDPLILIYLHSNKEFATRLVLSSLEIWPVSYCVNILMY
INYHLPPTSQLSAIVSKKLKQLYVYEKIIETVEVHNGQCSWGHWSHLSYDSNEKKDSILK
LLLKLKEFTLAREWNRVHHNSDDIIMQIEEQYIYELLEGMEEPDRYTAQSTLEDLPSNAM
KQQVCENLLVKCFNLESLILILRFLLDKLSFQIQSAEKLKNLNNQLLGVKALLCLPHFVQ
EQYRSLMKRPLFIVEQLLIDLKIEWAGLVIKELNEDEYICQMDTDDLPPLKYQTHTPEEP
LNPFTLLIMFYAEKALEFPLPSSREYPSRATSPSVPSTPFSRYDRMRSPSESPSIPPTPR
HKKGRTSSFSTVFPPSDRPKYSRFEPPPHPPKREDWKTSNYCELCNSSLFGMFSKRHHCR
RCGRMVCAQCSPHFMIVEGYGSQQKVCNDCYEYNFITSTTSDDPQLSDKEDDIVTPTQYP
WQLLPGDPHYNENLRTEFCYEESPSTTLCMVILDLLSDAKACAGFILHCCHNVSMQLVAD
DSGRVNEEIDHYFTINIMETMLLNAKLKLFHAGDSHGVELCDTYLSRVDLLRLLISSSCS
EIPSIQELTKPDLARRVRDRLVAEDRFELAMEVSTKCQLDSNTVWTAWGMGCIKAGEFQM
ARDKFRHCFHGGTQLSGGAFLIERIVEQILSCPLASMKNSDDWNDSMRLLVEHKQDSSFI
TDRKYEECLFYLKNYGTAAAMIGFLARHHYWKDACQQAYTKHCSNDVFVDQIWLPALKCG
QLDNLEEALTRIDPSLSVWEQYLTGVCRFLSQRKLFHVLYRIQLFMKDFVRAAMTCIKFF
IGFSGHKTTLSDLYGRKHYLETARNHFRTAIDARVKRSASFSTTELEGHMRTISLQMRVI
DQLHKAAATNNILNKPVPVTSMGDSGVQPSTLFGNGAVRGEVAAHAYRLPSSKVFTEVSK
QLAMKENYVGIRVLVQSIQKSHLMSPELNDEVCMAAIKVLATQTKEARTLDKHLDKLVSM
LIKDENKVNCLIQCGKLKNAYLVAIKAKLPEEVERIADAAARAGQTSVRDILTMVKPIGV
RLFLGEENITIANFSMIAAGLFHDIDVDSPDGNATNNPISADGTWYYPDGTTVPLGFGSP
LFANNTATGQIGLLRNGGIDIQGLYSCVIPNEEGINQTLYVAAYGNTDFIRNDELPVIDS
SSPSFQLLSSVDADPPVFSLSFNVTNRSPTIVTCSVDNGNQFNVSDNDLIHTAATVNNDV
QVQVLVTFRMRMSGLFKCSASTDRITTTPLVSTTMAMRNISVTGSPSNLTYSRDSLFSVT
LGWSPSAVISATPQYHVFVNNSNNVILSDSTLTLSLRPDDEYNIGLVATGEDLPGEIITV
TVSQDEKDIFLDTLEPTMISVNDSVVHTLILNFSSLKVSQAGDYVCGALLNDGAGTISIT
SNYSVSIQGFDFNHVDISINISSVPVAGNTFNLSCVILVPPNFVEDLISVRWTYDLEASQ
DVTSENNDATLVPVVRNGNIFTSVLTLDPVKTSDAGQYYCQTTINVFNYVDYTDIDLTVQ
TSPPSVSIVADPPTGPIYESTSYLLTCTATVNTTIVNTPVTASVVWTDPSGNVIPTNETR
RQVIPPTGNSLVSMLLFQPIDTGLNNDGGAYTCQMIINLNNSLITLSQPTNTTFDVAVES
LPLMTVNFSSVGSVEVGQNLTITCTITAVERLVVTPSITFIKTNNTDMEMLSDLNRPYTI
TTDGTGSVTNYTLILDPVRFEDSGMYTCMAEFNVTGFNNTYDSSTATYDYQEASHVFNLL
IKLPPFSVSIVADPPTGPIYESTSYFLTCTATVNTTIVDTPVTASVVWTDPSGNVIPTNE
TRRQVIPPTGNSLVSMLLFQPIDTGLSNDGGTYTCQMIINFSNSIVTLRQPTNATLDVTV
ESLPPMTVNFSSVGSVEVGQSLTITCTIITVERLVVTPWIAFMKMNDTDMEMLFNLNRPY
TITTDDTGSVINYTLILDPVRFEDDGMYTCMAEFNVTGFNNTNDPNTATYDYQEASDVFT
LIVNCTILPFVATSVTTIPGTTSANISFIIPKTSYAIEIYSISYTGQYFQATQAVSMTRM
SSSFVNKPITIMLTGLEEDNVYQFTVDSSNCLAPVASPVDCANITFLSYNVTLAWTAPAL
IDQNGAPVGYNLTCMNTKGVSVNGLSPTQTSMNTMFTITDVMPFTGYTCDLSFINVVGEG
PFTQCAFETAQDTPNDGPQNFVSSPTMTTVTFMWSKPSIPNGIITEYKLRVANVETNNDT
VRSIPVPPGQIDIMYTVDMKGFFSAYNNYTATVTASTVIGFGPISTTRGRTLPDMSSPPL
LNTRSFTISNTEFTTIESIDNHTINVTWFPPTTPNGQIMNYTIDVNEYSGAKLLRRTVTD
VNKDKFTDLIYNTMLTPGVPYTITLVAYNEFGRGMPIEVTVFTKILTPSVSPSGITVIRS
ADGKNVTITWNRITLKEARGFFEYTIRLSTKSSRKRQTGELAYRVPYTDTSYTATGLNGQ
AAYAVSMGLSVDDGLGQGPIAGPTSNPIEIASPDPYTCIMETTVSTDRGMFEWPVTALGS
TANVSCPNGPTGAVATRRCVTNSTWESPNITSCATTEVSREFRNISKINITTDNVVTVSE

NLTNLVVSTTDTADQNTDNIEVVSTILNQTARLLSDPMIVMSLSSSELSMTTENTVQILD
SIEEWSPNVLETESNNIINSFERIVDALINQDNFTNITIIEDDIALKGERFQQSEFSGIV
FTAVSSDNRLIVDTQVEDDDANGLKLNRIASIELPQSIINIVESDTIDLSFTMYNQSVLF
PVRDPPPNTIVGSSVIGARIGGVADGTKLIDPVVITLSLNRVANASNPRCVYWNFTIADG
RGNWSTDGCNTTLNTTDDLSFIACHCDHLTNFACLVDVSARVSEPTQPPQYVTVTLEVVS
IVGVCLSLVGLVLTIITLIIFKKLRTREASKFHIQLCLSLIFMLAVFVSGIDRVLVRAGC
ITVGVLIHYFALVAWMWMGAEALLMFQKLVIVFTDITWKYLVGISFICWTLPLIPVIITL
VIDADFYIYLYEPENESGFCFISDIIPFIAAFLLPVFVILIFNIIIYVIIIRVLILHTIR
KNKRMIQFFFAFFNAFQGFFIFFFFVILSSDSRDAWRSLLCPWTLEDKKKPQISTTVKSK
TLNSKTTSLQKKNAPRNLILSYGEKSMPDEKQAEFTDKLIFENEAIFEDEVFSIDDEFMM
SEFNNIRFDRQLSTRCKHLVEKIEIDFFDANEDD

>PAC:15722424 pep:known
scaffold:Aqu1:Contig13467:350814:366028:-1 gene:Aqu1.223896
transcript:PAC:15722424 description:""
IKSTQSYSASPSVETTASFAASPSIESTQSYSASPSVESTQSTSASTNTELTQSISASPS
VESTQSISASPSIERNQSFSASPNVESTPSIYASPSIKSTQLFSASPNVESTPSIYASPS
IKSTQSFSASPNVESTQSFSASPSVESTQSFSASLSVESTQSTSASTNTELTQSISASPS
IESTQSFSASTSTESTQSIPASPSIESTKSFSASPSVQSTQSIFASPNVESTQSTSASTI
TELTQSISASPSIESTQFISASPSVESTQSISASPSVESTKSTSASTNTELTQSISASPS
IESTQSFSASPSVESTPSISASPSIEPNQSFSASPNVESTPSIYASPSIKSTQSYSASPS
VETTASFGASPSIESTQSYSASPSVESTQSTSASTNTELTQSIFASPSVESTQSISASPS
IERNQSFSASPNVESTPSIYASPSIKSTQSFSASPNVESTQSFSASPSVESTQSFSASLS
VESTQSTSALTNTELTQSISASPSIESTQSFSASTSTELSQSIPASPSIESTKSFSASPS
VQSTQSIFASPNVESTQSTSASTNTELTQLISASSSIESTQSYSASPSVESTSSIYASQS
IQSTQSYSASPSVESTHSFSTSPSIESTQSTSASTNIELTQSISSSPSIESTQSFSASPN
VESTQSTSASTITELTQSISASPSIESTQFISASPSIESTQSFSTSIDTELTPSVKSTQS
FSPSPSIESTQSFSASPNFLLHQALNQLNHFLLHQVVKSTQSISPSPSIKSTQSFSVSPN
VESTQSTSASTNTELTQSISVSPSIESTQFISASTSIESTQSFSASTNTELSQSIPASPS
IESTESYSVSPSVESTQSTSASTNTELTQSISVSTSIESTQSFSASTNTELSQSIPASPS
IKSTQSYSASPSVEPNQSFSASPNVESTPSIHASRSIKSTQSFSASINSELPPSAKSTQS
IFASQSVESTQSFSASTDTELSQSISASPSIESTQSFSASPNVESTQSTSASSHTELSQS
ISASPSIESTQSYSASLSVESTPSIAASPSIESTQSYSASPSVESTQSYSASPSIESTQS
FSASPSVESTQSISASPSIEPNQSFSASPNVESTPSIHASPSIKSTQSFSASINSELPPS
AKSTQSIFASQSVESTQSFSASTNTELSQSISVSTSIESTQFISASPSIESTQSFFTSIN
TELTPSVKSTQSISSSPSIESTQSFSASPSVESTQSTSASTNTELTQSISASPSIESTQS
FSTSINTELTPSVKSTQSIPASPSIESTQSFSASPSVESTQSTSASSHTELSQSISASPS
IESTQSYSASPSIESTQSYSASLSVESTPSIAASPSIESTQSYSASPSVESTQSYSASPS
VESTQSFSASPSVESTQSISASPSIEPNQSFSASPNVESTPSIHASPSIKSTQSFSASIN
SELPPSAKSTQSIFASQSVESTQSFSASTNTELSQSISASPSIESTQSYPATLSVESSQS
TSASSHTELSQSISVSTSIESTQSFSTSINTELTPSAKSAQSTFASPSVESTQSFSASPS
VESTQSTSASTNTELTQSISASPSIESTQFISASPSVESTQSTSASTNTELTQSISASPS
IESTQSFSASPNVESTQSTSASTNTELSQSISASPSIESTQFISASPSVESTQSTSASTN
TEFTQSISASPSIESTQSYPATPSVESTQSTSASSHTELTQSISASPSIESTQSFSTSIN
TELTPSVKSAQSIFASPSVESTQSFSASPSVESTQSTSASTNTELTQSISASPSIESTQF
ISASPSVESTQSTSASTNTELTQSISASPSIESTQFISASPSVESSQSTSASSNTELSQS
ISASPSIESTQSFSASPNVESTQSTSASSHTELSQSISASPSIESTQSYPATPSVESSQS
TSASTNTELTQSISVSTSIESTQSFSTSINTELTPSVKSAQSIFASPSVESTQSFSASPS
VESTQSTSASTNTELTQSISASPSIESTQSFSASTNTESTQSISASPSIESTQSFSTSIN
TELTPSVKSIQSISSSPSIESTQFISASPSVESTQSTSASTNTEFTQSISASPSIESTQS
FSASPNVESTQSTSASSHTELSQSISASPSIESTQSYPATPSVESSQSTSASTNTELTQS
ISVSTSIESTQSFSTSINTELTPSVKSAQSIFASPSVESTQSFSASPSVESTQSTSASTN
TELTQSISASPSIESTQFISASPSVESTQSTSASTNTELSQSISASPSIESTQSYPATLS
VESSQSTSASSNTELSQSISASPSIESTQSFSASTNTESTQSFSASPSVESTQSTSASTN
TELTQSISASPSIESTQSFSASTNTESTQSISASPSIESTQSFSTSINTELTPSVKSIQS
ISSSPSIESTQSFSASPSVESTQSTSASTNTELTQSISASPSIESTQSFSASPNVESTQS
TSASTNTELTQSFSASPSIESTQSFSTSINTESTPSVKSTQSISASPSVESTQSFSASPS

VESTQSTSASTNTELSQSISASPSIESTQSFSASPNVESTQSTSASSHTELSQSISASPS
IESTQSYPATPSVESSQSTSASTNTELTQSISVSTSIESTQSFSTSINTELTPSVKSAQS
IFASPSVESTQSFSASPNVESTRSTSASSHTELSRSISVSTSIESTQSFSTSINTELTPS
VKSAQSIFASPSVESTQSFSASPSVESTQSTSASTNTELTQSISASPSIESTQFISASPS
VESTQSTSASTNTELTQSISASPSIESTQSFSASPNVESTQSTSASTNTESTQSISASPS
IESTQSFSTSINTELTPSVKSIQSISSSPSIESTQSFSASPSVESTQSTSASTNTELTQS
ISASPSIESTQSISASPSFESTQSTSASTNTEFTQSISASPSIESTQSFSASPYVESTQS
TSASSNTELSQSISASPSIESTQSFSTSINTELTPSVKSTQSIFASPSVESTQSISASPS
VESTQSFSASTNTESTQSISASPSIESTQSFSTSINTELTPSVKSTQSISSSPSIESTQS
FSASPSVESTQSISASPSIEPNQSYSASPSIESTQFISASPSFESSKSTSASTNTESTQS
FSASPSVESTQLTSASTNTELNPSISASPSFESTQSTSASTNTESTHSISASPSVELTQS
TSASTITESTQSTSASTNTESIQSISTLPSVESTQSISSSPTVELMLTKSISTHMSMYIL
SSSIPSPSPPGTCEKSTTVSDHGSFVWLETSVDETGNVFCPHGPPGAFATRLCMSDGNWG
LPNVTNCANPKITAAFTNLAEANVTASNVASVSQNLSTLVSQVTEPGDQNTNNLVNISSL
LNKTASLFANPIIVFELSTEDVESTTESTVQVLNSIQNWPPHIVEAQSNVIVQSFERILD
ALMNQENFTNLTVIETGIAFQGLRIKKQDFIGITFIGSSISGIRLSVDSIFENNRSTHLN
ELDEIRFHFPSSIINVTKDADINVVFVMYNETTLFPLRESDPDTVVGTAVTGAKVGGIPD
GTALPDNVTMNFTIREENATNHHCVYWNFSAAGGKGNWTLAGCTTYVINTSFVTCVCNHL
TNFACLVDIGSREGNTPPPKIRAALNIFSIIGVFVSLLGLLLTIITLIIIKKFRERDVSK
FHIQLCLSLIFMLIVFVAGIDRVSVRVGCVTVGVLIHYFALVSWMWMGAEAVMMFQKLVI
VFSNITWKYILFVSIICWAVPLLPLTITVSIDPDFYIYGNASNNEYSFCFIGELMPFLTA
FLIPVFLILLFNLVIFVIIIIVVIKHNLKKNKRLGKSMKTKDAIKMLIPLAGVMLLFGLT
WIFGVFTFISEPGVSNTVHSESRDAWRAFFYREKRNISSNKTAVIGSSQVNTNRYKTRVS
SLPQSNSLSDTVNSTSIDCSRRDNDLSEIEAKAE

>PAC:15723157 pep:novel
scaffold:Aqu1:Contig13478:381567:385085:-1 gene:Aqu1.224629
transcript:PAC:15723157 description:""
MYVINYYYYSADPPGVNIPPKLKRIEVKYDKELLTVHGPFPMETVQNIPAKLFSNLTMIC
YSEGIPQPETTWYKDGQPLTCNKRFFQILEVQQTDRGYYQCKVKSTSESQESGEFLVNIT
GVLAFRIRIEAEEQSNATVIVKMIRNNIINTRVGDGIVISVELLEQPTKRRKRYNRNPPM
ELTIVVTLFFEEEAPESQDAIRDEARESLKSEIENEGYRVVNISRFDGCPRNNTYINGTE
LEWPELNIGDTAIISCPCGGLDLRSTGLIASHKCGGTFETGGQWEDLKDEKCIFSETTKA
ICELAGKPSDEKVEQLGSLTEDVTNFEPAEVITATVVLLTTGQDAKGNFNLSKGFLNVVN
NIASVNVTVLRTSQEEARVTTKLMDSLHNVSEGLPIESNDKPAILLEELFVVLVQGVDQR
SFANNTFVANLRNNSLVNDSLELVDNSNGEYSNSTAFLTLPPNLFDNLALSNVSNLADKL
VSIAFTDETLFLRRTSAIENKKVGSVIFSVSVANQSRIDNLTNPTVDMSFQIIKKPQAEF
AVTCVYWDAKLDEGYGDWSTEGCIVNETEYLNDSSRVLCHCNHLTSFAIILDLTPMETPT
DGPDHYSYTLSAITYIGTIVSTVCLLITIVTFLSVNSLQIYRKLRRTEHGQLLIHLSVAL
IGLYGIFIGSSLSIKIHVLCSMTAILLQYFFLVIFMLMAALAVDLYLKLVVVLGGKISYY
VLKATIVSWVLPIFITLFSFAPDHSNFYKRNYFCRPYDIPFYVGMIAPFAVIYLFNWIIF
FIILVRLLLRRDVSPQEGQSKYKKIKQQLIAAIGLSVLLGLGWGMGIPASSQFFEKTPAV
QKTFEVLFVSLTSFQGLAVFVMQCLRSLEARTIWSTWYHGTLKYSLQSSNNARLTISSDK
GQTKVRETGSTFPETRLNGNEQIIDGVEGQQIELISQSPSDESNGQAGDTTETIMNPGFI

>PAC:15724467 pep:novel
scaffold:Aqu1:Contig13492:436788:447051:-1 gene:Aqu1.225939
transcript:PAC:15724467 description:""
MAAALLLFIFLVSSSLLDLSASQDCNLSNEEKFNCSSLDDTTHPSIIYQDSPFTIEIYRT
NASLLISNASLFISTNGYCGPHSFASGVQCSVVSTEGVSNSTITCSNGFHVGPAVLIIGL
NDCALSLYVQWGQLPTLSIQENGEDIDVLGNIYVVHDQATPFNVVCSVSPPSELFLPVLN
ARKLDGNSPIEGLNIYQGGDRITASFGEGFSLDFSGVYTCSSSFNASTILFIVTAQDIPS
VYYPNGNTVSSVILGSPLSIKAAILTERPIDTDSVQFMSLNSVPTTGSTPTNLPTIDPSS
SPMYSTGPSPVVQLIEDDEYDQSLYITFDAVGQGSAGLYNVCFSYFDANSSSLGSGSDTE
AVNPEVVCVGTFNISSISVPSVSSLNNTLVVKRSSAFTNGSTTLTCIPSNPSAVIRWQYF
NGVPLWEQYSSYLFSPPGLNHTLTLTSPPYGILLIECGLYDPGLVRLVNGIELTIVSVSR
PVSIWNNNNELFSDAIINSFRVYFPLNTPFLSLRCFAEEGHGQLQWSTRDVSSLPETLLT

SDTAPDLSVIGAGQDLTINYANFDVNDTGYYVCSSGASGESFEVYTTLSNPYFNATAPSF
IRAPIGTNVSLVMRYAYGSEGFSNNGQSFLLSIRIQPSDTKFSSFYAVSNITLPNLSYRF
NYTFIVGVIHTGIATFEAPVFSPLPPSTVYALSGRRVNISCSPNETAAPVYWTIEIFNYE
ILSSLLDVPEFDLSPPGLNHMLEFNLTSPTFSSAFDVDLASISQMTFTCGFRDGANFGIY
NPRTIRVDIEQERIFITNFRFNVANNSIVNYFPQYYFNSKRSVSDGFFASFSCTGVQGDH
YPVFKVELFNETGFIRTVNASSPNNEITASPNGEFQNTLHVYVHDETSGRYYCESSVSGA
FKSFIVTSENPFVRAASSKVINAFLGESVTLSFLAAEYSRGGNNYLKAIPVMFTSNNGTV
INYYSYQSCEDDPCYYYSLSLRSVSPRSAGQYTAYLDNDYEYEYDYSAEVPNDTITVNVE
VPQLLTSGPVVQALLDTQIPLSCKPSVGSFLITWQHNGQPLNCETEEGIPTCQFTNNQQD
TLLLNRLSYNDAGTYTCTLVLNGEPIVSQSIALGIIQNCPTNFSNGILWAVTPSGSVARV
PCTAAGPQFQSGLFATRVCSDASRWEAPDMTTCLLREESNNVASVWVTVDTSNATTTPND
SVLVEVLQDLLVNYNVSYKSVSLQSTYTETGSALLEFRVELTGFNDTTPAQLNQLVASSA
QFGGLYVLPQRSGVQLFISASSCPCLPKDTNTSSVTYLCYEGATTPCNCNNGVCQCVPPF
VGDGRLCTVDSDGDSFPDEPLTSCAPNSTLHYCIQDNCRRTFNPNQDLAACQILENFEGC
GFGIDTEWGIAWANVLVGEVDRQPCPGDAATLGTATKACLSVGVWGETNVLDCLPVEYQN
YLDQVESILDANITIDEQLSLLSELVSTLATSTSEVVESAELFLPGSLNATNKIVDTVID
VLFDSLDEENDNISRIVDGLNLVQVFNSLLDSSQLAAWQQLQQAPQDTTLRGSTELLSNA
ERYGKYLALSRNETTLRRRPNIVINANQYSAASESLVFPQPGDTDGTLFTSERGAEVSIP
PELLRERRNRGDTTIPVVSILFKNLENFLEADGSSFNNTRPGSIVWSGQVAGIARNTEFP
ISLRFNVDNFTNSSGYKCVYWNTTRREWSIDGVNTVSSVVNSTTGRATVYCQSFHLTAFA
VLVDVSGSITETGSTEYTALKVVSYIGCAISIACLIVTVVFFLIQGKALLRSVHFFIHLN
LALSLLCGYFIFAVGVETARNSSGGCAFVAALLQYFFLSSFCWMLCEGVMLYLMLVKVFS
TLSKRWYFFLILGWAPPLLPVVIGLAAGHSNYGVKNDEGDLLYCWLSVDNGVIWAFVAPM
IVIIIINSVFLVLALYSIWKTRSNQKSIKKKTGANRDLALSLLKASLVLLPLLGLTWLFG
LLAVNRNTSAFAWIFTLLNTLQGAAIFFFHVVRSDKVWSKLSPRINKVKRGLTSRTRTYN
YISTSSTMKASKDSQSSISMGTIKKSVVENDYSTASSLGFANTSVEDGDDEKIELAQLEE
KKMPTEEKVEEMSLSERAKEKEKEALGSD

>PAC:15724646 pep:novel
scaffold:Aqu1:Contig13495:263088:265060:-1 gene:Aqu1.226118
transcript:PAC:15724646 description:""
MSSWKTPEIELTSAEVLCPSQYSINCSGSNEDSQYNLYLGPTPTYVSLVSCALSCLGSLS
IILSYVFFKELRSGSRAIITFLSIADLVTSMGYMAGDINYLTHLSSANGSGDCNTFDTIC
SIQSYITTWSQLSSYSWNSALAIYLFLSISFAKNRAASKMIPYCHIISWGLPILIAFPLL
LFNSLGFSLYSASNWCFVKDPMVSYAKKSWLNENVMTPLLIARVVPEFLTYVLIIILYSI
IKIFVFKMKRRVKAESSTISGALIQNSLNKIDNKLLMIPMAFILLRIWGFIQIFFILSVS
KYFDKGCVIYPVYIIHFVLGILEAIGEGGQGWANALLFIIFNSKTRSLICASTFKLCFKH
CPCFRFCCRFTCCLKVLYKKRRNMIFDKTEEEEENNSILSSESTIGEHSTGLQEVSADSC
RLSIQYAD

>PAC:15724648 pep:known
scaffold:Aqu1:Contig13495:267115:268872:-1 gene:Aqu1.226120
transcript:PAC:15724648 description:""
MWNTFHELMKQSIYCPRPNRTCSGDYSSLYYGDTPAIVSFISCSLSLLGSLITVLPYLLW
KDTRTGIRRIITFLAVADFFTAASYIMGNINFYIYKHNSKTAGINTACNYFDQICEVQSY
ISSWASYSSFWWTSILALYLYWTVVKGDIKKGERYFPLYHVLSWGSPMLAMVPLLVTNSL
GYSYVAAAGWCFIRGSRFDSSLQYAQLDFEDIVKILAGGKAFEIGTYAWVLLMYGAIHCN
IRKKKKDQTPTSINGGVSVLLRQIQKKLYFIPAAFIILRMWGTIQFIVSIFVFNFRKDGC
IEKGYRDLYFSLAILQCIGDGAQGWVNCILYIFWSPKIRQRLILNPLSNCCYHVATQILP
TTSRHSSALSPVNSPSPKSVFVSVQHVGEEDGGPKENRGGVEGEQEGTSALLQTFSREED
EERVPLIASEKT

>PAC:15724653 pep:novel
scaffold:Aqu1:Contig13495:275989:277619:1 gene:Aqu1.226125
transcript:PAC:15724653 description:""
MTNCTIEFVSSFLTLASSSLSIFGSCLIILTFILWKDVRSSTARIIVFFLAIADLGTGLS
FFVSSAGYISYYSEDDLLINSSYNSFCSIMSYFTTFFPVSSFFWTAYLALYFVIALVFKK

PRWSGKLIILFNLTAWSIPFAICTTAIAFGVLGNSDSRTSGGWCFVTFHGAGRFNNYSYG
TYLWLEAVCGKGWELLFYVVVVFCYSTIYWVNRKLCCKSSNANKIQSAYPPLIEKESDSY
IRVSQRASSQAALSQAISKKLFIIPVVFVFLRAPGTYRYFLSMDPYHCRDSDDDYDNFTS
NYCGVTEKCFRFGGNYYILPLQAFCDPLQGFGNAVLFVFFSRVIMRRLSYSIHKKFHLIK
YHCTRFCNGEEATDDAPTASLKTKNSPAIVRAQVQQSSEYDTQLDDDEYHTSLGSVSPHM
QYGSVVSDNDTITNSINQ

>PAC:15724897 pep:novel
scaffold:Aqu1:Contig13499:83135:86726:-1 gene:Aqu1.226369
transcript:PAC:15724897 description:""
MAAFLLLLFFSTASATAKSCIEGPSNYTIMPPSSEIVCSNESSCLDLNQTCININSSSLT
AKKFNYHLGMLFENAVKSDSCSELNVLGFVYICCSETGASLSTCTYSFIEHCVNHTAIGR
AQVLSCVDKFYGFCENGSNPSCREAIEDVIDDVLKNRFDSPVGEEILASILEKENGLRKF
NVIKRVVSLLRGKSQSAKHAQKFLKEAEKVIEAFENDIAKSSSMIDYEDVKNLLYALDNA
FNGSHISLLSVSDAITTLYSNDEYSGPNLLSKLKRLSKIFGRAILYGSGRIKSHFNFSGN
NLDVKFRVIDKEMITFEQIYESENGSKSVFIEVPKNALCSQSQLQLSTDDNYTYTFDEID
DKDDIPDNRSTLVSLVVSVQAISDNTVNISDTVQLMDNVSLIFNITEFESNEEPFCCYWN
DSLQNWSTDGMTTTILSSSQPSAEDTGLEIISYIGVSISIICLILSIILLVALRKQLQKG
PLLYVHINLSLALLLALLVFVFGIELPRSIPWLCSVVAGLLHYLFLCVFCWSLAEGIMLY
ILIVRVYGSLADRWYLLLPLGWGLPIIIVGISTGIKHDLYGTDDHCWLSTSKGMIWSFIG
PMLLIIIINTIFLIMTLYHIIKSRMTGGAEYNLKKIDFIKSSVKSVVVLLPLLSITWIIG
VFTFNEQTKLFQWLFTIFNSIQGCAVLYFHVLRNKFITNWVKGRLRNKYKIKKQEPITSS
ATSSPL

>PAC:15724932 pep:known
scaffold:Aqu1:Contig13499:262316:269156:-1 gene:Aqu1.226404
transcript:PAC:15724932 description:""
MAATTSVIIPPPLLSLLLLLLVNQSQLSIASRLPPCTTFRNTCSKVFQLDLSAINSTDEA
VSFSISSSSLALLNISYIVNTSRGDCLTRSGECQNVSEQYYSNGVSSSQEQEVTNVYYIS
CTIKETEPLYSVISVKLDFGNCSYTFGLLQDGGDGGNLCPADYDNGLLWQQSWPSLLPVF
KSCNDISERFSSNSYAYRNCTSNGIWTSTDISHCRLKPSYAQNAFFNIWYKFNPKEPFNK
ENYLISNMTIHLRSVDYSNISLQYVTRNGLLLRAGFTVILESNLSSPGERADITMLVQNL
KELKILSIYETNTTQAQIFRPNTSCLCDMEYRNSLTLPVCVGDFGSPCNCSNGNCKCSSP
RYVGDGYYCTVDSDRDGFPDVPLDSCIENTSKYCRKDNCPYIHNPSQTNYSCFGCAAERD
RIWNLTWPDTQFGKTALQKCPNSTTGGIFFASRKCNASLGWLWPRTSNCSLSNWFYFKLF
NDTNYAVNHNLTDEERVAELNYLYGELIDSYSAPPSGSNGGPTIGQPDLKKSPYSLLHIN
KMVDYTLKLLPSAEFDEYNSYTFIPDMVDVFDDLLNEDYAEEWKEVEETEGHHFGSTLLL
ENVENYFLYYTGFEKSVRIKRKNFNIMSDVYNLSSQVAEGDYVINSNLSDSYITLHIPEG
LLREKTANGIEPVSIISIQFRSLDDFLSSDGNNTNDTKISSNVWSGQIGREGTVFSEPIL
ISFTAEKIDNETHNYVCSYWNTTSGQWMTDGVYTVGYYEWTDNRSYVVCSSYHLTAFAVL
VSVNGNNGGGSNEEGGTTDDSGDDDDEHGSSLTSLNHKAMSIATYAGTAASVLCLALSFL
FFIAQYKDLLEKPHLFIHLNLIIALMLAYSIFGLGIELAKGNEVLCSVVAGLLHYFFLAS
FCWMLCEGIMLYLLLVVVFSRLAKRWYFFFAIGWVTPLIPVSITMPVHYDKYTVKDSFGN
ITLCWLSDESGTIWAFVAPMLLIIAINVVFFVLIVRSLCKSKRMEKSKKMKATNLRQQTV
SLLKNCIILLPLMGFTWLFGLLAFDTDTYSLIFAWLFNILNSTQGVAIFFLYVVRNDKVW
SKLTFCFRKYISKEVSSPSNTMMKPSLKSTRTLISNVSLSQANTFGENSSQDGNSSTSDP
PADNALFTLSETDLTATHKIEDTKDNVSTASIKIDFD

>PAC:15724934 pep:novel
scaffold:Aqu1:Contig13499:279323:283113:-1 gene:Aqu1.226406
transcript:PAC:15724934 description:""
MDPSAINRLTSFSVILLGLLIPFLLLDSTVGLEACSSKRPDFTLQAPFHSGCTGLELPNC
IRTSIQSCRSNGSCLVQKTLNITRMMSLQVGRKYLLETFVENVINLTKRPENMNKNIRTG
IMREYTYVCHKVFNESLQDCFAEFIAVCLDLPSHRSPSPCINLLYNHCLNRTKDCREAVE
NEIEEIIEEECDEDYDDCEELIDDTFNNQFRFNSMKRIGTALRVSSNSKTKNATRLFEKR
RKVIRELVGNINNVSSIMDQEDVQILYEVMDSSFNISHVSSLSLSLSDKIGPMLLTNLKE
MNIVFGKAILSGGGRIKNDYKFNGNNLVILSLFYDEDEGLSVKINLPRTALCQSMGMKMN

KRNNKKDDDDDEEDYDEDVPDRYSNCFTPQFPSISNDTTDVNGMFDLYENISITFNVSKF
NNTNPMCHYWNPSLQNWSTDGMTTTMLSSSQVLCTSNHLTSFAVLVDHSGVTSENDDPSI
SPAEQIGFQVVGYIGCIISIIALLFAIVLLALLRKQLQKGPLLYVHINLSLALLLALLVF
VFGIELPRSIPWLCSVLAGSLHYLFLCVFCWSLAEGIMLYILIVRVYGSLADRWYLLLPL
GWGLPIIIVGISTGIKHDLYGTNDYCWLSTSKGMIWSFIGPMLFIIIINIILLAMTIYQI
MHSKSFKAATGADPNLKKIDLVKTGLKGVLVLSPLLGITWVIGIFAFSQQTTIFLWLFTI
LNSLQISDWVKKKLSHCLRRTKEKSLSSNSTNETFMKNSSIESKA

>PAC:15726423 pep:novel
scaffold:Aqu1:Contig13510:653267:664006:-1 gene:Aqu1.227895
transcript:PAC:15726423 description:""
MLPEGQPIPLNISYDFVNGGPRGENQTIASLHFKQDGSNLLSCSSTGCFIHDSPLNVTKS
LDSNINNIQADITDDRPINISRVFVVELDVVNMGNLYDTVTATFTVSMTSTFTVSMTPTP
TNLVACSMETIVSSDRGTFEWPETVSDSTVHISCPNGPSGATANRACTNNGAWESPNVTL
CRAITLISNQFRNISKVNVTTENVVSVAENLTNLVVSTTDAADQNTDNIRTVSAILDQTA
ILLSDPMIIMNVSSSELSMITKNTVQILDSLEEWAPAVVEIESNNVINSFERIIDALINQ
DNFTNIMVVKNGIALAGESFQQAVFNGTEFTAASIDDTLEVDTQVGQDSTNGLTSNEIAS
IRLPQSIISIIETDTIDVAFTLYNKSVLFPIREPPPNTIVGSSVIGARIGKLTDGTKLPD
PVVINLVLNTTGNITNPRCVYWNFTTAGGRGNWSTDGCNTTVNATANDLSVINCHCDHLT
NFACLVDVSARTNGATEAPQYFTMTLEVVSIYGVCLSLVGLILTIITLIIFEKLRAREAS
TFHIQLCLSLIYMLIIFVVGINKVSVRAGCITVGVLIHYFALVSWMWMGAEALLMFQKLV
IVFTDITQKYLIAVSILCWTLPLLPVVVTLAIDRDFYIYLYENGNQSGFCFISELIPFLV
AFLLPVFIILIFNVIIYVLVIRVAILHTVGKNKRMNKSPFTKSDRSSSAYSDTDSGSGFG
GGSGANPFGDDDKELLVEREDPGLSPEAQLQLQHDTSEVEERERHMRQLETEILDINDIF
RDLGTMVHDQGEIIDNIEANVEIAGTRVESGNKQLGRAVKHKRCSRRLTVCILCILLAVA
IAIVITILILVGVLNGFKK

>PAC:15726767 pep:novel
scaffold:Aqu1:Contig13513:266519:268222:-1 gene:Aqu1.228239
transcript:PAC:15726767 description:""
MLLFLSTTLLHRTLYDTRWSNITSNGHSSDDNALIVRIFACCMSSLSIFGALLIMLSYLC
FKNIRTKAREVLFHLSVADFGVGSTNLIGAIVNYDDLIDKCRNQSSSVLFSCPTYVSLCK
TQAFFAQFFTVSSILWTLLLALYVYTLVLDSSRKLSLWIVRFGYLVCWGMPLGLSIWFVL
TRKLGKTKIGGAGWCSLRAENKEGNVNHFAVFFGNDIWVMSTFVLILVLYTTLHCHLKDR
MKEMKKYNSDTFSQHSDAKFLAIPFFFLLLHAGTSENMSHTLQHIVLYWMFIGDSSEGLV
NGVIFCLLTPRIRQKLLSLCCCCSCYKFKNSNPSSIVRFPRETMEQRRLLENPDSNASPK
YSVNRNMSSSASSITSSSDSEY

>PAC:15728257 pep:novel
scaffold:Aqu1:Contig13521:116118:122705:-1 gene:Aqu1.229729
transcript:PAC:15728257 description:""
MCVATGSTLNGGQWVRAIDGNPVICHSNSDTDPFRCDSLASPNASLSLYLPNGQALLPER
EGFYKCCLPTGCLDPNTNVITANIFRWAQIAGIKFELLSDMTMLPQQYALHAIKIGRMNH
TFLLAATWYYETGDTSSNLTSFGISHENVVRNRYYTVTVPASTPSNLSVTSGTASTISLS
WNALGSIDADGYVVNVTIGTSIIQTVQIEGGNSNKTTLKGLASGTIYSITIRAYQQLLGP
ASSAILGQTLPVVGVAVVKKSGQSISFNCTANGIPTPTIVWMKDGQLIVPNKKRSISVQL
SAGFHSNNSSNISQVTSNLTISDLTGSDNGSYFCRADNNANIGTVLNTSYQLAVIESPLT
NFCLSSPCVNGDCQSLSDAYYCLCSETHTGKNCQEKVTIKIKPKITEPPQTTTGELYSPV
NLTCKATGSPFPLILWYKDKKLFVNRNSNPSILVFAELTLNDRGIYHCEASNIIDGRKMS
ANSSSVVLNITNVVQYKAEMHLSTNSYVGTNDSMENTKQLMIQSNNYLSGSNISDTSLFY
IKIQLNPDELSLGTNNDSLLVVVTLVTQKGQGDKILLNGVRHVLNFIQQFALPTFVTTVE
RFDGCPSNFTIIPSHSGNPNLTITIVWPETNIGVLAVVDCPCGTNGTSGGGKLQATRYCG
GDFTNGAVWDAPDVMRCNFSDLARTLCHLKDLPVEERINELEALTSDSSALGPTEVAASI
SALVSATGELEGNITLTTTFLDTVDNILVVNQEVLQKSQESSNTSSRILDAIENVVGDIN
ITNSSEPVVIARNHFAVLVQQIDLKELNESSQVFSVNIGDFATQNISWDDLSFGSNSVSP
PTGSIQLPSNLLSSLSNNLSDDSKIAYAVFVTDSLFLRRKINYWTVGSIIISANVVGIGP
VKGLKSPVNLTFQLNPDINGSFPQCSFWKQSLDNGYGDWSNDGCNTSVDPKYVTCHCDHL

TNFAVLLDVSLNNEPTERTELTLFLDSISYVGIMVSIVCLIITIISYLLSKKLRSSDHGQ
LLLNLCFALLGLYLSFIVALHSKNINIFCAFSGAVLQYFFLVTFIVMAAEAIHLYIKLVI
VLGRKIENYVLKATVVSWIAPVFVVLFCFSPDYKSYISDPPNFCRAFRAPFYIGMVVPFV
IIYLFNCIIFVVIMVSLLHKTHSLKLNDAKMKKDKDSKSFLKQQLIRAITLSILFGLGWG
LGLLVTEDIYTSETLRDLIASVFVILTGFHGLFLLITYCLRSEEARHVWKNILFCGKDKE
FSKLSLSNLNKTWKKSPNTTKISYNLATSLKKDQAEEGSQMRSYEYTKKNETGKKEVHKD
EGNFDLNNPNDGQATLRFYAKKYNDNEAQFDILNDESSESEVEMDEKNCEKYHQNKHC

>PAC:15728351 pep:novel
scaffold:Aqu1:Contig13521:509315:513851:1 gene:Aqu1.229823
transcript:PAC:15728351 description:""
MCGSDKYDRVGAFRRCIDVTTPFPPFDITKPTCTLSTAQLQEIVANSRYQQQYGFGCDDL
IRFADYQQLNNVNLLELSSISLNSTHSKSLATAAFLCHLSATQRLCDHYNLCFSYRENAT
KAYERNACIMDFSRLVSHGSDRVFNEIIYKYLLKPSLVESYSQAGRFNLAITQDYLYTCT
SLFHRNDIEDCIIQLLNHCFLFEDEAHYLCYNGIQDNFCSQFGGDNQLRCYILLEEKILT
SIVDEKPIGDLNSTDIDEVKAIVAGSSRRANYTNETLTQLNRIVDYFTLNESVVSQNIDK
QDANTFLSVISTLASKEESLALPENNETNSSTALLNDLAVMSKLIGSAVLTSNDQVEGNF
AYSDNNIGLFLTVGSVEYLSGALKLNSTNSSAEINIPPASVCGFDYELSKNDGSYEYDEY
PKICQGRPSCNISQNGFTYTCYDMYTIFGECSKRIAIPRSCPSVVSTSIAKLSLPGTLPP
NMTIASNVINVQIVTRNEDSSVSNVHSLKDNASLHFKLAEPIKENFIPKCHFWNTTENVW
SDVGIETLVLSDVDILCNTSHLTSFAVLVDHQGLIESASSSTAHNGTSIIPSTELQALSI
VGYIGPSISLLCLIIALVIMIYLRKEMNVNFLYHVHLNLCISLLLGLTVFLFGLETAIHY
DWLCSIVAVLLHYLFLCVFAWMLAEGITLFITVVHVWGMKLLKWQISLLTAWGIPLFIVA
VSFGIRHDLYGTDNYCWLSSQKGLVWAFLGPALAIAVINTIFLMVTMIQLVRVIRNKFDH
RKNSKNLKTVKSAIFGAIVLFPLLGITWIIGVFAVSQNTTVFLWLFTICNSLQGVFILIF
HVLRHKTVLTWIVRKYPCMDKWIEVHTSSEVIRRMSAMKLSVGKLAPQDMLSAKFGLRRN
SSYSLSDLIVSTSNENCINSELDTIAEEGDDEGVA

>PAC:15728352 pep:novel
scaffold:Aqu1:Contig13521:513988:518699:1 gene:Aqu1.229824
transcript:PAC:15728352 description:""
MLLSSLLFLLFIATAHSVYKDGTGDERLRAFRRCVADNDLPQLNTSKPRCPVTNTQLLNI
AANSRYQQDYGFGCDDLMRFADYAESQNLDLLDHCFALEDVYYYYSYWCYIGVMNRLCVQ
FRGDERLRCRILIETRLEVSLENQCANENNEGKCEKILTSIVDEKPIADLNFTDIDQVKA
VVAGSSRRANYTNETLTQLNRIVDYFTLNESVVSQNIDKQDANTFLSVVSTLASDEGTRN
LRENSTNNSGAMLLRDLDKISKVIGSAVVTENEQIEGNYAYNDENIGFFLALGSSDTNYS
SGGNIDNHIYILLFIVMNLALKLNKTDTTSPYMTAEISISATSICGFATTDESIGSGDDF
DYYYDYPKICEGRPKCDIDGYIYYDLYTIFGDCSKKLKIPNSCPSVVATSIADKLPLLLS
DDLPSNMTIASLVLSSQIVMKNEDNSTSVTHSLKENATLNFKLLKPINLTEYIPRCYYWD
TVENMWSDAGIETEVLTNDEVKCITSHLTSFAVLVDHQGLIGSASSSSSENATSISSTEL
QALSIVGYIGPSISLVCLIVALVIMILLRKKMNTTFLYFVHINLCISLVLALSVLIVGLE
TADHYSWLCSIVAGLLHYLFLCVFAWMLAEGITLCIMVTYVFEMKFLKWQIFLPAAWGIP
VLIVAVAAGIRYEQYGTENYCWLSTEKGLIWAFLGPALGVALVNTIFLIITVAQLVRVTC
SKVDTNNSKTSKNFKAAKSAIFGTIILFPLLGITWTIGVFGVSQDTTFFLWLFTICNSLQ
GVFILIFHVLKHKVVLTWLARRFPCLDRWIEVHTSAEASRRLSKQRLSVIHDPKLPKRRL
SDQFAFCLSSFLFLLWWSVKRKGRRTRIVIIINT

Frizzled like GPCRs

>PAC:15725169 pep:known
scaffold:Aqu1:Contig13501:51559:53230:1 gene:Aqu1.226641
transcript:PAC:15725169 description:""
MSFFYSLYLVSFFFIFYTPRVTGQCSSRTSLIQEATCNYNYTLRNLSIYPNQVINETTEI
SEVVTLVNALDSSLRSCSSNADRSGFICAAFFPSCDSGYGPCSSFCKKIRQDCSNTLPFL

PSTVSWIFNCTRYEPNSPCIQPISIEPTSSSSSTSSIVVISTPLPSQTPSTTPSTNESSV
GCPPPSSSYYTDGMKSFTKGWIATWSTLCFLSTSLTLLTFVIKPSRFEYPWRPIIFLALC
FNIHSLGYFFSLALGRGIITCPGNNYISSSSKWSWSHIPCLLNFGLLYYSMVSAFLWWLA
LTLSWFLSAVFKWSNESVGRLAPFFHVTAWVVPLLMMISLVAAQVISADELTGTCFIVRD
DSNATLFGLLIGVIIPLLLFLSIGLLFLIIGLISVFRIRRFLHNKGRQKESIVLEKLMFR
IGIFVAVYVLPAGAVISCYIYELAKVPDWVTLNEAESCSDGGCQKASPIVFIIRIFMFLM
IGILTGVWIWSKKTLDSWRSLGNSCCSNGSSGKNDQEPTMTKHVRSFSTVNS

>PAC:15726883 pep:known
scaffold:Aqu1:Contig13513:757187:759285:-1 gene:Aqu1.228355
transcript:PAC:15726883 description:""
MSLVILLFLAVVNLSTFMGQPTRPVSTLPPPGTCQPITGVAMCLYVPWGNASFPNLREHR
TQSEANTEVNVFQPLVTHQCSNAIVHFLCAVYTPACVEGPEGTVTLKPCRNLCNHVRNTC
EPLMQQGGLDWPPHFECSNFPESTGSTSDIDSLCFKVPINVTIPPELLPSATTSSGTAVQ
PTAATSSTFVSSMTLGTTPIPTATTIPSSQKCSPDLDLRRLGHEFANSSYTFASIPNCAV
PCEGIFFTETERNVVAPAFILVCALICIGFTLFTVGTFMIDRQRYHYPERPVIFLALCYL
ILSLAFVVGAIVKLSDPKSSFACSDTADSQSFVFQDLPNSTPTFHSASCVILFIITYYFQ
MAGAIWWVILTLTWFMASTLKWGEEAVERPWLLYHIFAWCIPAILVILLLSVRLVDGDQL
SGICYTGNSSSVSMGVFVLLPLLLFLILGIVFLVIGFGSLVHIHLQISKDPNKSRRLQRL
IIRILVYALLYIIPTFIYIFLCIYQLAERNSWEESYIKCPPSSFNCEPESKPQFTALLMR
YLVLFIIGIFSTFWVISWKTLLAWQKFFDSIFFCCHKREDYQLPRRNIETAI

>PAC:15726501 pep:known
scaffold:Aqu1:Contig13511:286703:287570:1 gene:Aqu1.227973
transcript:PAC:15726501 description:""
MSGLLSCSLTNASVLCSGKLIPYHSQASQPVFGTLHNCSEPCNGAFFSQEQVDILHYSNL
VLSCLSIITGCIILFLYLINYCKIKHPEAPIYYIAGLQLVISTMQLISGLYGTFNSMSST
NSSSDSEGLHCDDRYKNHFNESILLQDGGDNPFCLTIFAILYYSFLSILSWWTVITIEWL
VTSIKRKITKKKYLYIISHIAGWSIPIPFLFTAAYLNAVSGSTTEMTCWISRRNESLYQL
AFIITPLSDSGLIARKGDIVTAYISLQGMKKSPYLS

Other GPCR families

cAMP like GPCRs

>PAC:15717951 pep:novel
scaffold:Aqu1:Contig13368:29673:31585:1 gene:Aqu1.219423
transcript:PAC:15717951 description:""
MDESDTNSSSSSSCDEFDDLDYKIVSGVRAFSAFISLVSCLIVIILIFLFKRHHYFIQRL
VLYMCFMAAINSVTIMIQKVDYFTPDTNQRDALDKYCIFAGFAEYYTSAVELIGFVCITH
GLYRTVFKQKPKKYLELGYICISLLFPLLPACAPFFGFMYGKSGPWCWIRERSDNCDKET
VGIIFQFVLWYIPLVLLTVSTSIVYIAMFCKVHKSIESHWQGPYDPELFLHKGRLRKVVK
ILLAYMPILFLLINLMSLPNAIQWAIGEKPILALWILHGVFPPFRGALFAIPYLFHTDTR
QQIKQIGIRGAVRERFMRKKTVTSYPAKECNFSDSLTFPAAADTTYERNKPYRNPSLRQQ
ELSQRYSIPTIPQVATLSENTTSTSISTYSTDLGTHSSLSGLLTSRNAADTHSECESRNQ
QNE

>PAC:15717955 pep:novel
scaffold:Aqu1:Contig13368:37889:38992:1 gene:Aqu1.219427
transcript:PAC:15717955 description:""
MANNSSIDTSCFPTSHQLEIIGWVSIGTGGASLISCILILFVAILFQKYRGTTQRVILYL
TITVFLNSILYLLHGIRTILEGATDSDFCIALAFLDQVISWMEFLAILCLTVDFFVKSVF

LKFNTERFEIVYALVIFILPFTFNWVPFLGNTYGYGGTNCWIKQYKDMNCSETDKLGLAF
RFGLYWIPFYIIMTFVIIAYFISLFKARRRITQYSATTPTFSEQTIKELLLSEVRQYQLY
PFIMIVIFSLGIISRIAEAVDDGKNFFALRVFHVVVMAIQGPVIAIVFTMDYDTRKQICN
PRQILTACFGLWCCCRKNRVREYTAIIHEGSFISDPIMTPKQSSSMEEESLATGSGYITA
NKKNEVI

>PAC:15721723 pep:novel
scaffold:Aqu1:Contig13456:118682:119488:1 gene:Aqu1.223195
transcript:PAC:15721723 description:""
MATFCIVIALFIKAVCGRTTSQFEFGYVLIIFVFPLIFSWIPFYYDAYGPDDMFCWIREV
NLSDCNNYFKIGMILRYTLYYVPLTLLMLLLVALYIPAVIIAHKKKSRWSGNFDPEAIAI
RRMIAMEIRPLIYYPLIVLVINVFPLLKQLYATINDATSSEHDGLNHNLTTYFTLSVISY
VIYPFQGVLVTLAFTLDPETRKLLTWKRFVAAFLQTCCCRSRGIVRSYSIVHGRSDSLNY
GSTSRDRAAVTQDESCDPDGLHMKLNWE

>PAC:15721727 pep:novel
scaffold:Aqu1:Contig13456:123609:124673:1 gene:Aqu1.223199
transcript:PAC:15721727 description:""
MSNSNESNETDGPDLSCFDVFSDPRYTALAVMNIVFSISSILCLLVAISIIVLFKKYVHF
SQKLILYLCIASLFYAVSNCFNVTPPDVATNTASRGYCVVMGFLDQTVYWWMINCVAIIM
IDVFIRVAFERDTQKYKIPYIIGILVPPLVISWIPFLYLSYGPAGIFCWIRNKNYEDCST
FDIGNYFRFSVYYIPFYFLMLVLAVLLALSLCIARRKRKQWITGNLGKETKELQKKIASE
IRPLLGYPILFMIISIFPLISRIYDATEPDEGDVFYYILFVLLVTFYRLIGVTVSLIFVL
DQETRKHLNKKEIRAAYMRWRGLEKRHVTSYKFKVGQSDSFIDKSTAMTEENKQ

>PAC:15721726 pep:novel
scaffold:Aqu1:Contig13456:122275:123321:1 gene:Aqu1.223198
transcript:PAC:15721726 description:""
MSNDSSSNGTDQSCFDIYDDHHFIGLAVLSVVLSIISSIFLLAAIALIVLFKKYVYLSQK
LILYLCIASLLFSFSTVLNVTPADAHTNPVSKAYCIIMGFVDQVFLWWQINCVAIIMIDV
FIRVVFERNTRKYKIPYIIGIVAPPLVFSWIPFIGLSYGPAGVFCWIRDREYENCDVFNL
GAILRFSLYYAPFYALMIGLAILLVITLCISRRKKKQWMTGNLGKETIELQKKIISETRS
LIGYPVFFMVICITPLIFRIYGIFKTDGTIYFILSYLLVIVYRLIGVIIALIFALDPETR
KKLNCVEIQAAIQRWKGKEKNAVVAYEAEIGRSDSYSREKAIKIEKDT

>PAC:15721725 pep:novel
scaffold:Aqu1:Contig13456:121093:122136:1 gene:Aqu1.223197
transcript:PAC:15721725 description:""
MEFNSSTNCTMLDNEKYDGLVVVQIIFSTVSCVLLLCMIGLIVLFKEYQFFSQRLILYVA
VCSLIYSLINSINFFGREAQNASSALEDLCMAVGFFNQASFCWELMSVTIIMIDIFIKIV
FKRQTEKFELFYVILIIFVPLSFSWIPFIYKAYGPAGTFCWIRERTFNNETYCPLFWHGI
YLRFFLYYGPLYILFFALFILILSTWCIIRKRHKQWRNSQLDYQLVHLEQILEKEVRSLF
AYPVIFIVVNIIPFAHRVYGIYSTSDITYFVLSLITVILFSTQGSIIMIAFLSYPETRKK
MKWIEIKAAFRRWRGKEDAAIREYLVGESTERADSILPTNTEYTKIV

>PAC:15717952 pep:novel
scaffold:Aqu1:Contig13368:32110:33159:1 gene:Aqu1.219424
transcript:PAC:15717952 description:""
MSNNSSGVDLSCFTVYDNDGFVAVVAVRVALSIISILCCLGMLLIILLFKKYLFFAQRLI
AYLAIATLAYSVVSAINVEGYKAYRNDAIKGYCVFTGLLEQVVSWWVLLAISCIVFDLFV
KIMLKRETERFEWVYIIVTLVTPLITAWIPFINLAYGSSGAWCWIRGNDYDDCSSFTFGV
VLRFVLYYAPFYILMCALLIALVIVFVRLRKQRRSWTGNFDPTVLALKKKMSQEVFPLIS
YPIIFLVINIIPLILRIVNAVKSNEPIIALWFLSTIVFSLQGAVVMLAFALDPETRRNLA
PMQLLGAIKRQVTREGQVNEYEMGDDNEGEPEVVNSTSISFPSYPDEDQ

>PAC:15716961 pep:novel
scaffold:Aqu1:Contig13340:36565:38756:1 gene:Aqu1.218433
transcript:PAC:15716961 description:""
MVNFPCPLFPNNTGYELSVVDIRVSVSCFSLLGSIFVIGLIWLFKKYKFFTQRLILYLSI
ASLISSIAYILGRFDYGSDNKQHLCIFTGFLLLYSDWSVLLSTIGVTANLFFKVTCERLS
NKYEFIYVMLSFGVPVLFIWIPFTFDAYGPSGAWCWIRNKNVTDCTPFIKGKIIQLLVWY
IPLYALILFLFIVYVYIAARVSYRVRTWQGLYDPQVQRQRILMREEIKPLMWYPLIYFLV
QLFPFINRIVYNVTDNAYIGLFVLHVLTSPLTGVLIAFAYALDKETLKRLNWNSIKASLR
FRSTSKIVTAYSAEETFEEELEATPRSTYFSESSEGEDENKRLVSTRID

>PAC:15722414 pep:novel
scaffold:Aqu1:Contig13467:307811:308695:1 gene:Aqu1.223886
transcript:PAC:15722414 description:""
MNNHTCLFLSLNEVRTLGIIRSSVALVSALSCIGAIVLVLLLARKQWRRFKFTKRLSLYL
TILALLYSVATTFQWVSSSAIDEQPGGEQGCKAVAFFVQYSSWSLLVFTFLLTLILFITV
YKRKEPHYKRWEIGTLLASILSPLITVWIPFINDSYGLSGIWCWIRSHDSTNCTVFLPGV
IEQIALWYAPLSILMIASSILMFMTIGKYIYLVFKPCGYQTIDADLRYQNSFKDYSPLVV
YPIVFFSLNIIALSSRIASTQYVFPKGLLYIHSIVDPCWGLIASIATIIYSVKF

>PAC:15726659 pep:novel
scaffold:Aqu1:Contig13512:397833:398765:-1 gene:Aqu1.228131
transcript:PAC:15726659 description:""
MIFCRLFTLLTHRLIIYMLLGIFFYSFIVAAQCMNYWLNIWKGEHAVVCIVESYFTEYAC
WVMLLSVLMVTLHLTVMVLFPKFHIKMGRLEPFYILFPWLFPLLVAWIPFVHNNYGISGS
WCWIRLYNNNCSLNKEGMIEMYAVLYGELFVGLILNNIALIIIFVTLCKRVYCNTTCPNY
RKTLKQTLPLVIYPISYQLFTWFAIANRLNQALNKGSGGTWLFYMHAFFGASGGFFAPLL
SLLYILFLRKTIKDNIRKWHCLKCFFGPKTESEATDTSRLINPDQPERASSTNTNQPRES
EIEREYEIIN

>PAC:15727835 pep:novel
scaffold:Aqu1:Contig13519:507890:509017:-1 gene:Aqu1.229307
transcript:PAC:15727835 description:""
MEELDEENATTNHTHSGLLSSEQKYIILLTMCVLSIISIIICLIAMVMAVKFKLHKYFVH
RLAIYQVLSAMLYDAVLALEVVFINYDTKKSVYYPVCVVEAFLIEYSIWIKLLFTFCLTF
HLFCFSVFHVNLNRLEIVYILVSTLGPALFTWVPFVNGLYGLAGAWCWIQNWKGDNALKK
LPQGEIEQYTLLYGPAIFCLLLCGVAVVIVTAILVHRAYNCNCCNNKIHDDDDNENQPLL
KEQLTEQKKKVLREMLPLVSYPILSLMFYIPAFINRLVGSISQSPNFISFMWSGVSLPLI
GLLAGVSLIVHIFILEHPKRRSTIKKRRQLVDVTQRIKSINDVFTTDTVASTAAKTHFEV
PPESAIDEELNPSMN

>PAC:15721256 pep:novel
scaffold:Aqu1:Contig13448:35351:36343:-1 gene:Aqu1.222728
transcript:PAC:15721256 description:""
MEDDSPCNSSQAQFSNQQKAILLYIYGGVGVISTLCCVFALIVAVRYRFYQDIVQRLVLY
QLIAALAVSLICAFDIIFVNYHKNPDVYQPFCAMAAYFNTVSLWTKIGFTFWLSLLLFIC
LVYLKQPKDVKKLEPLYILTSFGIPMLFSWIPFANDLYGISGAWCWIKNWEGECANKKIL
LGTIEQYALFYAPATVFFLIDAILITATVIVLLKRIRRNNVSEEQLLLGENRKQKQLLRM
ILPLSVYPVVSIFLYIFPFSNRVYSIIDDKPIFPLFILHGVTEPLWGCCVGIVVTVHICI
AKSKRSSSGDTGMRSVTDHYTLPKDSMSFE

>PAC:15719005 pep:novel
scaffold:Aqu1:Contig13399:199087:200235:1 gene:Aqu1.220477
transcript:PAC:15719005 description:""
MSGDCPNGSDLTSEEKRIILSVYGGEGILSATVCLTALLMIIKLKYYKDTVQRLLLWQLI

AGLGLSLYNMLNLLFINYDEETSSRAYTVLCSIVGYIGTVTFFVKIAFTLWLTIFLFRCL
VCLKDPKELSKMEILYVSTSLGLPLLVSLVPFIHHSYGIAGAWCWIKEWKDDCASDEKLV
LGVIFEDAFYSSGAVIALTNAVLITITLSTVLCRIRCSCVGSSSSEKEQLLETVQLKHKN
LLKKLLPLIAYPLVFLFLIIFPLTNRIYGGIGKRISFPLFMLHAAVEPFWGLFVGVAIIV
HIAVIEYKKKTKKIHFKVPVVTGVQSMRRYNALSPVDDAYTVDTVAATSAKTTFEPSTES
IDNCSCTDDNINGSIVSTDVYK

>PAC:15717950 pep:novel
scaffold:Aqu1:Contig13368:28057:29224:1 gene:Aqu1.219422
transcript:PAC:15717950 description:""
MIGGADIPEDNVIPICYKFDNSQARRIGVARAASGISTLIVSLVAIVIILSMKAIKQYPQ
RLFFYLTLATLIQAPTFTLEVLSVSYNRSESEQPLCSIAGVYTMYTSWLQNLIILWISVY
LVRVIVLRLTVYNKKLEVFLLILFLVLPIPGALIPLIKNKYGMVGAWCWIKDFNSTDCVR
HDNLGIAYQYTLWFIPVMLEVVIIAIVTVLIGAVLCVRGFIAKKYVLFQSEYRKLLRDSI
PLLCFPLFFCSISCFELILYWSSLDAEPKYNLWMLNAILTPCKGILMIFGYLIPMIWIRI
KDYRAKKGRLVLRKEDWRSDNNSSFLDTVSIRFQSSGGEDHYGSVSVYSTEIKN

Ocular albinism (GPR143) like GPCRs

>PAC:15722473 pep:novel
scaffold:Aqu1:Contig13468:153476:155508:1 gene:Aqu1.223945
transcript:PAC:15722473 description:""
MASPHASSFMCLPFDSMREVSDAYITYYGVCLGSGCIGTLGSMLFLYQVLRGAAAKSTSK
TQKNILINLAFADLLADIGVIGRSLYYMVYWRDHDLTKIDLRDRLPQAITGAFTESWVVF
WYLASYFWTFFFAVDIYTAKHKGRWRLWCSHMVTWLYCSLLAATGTLALWACAPPLECAV
DKYCIVGHFLCTFGPILIFLVSLPVFYFSILKKIIDEFKDDGHALEVREREKKQAKKKIL
LFIIVFYFCWLVNVVDGFILVIYHLVEKGALAPSVRQTFLYKPAYIYTVWIIEAAVNPLQ
GLLNAIAYGNACQSVHECYVQVKAKLTASLSHQASFSGEHIEVDFREGRGSVWTTQRKRV
LSC

Intimal thickness-related receptor like GPCRs (GPR180 like)

>PAC:15717891 pep:novel
scaffold:Aqu1:Contig13365:154501:156037:-1 gene:Aqu1.219363
transcript:PAC:15717891 description:""
MSSFMLFLLLFLSSSLHLAFSLRIKGSYDVSNETLHKEGQMKYKFLDKFGIFKGDSVNLY
GSVNITGDLLFAFIKKKVWDKFHPGSSCDQLQTQSLRYNNRFVQPLPPPNPSSDLTPVSS
SSSPLSPSASHSISMNTEMNESTDTEFWFAVFIYCRESNNAWMIPNDSLSLSYEIFIVNA
NHDHNNDTSPFTRQFPGNEKGFLFTYLLFSVFYVILIPVHLLSHLKYTIKCRTPQIINLF
SLALVFEGINIFCGLIHYGIYAHNGYGAPPLNYMKTFFNLVGDWFLIIVLVLIAGGWMVT
LRTVKWRLASFIFISLYIFVTIIYYIATVIYMNVQPWDTLHPLQEWPGGIYLFARSLLLF
YVWYQIYHIHRQEETLQKLRLYRALFGIFTVWFWYLPITVFIVTLINPVVSWVVLINIMN
FMNFIVNVVMVALFCPFWSHEYFQFQFNVETSSMTSALMNSSSYTKKYQMMSGNSYDKRE
DKDLIL

>PAC:15726074 pep:novel
scaffold:Aqu1:Contig13508:509929:511865:-1 gene:Aqu1.227546
transcript:PAC:15726074 description:""
MAFSWIFLVVALLHFLFIETRAKVIAGVVSSGQAWSENGTFITKFCFHDRRALFEYETNI

SSNGRWYFYLDDHWSDSLAETDCRNKIAKARFSVGISATRGQQLVNQWLRPHFWYVVYAD
PNTCDESPPTEDLFIGYTLTFLNPDSEGNANDHFGEDEKDLRMTVKEVQLVKRYPKVILV
LWEQSYEESHYTYHVHENLPGFLLVCLRIILAVLFGYNLQSTLSKERSTLRKDFYQAFAI
ICYLWFLSFPMLMLIAMVLPEYWRHRVSHYYNYQYFSHE

Lung 7TM receptor like GPCRs (GPR108 like)

>PAC:15721385 pep:known
scaffold:Aqu1:Contig13450:242450:247434:1 gene:Aqu1.222857
transcript:PAC:15721385 description:""
MLWQPVNNLLINAHQEIPRPLIGVVSHLSYVSKSLSSSSVFALLLSSFSMLRSVFLLLCC
LSLSLAVPDHGHNKVVLFNNTQTRSGCTSLTMAGGSRIRVSLYNANMSAHEAVITFKITL
YESQCEAIDKKNCAIRTVDEIVMTNTQVFKVTSGRDYINFHSQKKPSSAANSTNTKNGTA
TTASKRSAASTEVTAAPTDGATTKAPTDGATTKAPNDGTTTKAPNDDTTTKAPNDDTTTA
APTNGTTTTAPTTPPPTEAPTTTVLINNASSVPDDPEWATFTTNNSGVFDVVIEATAMKN
DINFSLAFHVDMKNKYGYLSLVDYPAMIVYSILMILYIFYAILWLVLMALEYKDLVRLQF
WILAVILLGFLEKVFFVAEYGTANGGIDSSGLIYVAEIVSAAKRALSRVLIIIVCEGFGT
VKPRLGHLLTKVVFLGFVYFIVAFIDGVLRASKGIGNHGLTTMLTFGLLFLLDTGIVYWI
FSALINTRRNLRVRKNTVKLALYNHLGYALIATVIASLTFIIWQFIYITRSNCTNDWREM
WLIECFWHMLFCFMLLIVMIIWRPSANKLRFAYSPVSNGDSDEEEQGANKNFETVKMRPV
SGKSESTKQQLTSMEAEEDLKWVEQNLPTTAIDKAAQMVLDSEEEVMTTRFEASKLN
