# Supplementary material for: The GPCR repertoire in the demosponge Amphimedon queenslandica: insights into the GPCR system at the early divergence of animals
Source: BMC Evol Biol. 2014 Dec 21;14:270. doi: 10.1186/s12862-014-0270-4 (PMC4302439; doi:10.1186/s12862-014-0270-4)

Rhodopsin GPCRs in Sponge and *Trichoplax*

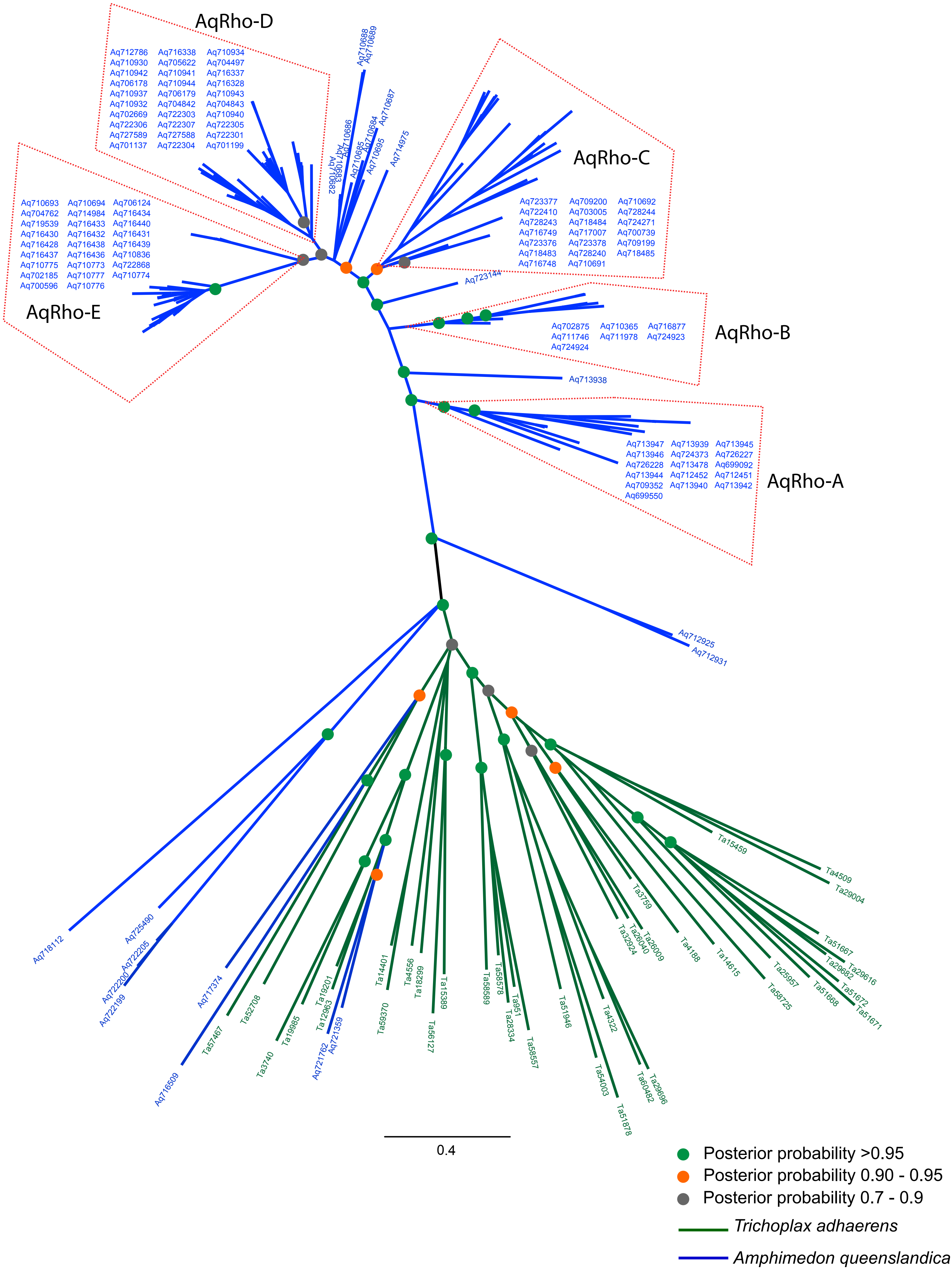

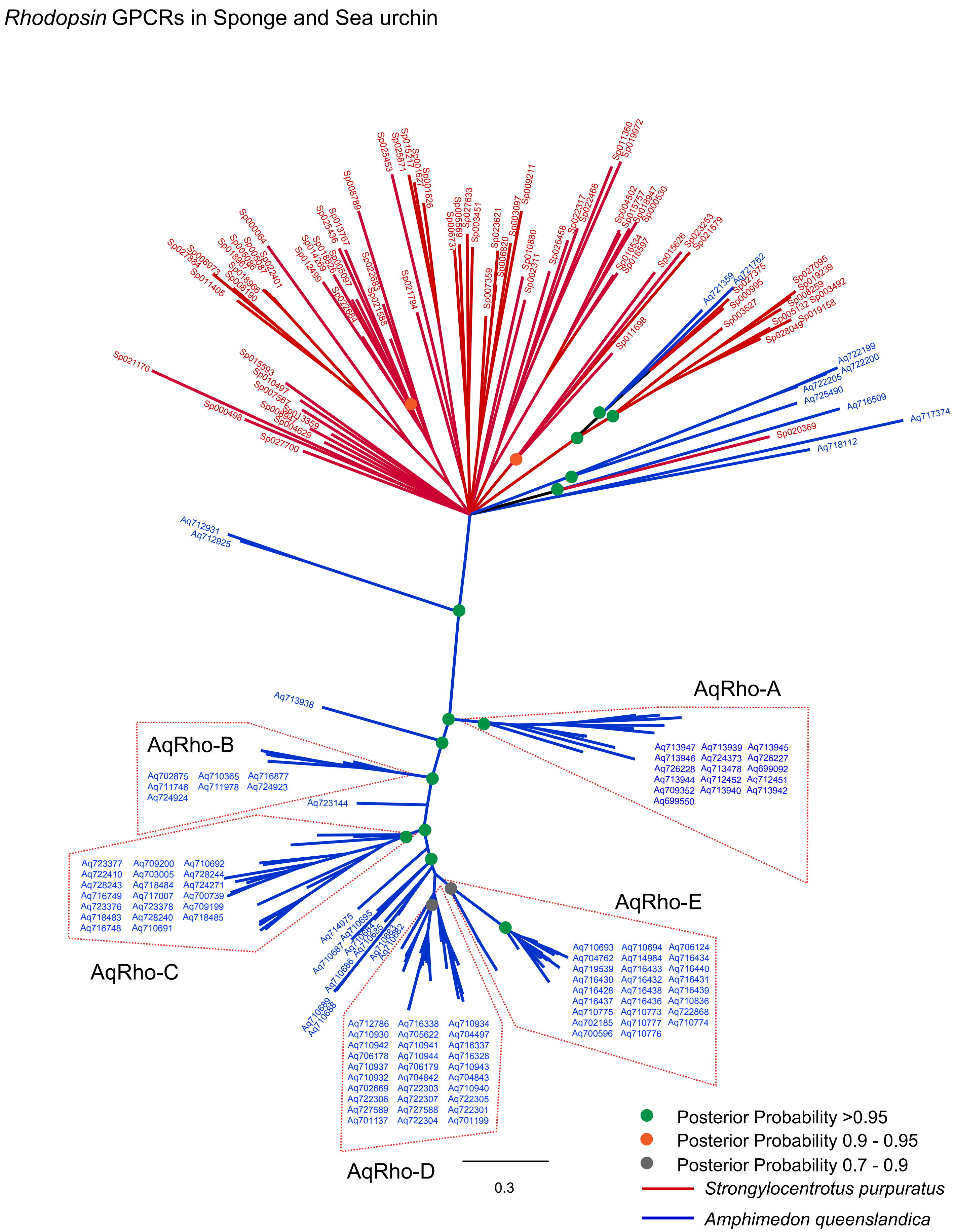

Rhodopsin GPCRs in Sponge and Sea urchin
AqRho-A
Aq713947 Aq713939 Aq713945
Aq713946 Aq724373 Aq726227
Aq726228 Aq713478 Aq699092
Aq713944 Aq712452 Aq712451
Aq709352 Aq713940 Aq713942
Aq699550
AqRho-B
Aq702875 Aq710365 Aq716877
Aq711746 Aq711978 Aq724923
Aq724924
Aq713938
Aq723144
Aq712931
Aq712925
AqRho-C
Aq723377 Aq709200 Aq710692
Aq722410 Aq703005 Aq728244
Aq728243 Aq718484 Aq724271
Aq716749 Aq717007 Aq700739
Aq723376 Aq723378 Aq709199
Aq718483 Aq728240 Aq718485
Aq716748 Aq710691
AqRho-E
Aq710693 Aq710694 Aq706124
Aq704762 Aq714984 Aq716434
Aq719539 Aq716433 Aq716440
Aq716430 Aq716432 Aq716431
Aq716428 Aq716438 Aq716439
Aq716437 Aq716436 Aq710836
Aq710775 Aq710773 Aq722868
Aq702185 Aq710777 Aq710774
Aq700596 Aq710776
AqRho-D
Aq712786 Aq716338 Aq710934
Aq710930 Aq705622 Aq704497
Aq710942 Aq710941 Aq716337
Aq706178 Aq710944 Aq716328
Aq710937 Aq706179 Aq710943
Aq710932 Aq704842 Aq704843
Aq702669 Aq722303 Aq710940
Aq722306 Aq722307 Aq722305
Aq727589 Aq727588 Aq722301
Aq701137 Aq722304 Aq701199
0.3
Posterior Probability >0.95
Posterior Probability 0.9 - 0.95
Posterior Probability 0.7 - 0.9
Strongylocentrotus purpuratus
Amphimedon queenslandica

Rhodopsin GPCRs in Sponge and *Nematostella*

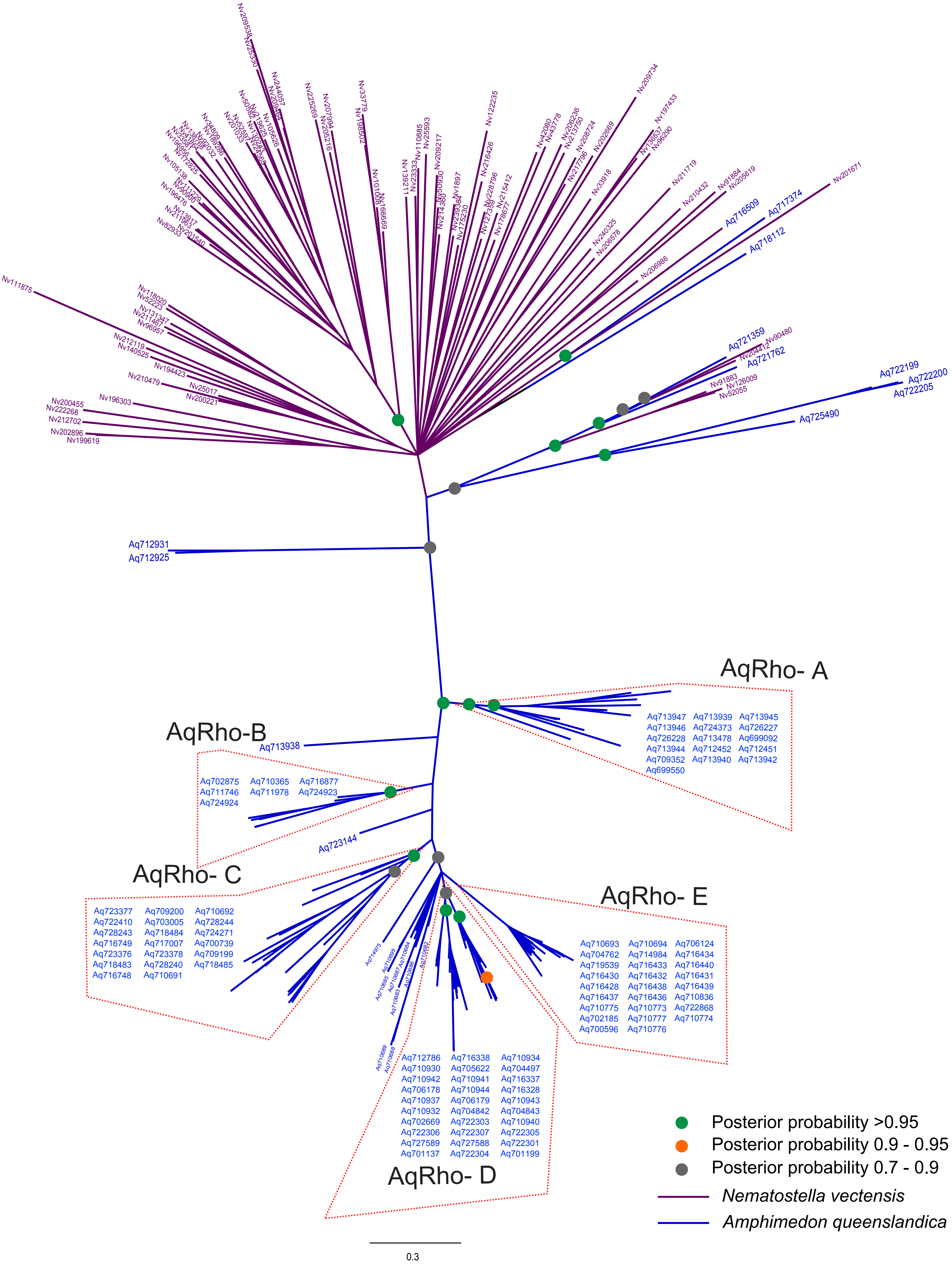

Supplement: Additional file 4: — Phylogenetic relationship between Rhodopsin GPCRs in sponge and other metazoan genomes. [file 12862_2014_270_MOESM4_ESM.pdf]
