# Supplementary figures and images for: The GPCR repertoire in the demosponge Amphimedon queenslandica: insights into the GPCR system at the early divergence of animals
Source: BMC Evol Biol. 2014 Dec 21;14:270. doi: 10.1186/s12862-014-0270-4 (PMC4302439; doi:10.1186/s12862-014-0270-4)

# Adhesion GPCRs in sponge and human

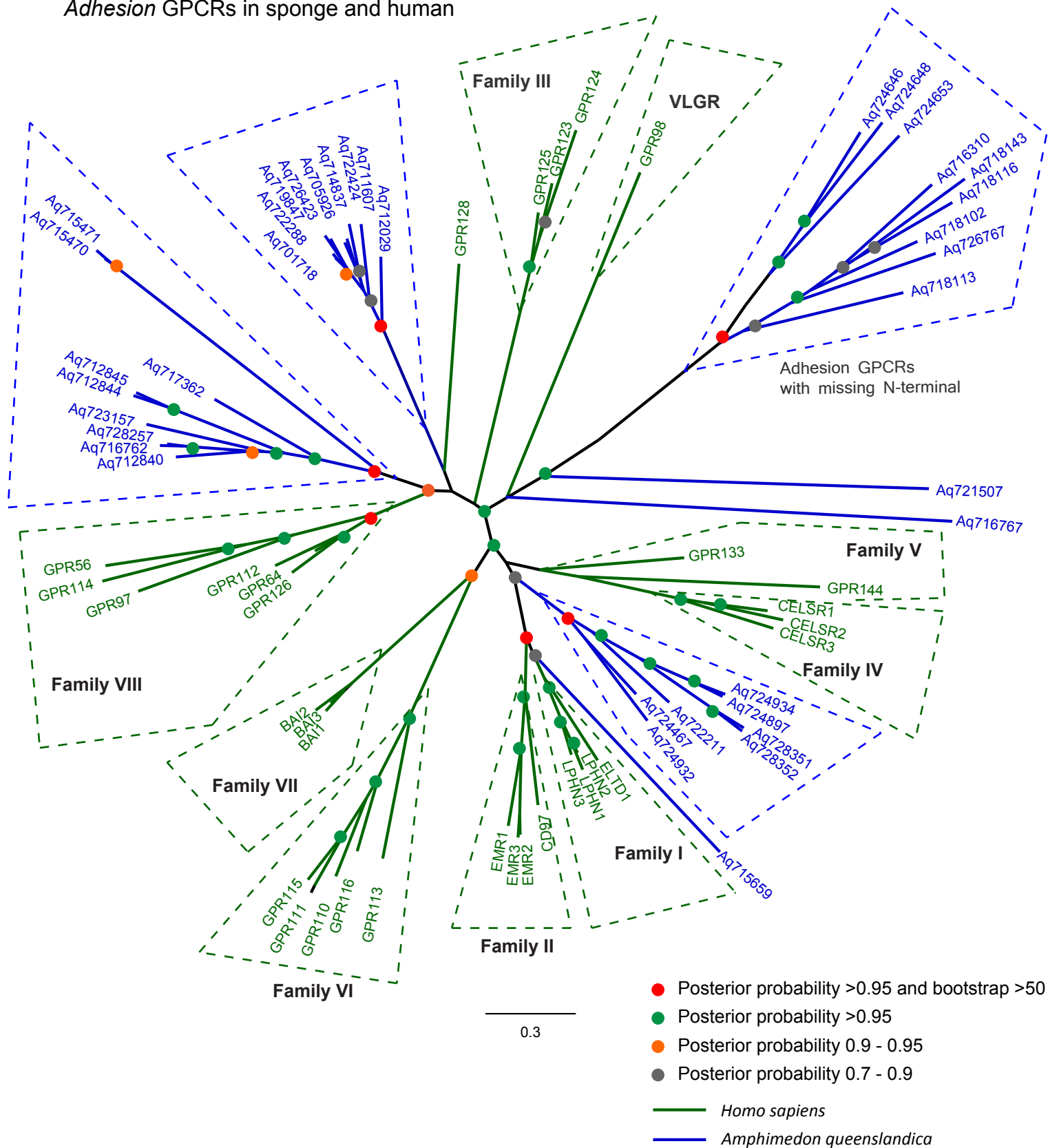

Supplement: Additional file 5: — Phylogenetic relationships between Adhesion GPCRs in sponge and human. [file 12862_2014_270_MOESM5_ESM.pdf]

## Glutamate GPCRs in sponge and other genomes

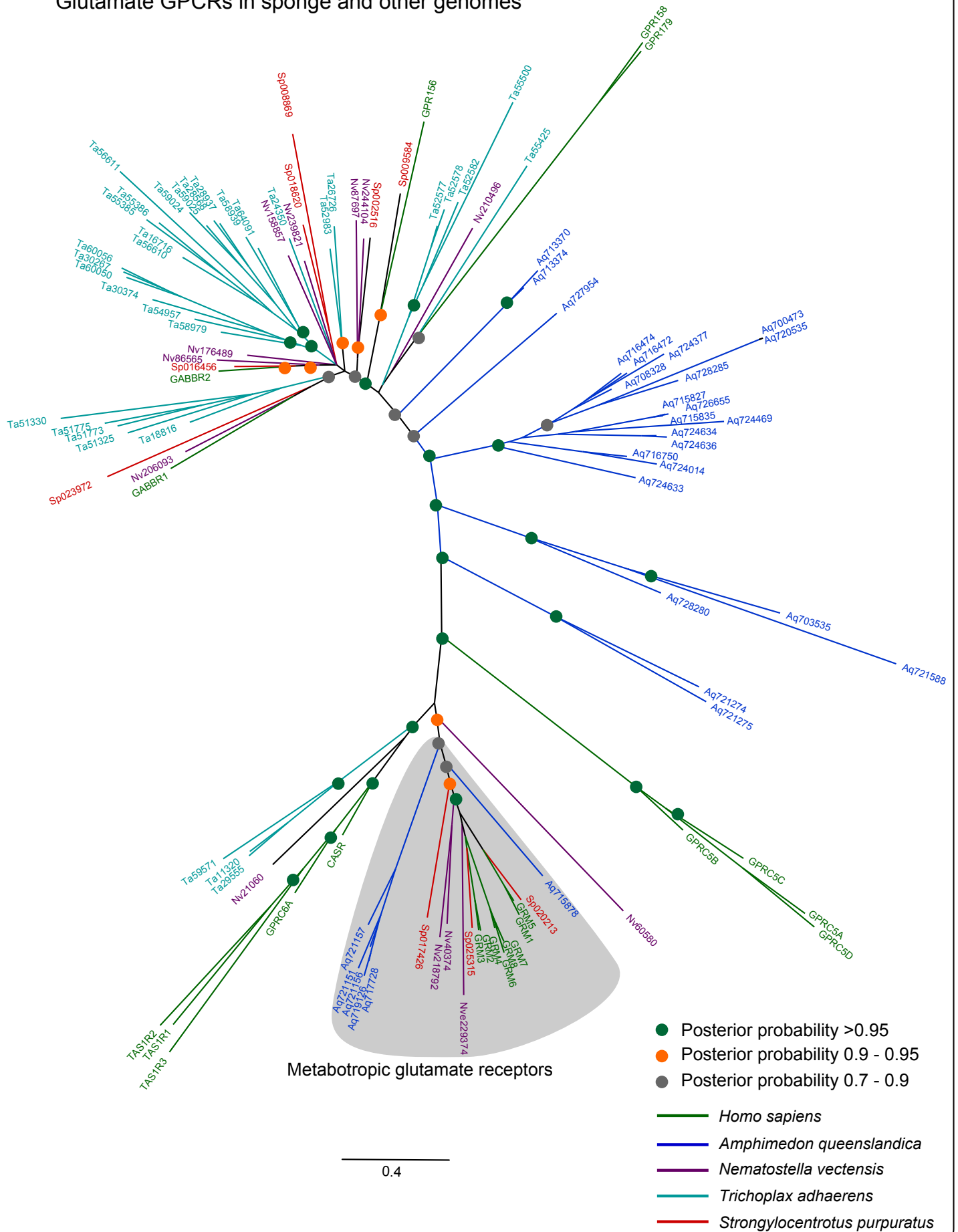

Supplement: Additional file 6: — Phylogenetic relationships between Glutamate GPCRs in sponge and other metazoan genomes. [file 12862_2014_270_MOESM6_ESM.pdf]

Frizzled GPCRs in sponge and other genomes

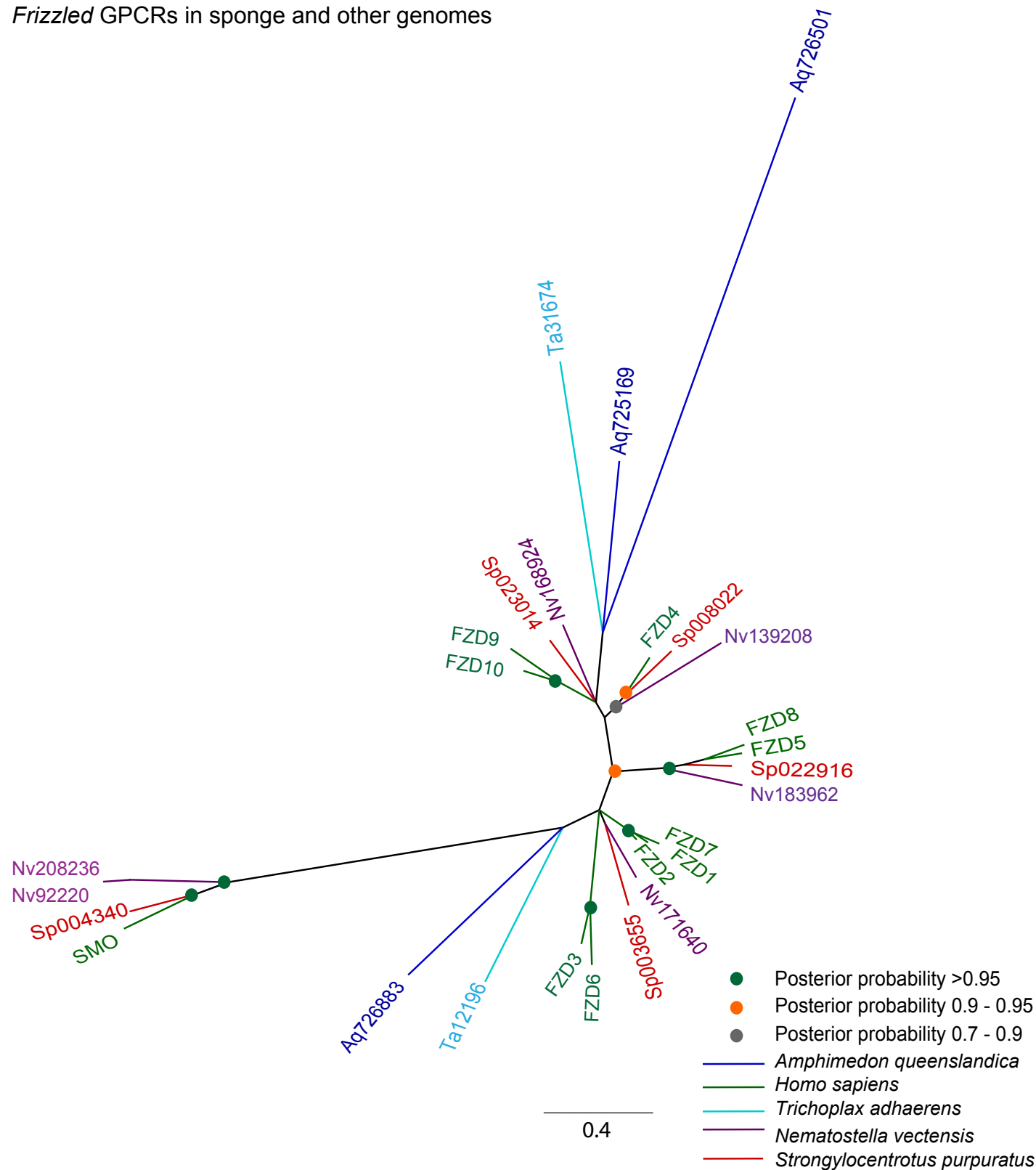

Supplement: Additional file 7: — Phylogenetic trees showing relationships between Frizzled GPCRs in sponge and other metazoan genomes. [file 12862_2014_270_MOESM7_ESM.pdf]

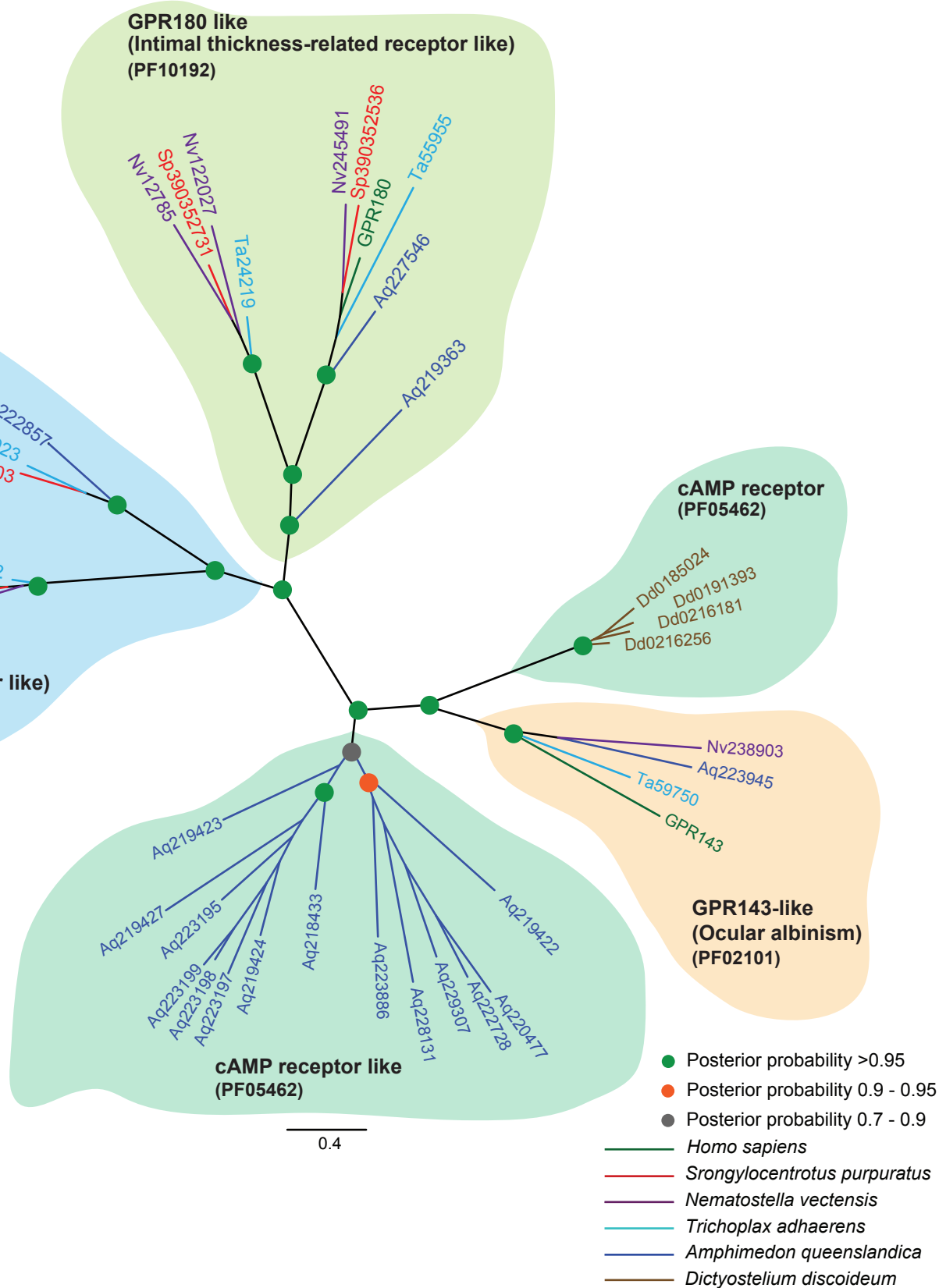

Supplement: Additional file 8: — Phylogenetic tree showing relationships between ‘other’ GPCR families (cAMP-like, intimal thickness-related receptor like (ITR-like), lung 7TM receptor-like and ocular albinism like GPCRs) in sponge and analyzed metazoan genomes. [file 12862_2014_270_MOESM8_ESM.pdf]
